# p53 promotes peroxisomal fatty acid β-oxidation to repress purine biosynthesis and mediate tumor suppression

**DOI:** 10.1038/s41419-023-05625-2

**Published:** 2023-02-07

**Authors:** Jianhong Zhao, Xiaojun Zhou, Baoxiang Chen, Mingzhu Lu, Genxin Wang, Nagarajan Elumalai, Chenhui Tian, Jinmiao Zhang, Yanliang Liu, Zhiqiang Chen, Xinyi Zhou, Mingzhi Wu, Mengjiao Li, Edward V. Prochownik, Ali Tavassoli, Congqing Jiang, Youjun Li

**Affiliations:** 1 Hubei Key Laboratory of Cell Homeostasis, College of Life Sciences, Frontier Science Center for Immunology and Metabolism, TaiKang Center for Life and Medical Sciences, Wuhan University, Wuhan, 430072 China; 2 Medical Research Institute, Zhongnan Hospital of Wuhan University, Wuhan University, Wuhan, 430071 China; 3 Department of colorectal and Anal Surgery, Zhongnan Hospital of Wuhan University School of Medicine, Wuhan, 430071 China; 4 School of Chemistry, University of Southampton, Southampton, UK; 5 Department of Gastrointestinal Surgery, Renmin Hospital of Wuhan University, Wuhan, 430060 China; 6 Division of Hematology/Oncology, Children’s Hospital of Pittsburgh of UPMC, The Department of Microbiology and Molecular Genetics, The Pittsburgh Liver Research Center and The Hillman Cancer Center of UPMC, The University of Pittsburgh Medical Center, Pittsburgh, PA 15224 USA

**Keywords:** Cancer metabolism, Colon cancer

## Abstract

The metabolic pathways through which p53 functions as a potent tumor suppressor are incompletely understood. Here we report that, by associating with the Vitamin D receptor (VDR), p53 induces numerous genes encoding enzymes for peroxisomal fatty acid β-oxidation (FAO). This leads to increased cytosolic acetyl-CoA levels and acetylation of the enzyme 5-Aminoimidazole-4-Carboxamide Ribonucleotide Formyltransferase/IMP Cyclohydrolase (ATIC), which catalyzes the last two steps in the purine biosynthetic pathway. This acetylation step, mediated by lysine acetyltransferase 2B (KAT2B), occurs at ATIC Lys 266, dramatically inhibits ATIC activity, and inversely correlates with colorectal cancer (CRC) tumor growth in vitro and in vivo, and acetylation of ATIC is downregulated in human CRC samples. p53-deficient CRCs with high levels of ATIC is more susceptible to ATIC inhibition. Collectively, these findings link p53 to peroxisomal FAO, purine biosynthesis, and CRC pathogenesis in a manner that is regulated by the levels of ATIC acetylation.

## Introduction

As a master tumor suppressor, p53 inhibits proliferation and tumorigenesis in a variety of ways that include the induction of cell cycle arrest and the promotion of apoptosis and senescence [1, 2]. More recent studies have uncovered additional roles for p53 in metabolism, including the regulation of glycolysis; the pentose phosphate pathway; mitochondrial oxidative phosphorylation, and amino acid, nucleotide, and lipid biosynthesis [2]. Accumulating evidence suggests that this metabolic regulation is a key aspect of p53’s diverse tumor suppressive activities [3], but many questions remain unresolved.

Dysregulation of fatty acid β-oxidation (FAO) is a frequent feature of cancer metabolic reprogramming [4]. FAO normally occurs in both mitochondria and peroxisomes although the latter is a non-energy-generating pathway and makes preferential use of very long chain fatty acids (VLCFAs) (i.e., >C18) [5–7]. Previous research has shown that p53 enhances mitochondrial FAO [2, 8] by increasing pantothenate kinase 1 (PANK1), which supplies the Coenzyme A (CoA) required for the biosynthesis of acetyl-CoA, the final product of the FAO catabolic pathway [9, 10]. p53 also increases mitochondrial FAO by transcriptionally inducing several other key enzymes in the fatty acid degradation pathway, including CPT1C [11], Lipin 1 [12], Acda11 [13], and MCD [14].

In addition to its role in energy generation, acetyl-CoA is a donor of acetyl groups for histones and other proteins [9, 15]. For example, peroxisome-derived acetyl-CoA is a major source of hepatic cytosolic acetyl-CoA, which contributes to the acetylation of Raptor (regulatory associated protein of mTOR Complex 1), a key regulatory node in autophagy pathways [16]. These results underscore the importance of acetyl-CoA derived specifically from peroxisomes in protein acetylation.

Purine metabolism provides cells with critical anabolic cofactors needed to sustain survival and proliferation and is particularly critical in rapidly dividing cancer cells [17, 18]. However, the mechanisms by which purine biosynthetic balance is achieved and maintained remain poorly understood. 5-aminoimidazole-4-carboxamide ribonucleotide formyltransferase/IMP cyclohydrolase (ATIC) is a key metabolic enzyme in purine metabolism that catalyzes the last two steps in this pathway [19]. Here, we report that p53-mediated increases in peroxisomal FAO, results in increasing acetyl-CoA levels. p53 also induces KAT2B expression. This leads to increase KAT2B-mediated ATIC acetylation to deactivate its enzymatic activity, reduced cellular purine biosynthesis, and tumor growth inhibition. Our study thus identifies a previously unrecognized link between the p53-dependent generation of peroxisomal acetyl-CoA and tumor suppression via a pathway involving the acetylation-dependent regulation of ATIC and the final steps in purine biosynthesis.

## Results

### p53 facilitates peroxisomal β-oxidation

**Fig. 1:**
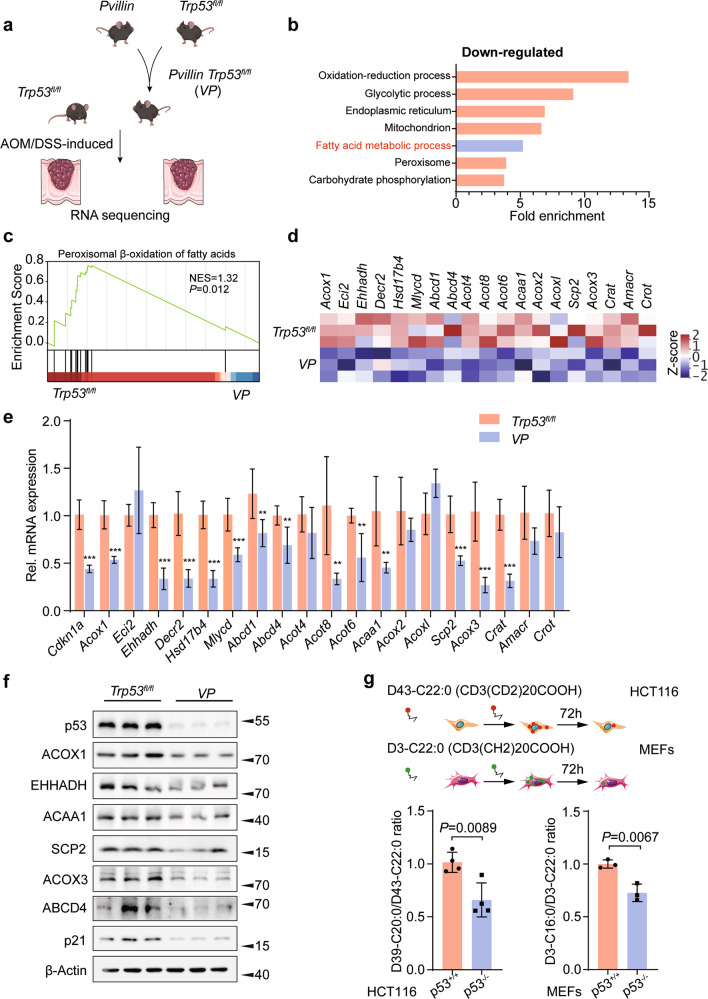
p53 facilitates peroxisomal β-oxidation. **a** Experimental scheme. *Trp53*^*fl/fl*^ mice were crossed with *Pvillin-Cre* mice to generate *Pvillin-Cre* + *Trp53*^*fl/fl*^ (*VP*) mice, and the colorectal tumor model was induced by AOM/DSS. **b** Gene ontology analysis of gene sets that were significantly downregulated in metabolic process from *VP* mice CRC tissues compared to *Trp53*^*fl/fl*^ ones. **c** Gene set enrichment analysis (GSEA) was used to evaluate changes in the gene signature of peroxisomal FAO comparing CRC tumors from *Trp53*^*fl/fl*^ and *VP* mice. **d** Heatmap of RNA-seq expression values of peroxisomal FAO genes comparing tumors from *VP* and *Trp53*^*fl/fl*^ mice. **e** qRT-PCR analysis of various peroxisomal FAO genes comparing CRC tumors from *Trp53*^*fl/fl*^ and *VP* mice, *n* = 6. **f** Western blot analysis for ACOX1, EHHADH, ACAA1, SCP2, ACOX3, ABCD4, and p21 expression in CRC tumors from AOM/DSS-induced colitis-associated colorectal cancer (CAC) mouse model, *n* = 3. **g** HCT116 cells and MEFs were incubated with D43-C22:0 or D3-C22:0, whose catabolism to D39-C20:0 or D3-C16:0 was measured using mass spectrometry. Peroxisomal FAO was expressed as the ratio of D39-C20:0 to D43-C22:0 or D3-C16:0 to D3-C22:0; *n* = 3 or 4. Data were presented as mean ± SD. **P < 0.01, ***P < 0.001.

After crossing *Trp53*^*fl/fl*^ mice with *Pvillin-Cre* mice to generate *Pvillin-Cre* + *Trp53*^*fl/fl*^ (*VP*) mice, colorectal cancers (CRC) were induced by AOM/DSS (Fig. 1a) in both cohorts. On day 98, the *Trp53*^*fl/fl*^ and *VP* mice developed large tumors in the colon (Fig. S1a). Then, tumor tissues from CRC mice were analyzed by RNA-seq and Gene Set Enrichment Analysis (GSEA). We found p53 knockout in the *VP* group to be associated with marked down-regulation of genes involved in fatty acid metabolism and more specifically, peroxisomal β-oxidation (FAO) (Figs. 1b and 1c). Transcriptomic analysis of GSE27901 [20] revealed similar findings when comparing p53 WT and p53 null mouse embryo fibroblasts (MEFs) (Figs. S1b and S1c). Notably, neither CRC tumors nor MEFs were enriched for gene sets pertaining to mitochondrial FAO (*P* = 0.435 and *P* = 0.167, respectively) (Fig. S1d). Among the peroxisomal FAO transcripts that were more highly exp*ressed in Trp53*^*fl/fl*^ CRCs were those encoding *Acox1*, *Ehhadh*, *Acaa1*, *Scp2*, *Acox3,* and *Abcd4* (Fig. 1d). Similar gene expression differences were observed between WT and p53 null MEFs (Fig. S1e). We confirmed increased mRNA and protein levels for selected genes in CRC tumor tissues from *Trp53*^*fl/fl*^ mice (Figs. 1e and 1f). Upregulation of the above genes was confirmed in HCT116 CRC cells following treatment with Nutlin3, which reactivates p53 protein (Figs. S1f and S1g). Similarly, knockdown (KD) of WT p53 with two different shRNAs significantly reduced levels of selected peroxisomal FAO proteins in HCT116 p53^+/+^ cells (Fig. S1g). The depletion of p53 in WT MEFs also led to a marked down-regulation of peroxisomal FAO proteins (Fig. S1g). Notably, the defect could be rescued by expressing WT p53 in HCT116 p53^−/−^ cells (Fig. S1g), but not in cells stably expressing various p53 mutants (Fig. S1h).

The foregoing results pointed to a direct positive correlation between p53 and peroxisomal FAO gene and protein expression. To more closely explore the physiologic implications of this, stable isotope–labeled docosanoic acid D39-C20:0/D43-C22:0 and docosanoic acid D3-C16:0/D3-C22:0 ratios were determined by mass spectrometric (MS) analysis [21]. This revealed significant increases in the levels of peroxisomal FAO in the HCT116 p53^+/+^ cells and MEFs cells compared with their p53^−/−^ counterparts (Fig. 1g), thereby confirming at the metabolic level that p53 accelerates peroxisomal FAO.

### p53’s association with the Vitamin D receptor is necessary to promote peroxisomal FAO gene expression

**Fig. 2:**
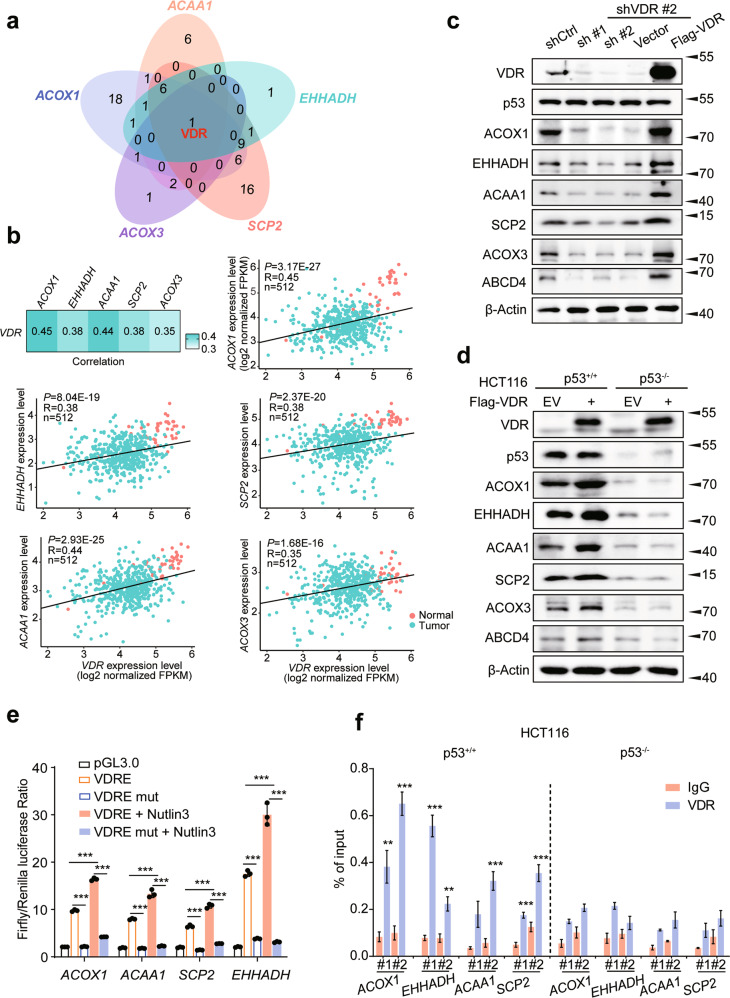
p53 is associated with VDR to promote the expression of genes involved in peroxisomal β-oxidation. **a** Venn diagram showing the numbers of overlapping TFs among the peroxisomal FAO rate-limiting enzyme genes. **b** Correlation between the expression levels of *VDR* and *ACOX1*, *EHHADH*, *ACAA1*, *SCP2*, *ACOX3* in 41 normal (orange) and 471 tumor (green) COAD samples, as determined by Pearson’s *r* analysis. **c** Protein expression of ACOX1, EHHADH, ACAA1, SCP2, ACOX3, and ABCD4 in HCT116 cells with VDR inhibition. **d** Western blot analysis of ACOX1, EHHADH, ACAA1, SCP2, ACOX3 and ABCD4 expression in HCT116 p53^+/+^ or p53^−/−^cells expressing VDR. **e** Dual-luciferase assays in HEK293T cells transfected with the indicated plasmids or treated with Nutlin3 (10 μΜ), *n* = 3. **f** qPCR ChIP analyses of VDR binding to *ACOX1*, *EHHADH*, *ACAA1*, and *SCP2* promoter regions in HCT116 p53^+/+^ or p53^−/−^cells, *n* = 3. Data were presented as mean ± SD. **P < 0.01, ***P < 0.001.

To investigate the mechanism(s) by which p53 promotes peroxisomal FAO gene induction, we determined whether the *ACOX1, EHHADH, ACAA1, SCP2*, and *ACOX3* genes are direct p53 targets. Thus, we first analyzed ChIP-seq data from HCT116 WT cells treated with Nutlin3 [22] but were unable to identify any potential p53 response element binding (data not shown). Using the Cancer Genome Atlas (TCGA) database to analyze correlations between peroxisomal FAO genes and 1639 human transcription factors (TFs), we found that only the expression of the vitamin D receptor (VDR) positively correlated with the expression of all the peroxisomal FAO enzyme genes (Extended Data table 3, *R* > 0.3 and Figs. 2a and 2b). Immunoblot analysis also revealed that VDR loss in HCT116 or RKO cells led to decreased expression of a select group of the above proteins whereas overexpression of VDR had the reverse effect (Figs. 2c and S2a). Although VDR overexpression in p53^+/+^ HCT116 and MEFs increased peroxisomal FAO protein expression, the levels of six of these proteins were markedly attenuated in p53^−/−^ cells, even in the presence of ectopically-expressed VDR (Figs. 2d and S2b). Interestingly, a number of these genes contained VDR response elements (VDREs).

To determine whether VDREs regulate peroxisomal FAO genes expression, we cloned WT or mutant VDREs into the promoter region of a firefly luciferase reporter plasmid and observed only the former to be active (Fig. 2e). Luciferase reporters containing peroxisomal FAO gene promoter elements with WT VDREs but not with mutant VDREs were also dramatically up-regulated when treated with Nutlin3, suggesting that the regulation of peroxisomal FAO genes expression by p53 is also VDR dependent (Fig. 2e). Consistent with this, ChIP analysis showed more pronounced binding of VDR to the promoters of peroxisomal FAO pathway genes in p53^+/+^ cells versus p53^−/−^ cells (Figs. 2f and S2c).

Given the apparent importance of the VDR in promoting the p53 response of peroxisomal FAO genes, we next explored its mechanism of action. Confirming previous findings that VDR is a direct transcriptional target of p53 [23], Nutlin3-treated HCT116 cells, but not p53^−/−^ HCT116 cell, significantly increased VDR mRNA and protein expression (Figs. S2d and S2e). Employing co-immunoprecipitation (Co-IP) experiments, we confirmed previous reports that both WT and mutant p53 interact with the VDR [24] and also found this association to be mediated through the p53 C-terminal regulatory domain (CTD) and the VDR activation function-2 (AF2) domain (Fig. S2f-h). Cell fractionation further demonstrated that p53 reactivation by Nutlin3 caused substantial nuclear translocation of VDR in some cells (Fig. S2i). Collectively, these results strongly suggest that p53 directly interacts with the VDR to enhance peroxisomal FAO-related gene transcription.

### Acetyl-CoA from p53-mediated peroxisomal FAO regulates ATIC activity

**Fig. 3:**
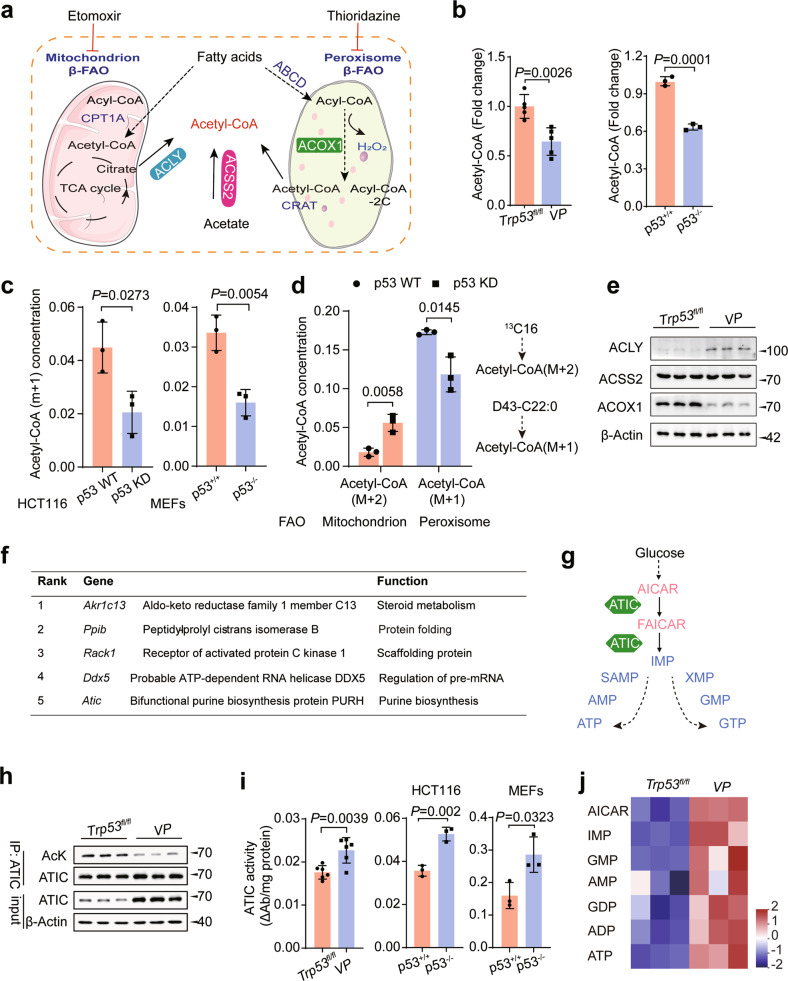
Acetyl-CoA from p53-mediated peroxisomal FAO regulates ATIC activity. **a** A schematic diagram of the mitochondrial and peroxisomal FAO in mammalian cells and cytosolic acetyl-coA production. ACLY, ATP-citrate lyase; ACSS2, Acyl-CoA synthetase short-chain family members 2; ACOX1, Acyl-CoA oxidase 1. **b** Cytosolic acetyl-CoA measurement in the tumor tissues from CRC mouse model and HCT116 cells, *n* = 3 or 5. **c** Docosanoic-d43 acid isotope profiling analysis revealed acetyl-CoA (*m* + 1) metabolism is suppressed when p53 deletion in HCT116 and MEFs, *n* = 3. **d** Docosanoic-d43 acid and palmitate (^13^C16) isotope profiling analysis was performed in the HCT116 cells, *n* = 3. **e** Western blot analysis of ACLY, ACSS2, and ACOX1 expression in n CRC tumors from *Trp53*^*fl/fl*^ and *VP* mice, *n* = 3. **f** Top acetylation of metabolic enzymes that are significantly associated with p53 KO. **g** A schematic diagram of the purine biosynthesis pathway. **h** Lysine acetylation of endogenous ATIC was analyzed in CRC tumors from *Trp53*^*fl/fl*^ and *VP* mice by immunoprecipitation using an ATIC antibody, followed by western blot analysis using AcK antibody. **i** Effect of p53 deletion on ATIC activity in CRC tumors from CRC mouse model, HCT116 and MEFs, *n* = 3 or 6. **j** Relative purine nucleotide levels were analyzed by LC-MS/MS in CRC tumors from *Trp53*^*fl/fl*^ and *VP* mice and normalized to per mg tissue, *n* = 3. Data were presented as mean ± SD. ns, non-significant, *P* > 0.05.

Peroxisomes play an essential role in lipid metabolism, with each round of FAO producing one mole of a shorter fatty acyl-CoA (-2C), one mole of H_2_O_2_, and one mole of acetyl-CoA [5] (Fig. 3a). In this context, we found the total cytosolic acetyl-CoA levels in AOM/DSS CRCs from *VP* mice tissues and p53^−/−^ cells to be significantly lower than in their WT counterparts (Fig. 3b). Moreover, in a [^2^H]-docosanoic acid isotopic tracer experiment, the amount of labeled acetyl-CoA (m + 1) derived from peroxisomal FAO was significantly reduced in p53-deficient cells (Fig. 3c) and no detectable differences in H_2_O_2_ levels were seen (Fig. S3a). Previous reports indicated that p53 promotes mitochondrial FAO under metabolic stress [2, 25], which may contribute to the increase of cytosolic acetyl-CoA levels. However, [^13^C]-palmitic acid labeling experiment revealed that the cytosolic acetyl-CoA (M + 2) levels in p53 KD cells to be significantly higher than in p53 WT cells (Fig. 3d). Cytosolic acetyl-CoA is generated primarily from citrate by the lipogenic enzyme ACLY. Secondly, acyl-CoA synthetase short-chain family, member 2 (ACSS2), employs acetate to produce acetyl-CoA in an ATP-dependent manner (Fig. 3a) [9]. Remarkably, the expression of ACLY markedly increased in *VP* mice, while ACOX1 expression decreased (Fig. 3e). Correspondingly, activation p53 significantly decreased ACLY expression, while ACOX1 increased in CRC cells (Fig. S3b). There was no significant change in ACSS2 expression observed in vitro and in vivo, regardless of treatment (Figs. 3e and S3b). Thus, these results demonstrate the important role of peroxisomal FAO in cytosolic acetyl-CoA production in CRC cells and MEFs with wild-type p53.

Beyond its role in energy metabolism and biosynthesis, acetyl-CoA also functions as the acetyl group donor for many proteins [9]. To identify those whose acetylation was regulated by p53, we use MS to analyze all acetylated peptides from the CRCs of *Trp53*^*fl/fl*^ and *VP* mice (Extended Data Table 4). The latter showed a relative paucity of acetylated proteins functioning in glycolysis, the pentose phosphate pathway, fructose and mannose metabolism, the biosynthesis of amino acids, and purine metabolism. The most prominent of these differences were in AKR1C13, PPIB, RACK1, DDX5, and ATIC (Fig. 3f). Because GSEA of our RNA-seq dataset in *VP* tissues had shown selective enrichment of transcripts involved in the nucleotide biosynthesis and ATIC is the only one of these proteins involved in purine metabolism, we focused on this protein (Fig. S3c).

ATIC is a bifunctional enzyme that catalyzes the last two steps of *de novo* purine biosynthesis (Fig. 3g) [19]. We, therefore, investigated the role of ATIC in the p53 tumor suppressor network and confirmed that CRCs from *VP* mice had lower levels of acetylated ATIC (Fig. 3h). Both the activation of p53 by Nutlin3 and the ectopic expression of WT p53 in CRC cells or MEFs dramatically increased the acetylation of both endogenous and exogenous ATIC whereas depleting p53 had the opposite effect (Figs. S3d and S3e). Furthermore, ACOX1 inhibition also led to a decrease in acetyl-CoA levels and ATIC acetylation, which could be reversed by re-expressing ACOX1, while acetylation of histone H3 was unaffected (Fig. S3f).

The acetylation of metabolic enzymes plays important role in regulating their activities [26]. Given that p53 enhanced ATIC acetylation, we next investigated whether this affected ATIC activity. We first found that p53 deletion in WT cells markedly increased ATIC activity whereas p53 activation had the opposite effect (Figs. 3i and S3g). Moreover, p53 knockout also resulted in significantly enhanced purine synthesis in *VP* mice with CRCs (Fig. 3j). Next, we used MS to identify three acetylated lysine residues (K266, K356, and K524) in ATIC (Extended Data Table 6). We mutated each of these to arginine (R) and found the K266R mutant to be associated with both significantly reduced ATIC acetylation and increased enzymatic activity (Fig. S3h). This indicated that K266 is an acetylation site that negatively regulates ATIC’s activity. This was supported by the finding that K266 is highly conserved across species (Fig. S3i). The K266R mutant also demonstrated higher ATIC activity that was no longer responsive to Nutlin3 and thereby indicating that p53 inhibits ATIC activity by promoting K266 acetylation (Fig. S3j). Collectively, these data show that acetyl-CoA derived from p53-mediated peroxisomal FAO inhibits ATIC activity by promoting ATIC acetylation.

### p53 increases ATIC acetylation and promotes ATIC degradation by transcriptionally activating KAT2B

**Fig. 4:**
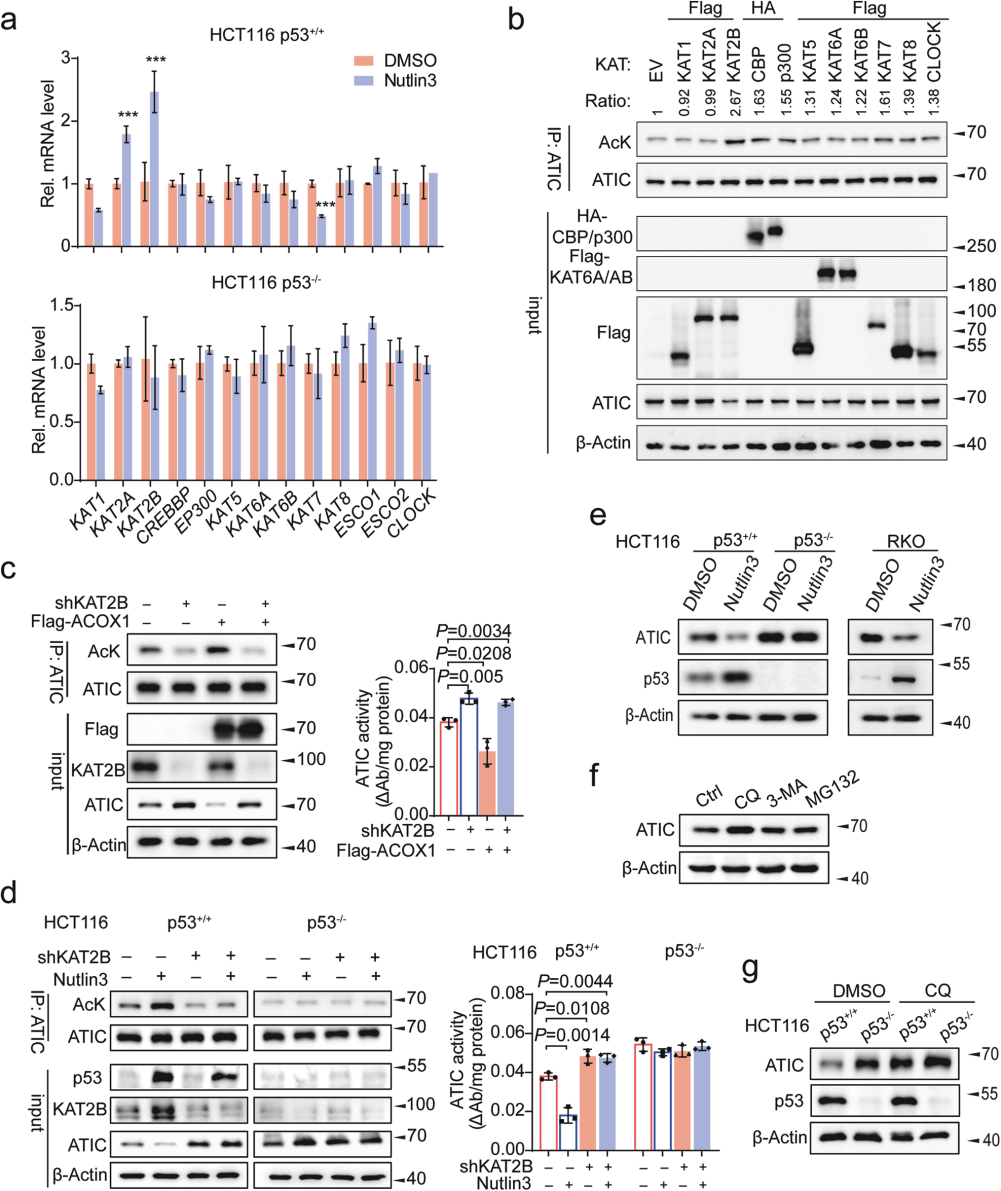
p53 increases ATIC acetylation to trigger ATIC degradation by transcriptionally activating KAT2B. **a** HCT116 p53^+/+^ and p53^−/−^ cells were treated with either DMSO or Nutlin3 for 48 h, and the expression of 13 KAT family acetyltransferases genes were assessed by qRT-PCR, *n* = 3. **b** Western blot analysis of ATIC acetylation in HCT116 cells with overexpression of KATs. Signal intensity of Ac-ATIC protein was quantified by Image J, the acetylation level of ATIC as indicated was normalized against immunoprecipitated ATIC. **c** HCT116 cells expressing shCtrl or KAT2B shRNA further infected with control or Flag-ACOX1 vector, followed by western blot analysis of ATIC acetylation (left) and enzyme activity (right), *n* = 3. **d** HCT116 cells expressing shCtrl or KAT2B shRNA further treated with or without Nutlin3 for 24 h, followed by western blot analysis of ATIC acetylation (left) and enzyme activity (right), *n* = 3. **e** Protein expression of ATIC in CRC cells treated with or without Nutlin3 for 48 h. **f** ATIC immunoblots of lysates from HCT116 cells treated with CQ (50 μM), 3-MA (10 mM), or MG132 (10 μM) for 10 h before harvesting. **g** HCT116 p53^+/+^ and p53^−/−^ cells treated with or without CQ (50 μM) for 10 h, followed by western blot analysis of ATIC expression. Data were presented as mean ± SD. **P < 0.01, ***P < 0.001.

To identify the acetyltransferase(s) responsible for ATIC K266 acetylation, we examined the expression of 13 KAT family member acetyltransferases following Nutlin3 treatment and found only KAT2B expression to be significantly increased in both HCT116 and HIEC cells (Figs. 4a, S4a, and S4b). We also overexpressed several classical acetyltransferases and found that only KAT2B significantly increased ATIC acetylation (Figs. 4b and S4c). Furthermore, KAT2B proved capable of acetylating WT-ATIC and all its mutants except K266R (Fig. S4d). Conversely, depleting KAT2B significantly decreased ATIC acetylation in a manner that could be rescued by KAT2B re-expression (Fig. S4e). An endogenous interaction between KAT2B and ATIC was also documented in CRC cells by Co-IP experiments (Fig. S4f). We also found that inhibiting KAT2B significantly reduced ATIC acetylation and increased its activity in a manner that could not be reversed by Nutlin3 or ACOX1 overexpression (Figs. 4c and 4d). Taken together, these findings show ATIC K266 to be a specific site of KAT2B-mediated acetylation that is mandatory to suppress ATIC activity and that the *KAT2B* gene is itself a direct transcriptional target of p53.

To gain mechanistic insight into p53-mediated inhibition of ATIC activity, we found that p53^−/−^ HCT116 cells express more ATIC protein than their p53^+/+^ counterparts and that Nutlin3 decreased ATIC protein without affecting its mRNA levels (Figs. 4e and S4g). Chloroquine (CQ), a lysosome inhibitor, could block ATIC degradation (Fig. 4f). Furthermore, ATIC’s half-life was significantly shortened when treated with Nutlin3 (Fig. S4h). Consistent with these findings, ATIC-K266Q and ATIC-K266R had markedly shorter and longer, half-lives, respectively (Fig. S4i). Moreover, a significant difference was not observed at protein level of ATIC when treated with CQ in HCT116 p53^+/+^ and HCT116 p53^−/−^ cells (Fig. 4g) and degradation through ATIC acetylation by p53 was interdicted (Figs. 4g and S4j). Thus, these results suggest that p53 promotes ATIC degradation through acetylation by KAT2B, which consequently suppresses ATIC activity.

### Acetylation of ATIC is correlated to colorectal tumorigenesis

**Fig. 5:**
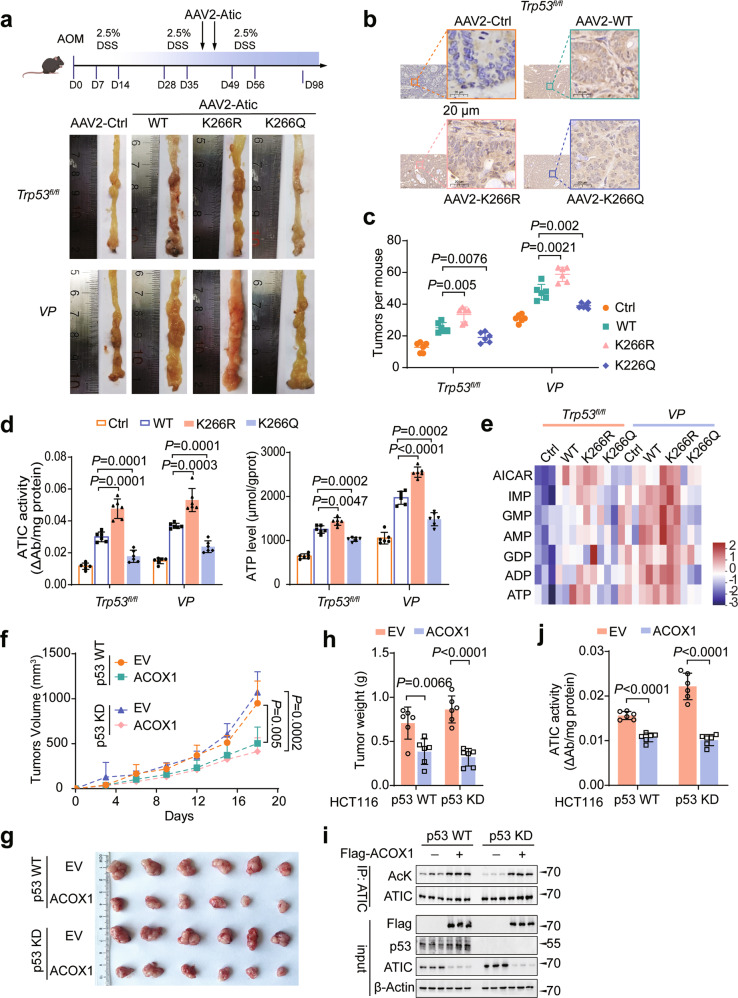
Acetylation of ATIC is closely correlated to colorectal tumorigenesis. **a** A Scheme for the AOM/DSS-induced colon cancer model in *Trp53*^*fl/fl*^ and *VP* mice (top). Each mouse received two intraperitoneal injections of the indicated virus during the second processing of DSS treatment. Typical images of colon tumors expressing the indicated ATIC proteins 98 days after AOM/DSS treatment. **b** ATIC immunohistochemistry (IHC) in representative sections of tumor tissues from **a**. Scale bar, 20 μm. **c** Colon tumor numbers in mice from **a**, *n* = 6. **d** ATIC activity and ATP levels were measured in tumor tissues from **a**, *n* = 6. **e** Relative purine nucleotide levels were analyzed by LC-MS/MS in CRC tumors from *Trp53*^*fl/fl*^ and *VP* mice and normalized to per mg tumor tissue, *n* = 3. **f**–**h** HCT116 cells with stable expression the indicated vectors were injected subcutaneously into BALB/c nude mice, *n* = 6. Tumor size was measured every 3 days **f**. Tumors were dissected and photographed (**g**) and weighed on day 18 (**h**). **i** Western blot analysis of ATIC acetylation in tumor tissues from each group, as in *f*, *n* = 3. **j** ATIC activity was measured in tumor tissues from **f**, *n* = 6. Data were presented as mean ± SD.

We next explored the effect of ATIC K266 acetylation on tumor growth. To this end, CRCs were induced in *Trp53*^*fl/fl*^ or *VP* mice by AOM/DSS, followed by intraperitoneal injection with adeno-associated vectors (AAV) encoding WT-ATIC, ATIC-K266R, ATIC-K266Q or a control empty vector and ATIC expression was confirmed by immunohistochemistry (IHC) and western blot (Figs. 5a, 5b, S5a and S5b). Ectopic expression of ATIC-K266R promoted more rapid tumor growth compared with that of WT-ATIC in both *Trp53*^*fl/fl*^ and *VP* mice (Figs. 5a and 5c). We found that *Trp53* KO mice showed significantly enhanced tumor growth; even so, delivery of ATIC-K266Q inhibited tumor growth compared with WT-ATIC even in *VP* mice (Figs. 5a and 5c). Human CRC cells expressing ATIC-K266R also proliferated faster than those expressing WT-ATIC (Fig. S5c). However, K266Q mutant still significantly inhibited cell proliferation even in p53-deficient CRC cells (Fig. S5c). Interestingly, when p53 was stably expressed in HCT116 cells, the WT-ATIC exhibited inhibiting effect like K266Q mutant on cell proliferation (Fig. S5d). However, the K266R mutant still strongly accelerated cell proliferation (Fig. S5d), indicating that p53-induced highly acetylated ATIC had a similar effect of the mimicked K266Q mutant on suppressing tumor growth and cell proliferation. Furthermore, the K266Q mutant led to a greatly reduction of ATIC enzyme activity and purine nucleotide levels (IMP, AMP, and GMP), while the K266R mutant showed the opposite effects compared with those with K266Q mutant (Figs. 5d, 5e, S5e and S5f). Interestingly, exogenous expression of ATIC-K266Q partially promote tumor growth compared with AAV-Ctrl (Figs. 5a, 5c and S5c). This implies that other post‐translational modifications may also play a role in regulating ATIC activity.

Taken together, these results demonstrate that ATIC acetylation plays an important role in the regulation of CRC growth.

Having shown the importance of ATIC acetylation inhibits CRC growth, we next explored the role of ACOX1 in this process. Overexpression of ACOX1 significantly inhibited tumor growth and tumor weights in both p53 WT and KD cells (Fig. 5f–h). Western blot analysis confirmed that ATIC acetylation significantly increased when overexpression of ACOX1 (Fig. 5i). Meanwhile, ACOX1 overexpression also effectively inhibited ATIC activity in mouse xenograft tumor tissues (Fig. 5j). Together, these findings suggest that ACOX1-mediated ATIC acetylation is critical for tumor suppression.

### Targeting ATIC suppresses colorectal tumorigenesis

**Fig. 6:**
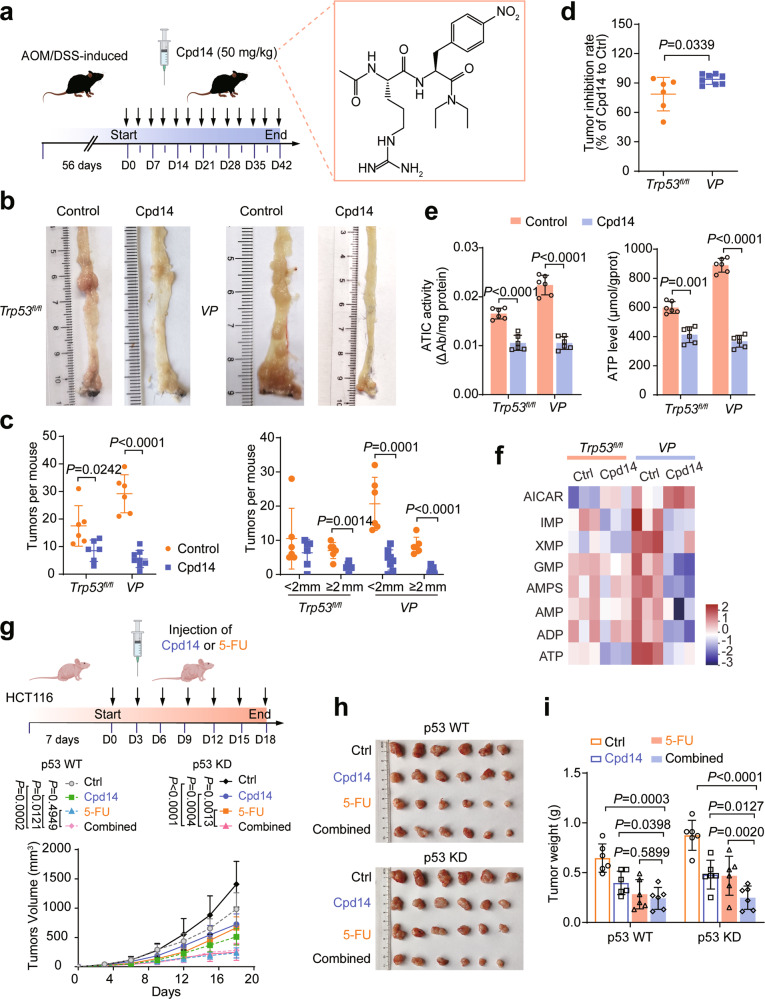
Targeting ATIC suppresses colorectal tumorigenesis. **a** A scheme of Cpd14 treatment during AOM/DSS-induced *Trp53*^*fl/fl*^ and *VP* mouse CRC model (left). The chemical structure of Cpd14 (right). **b** Typical images of colon tumors from Control and Cpd14-treated mice 42 days after Cpd14 treatment. **c** Colon tumor numbers in mice from **a**, *n* = 6 or 8. **d** Tumor inhibition rate for Cpd14-treated mice relative to control mice in p53 WT and p53-deficient mice, *n* = 6 or 8. **e** ATIC activity (left) and ATP levels (right) in tumor tissues from AOM/DSS-induced CRC mouse model, *n* = 6. **f** Purine metabolite abundance were determined by LC-MS/MS in AOM/DSS-induced CRC mouse treated with Cpd14 or not, *n* = 3. **g**–**i** Schematic depicting experimental setup. Nude mice bearing HCT116 tumors were intraperitoneally administered 5-Fluorouracil (5-FU) and Cpd14, either alone or in combination, *n* = 6. Tumor size was measured every 3 days (**g**). Tumors were dissected and photographed (**h**) and weighed on day 18 (**i**). Data were presented as mean ± SD.

Cpd14 is a small-molecule inhibitor that prevents the homodimerization and activation of ATIC (Fig. 6a) and thereby inhibits the proliferation of MCF-7 breast cancer cells and HCT116 cells [27, 28]. We found that Cpd14 also dramatically suppressed tumor growth rates and total tumor burden in AOM/DSS-induced CRC and *Apc*^*Min/+*^ mice. This was more pronounced *VP* and *AP* mice than in control mice (Figs. 6b–d and S6b-d), indicating that p53-deficient tumors were more sensitive to ATIC inhibition. Cpd14 treatment also more significantly reduced ATIC activity and ATP levels in *VP* and *AP* mice (Figs. 6e and S6e). Of note, Cpd14 treatment resulted changes in purine nucleotide pools that included the accumulation of AICAR and the depletion of IMP, XMP, AMP, and ATP, all of which were consistent with the intended inhibition of ATIC (Fig. 6f).

We also evaluated the potential synergy between Cpd14 and 5-Fluorouracil (5-FU) on cell-derived xenografts (CDX) in BALB/c nude mice (Fig. 6g). Treatment with either compound alone was effective in reducing the growth of both p53 WT and p53 KD cells. The combination of Cpd14 and 5-FU provided no additional benefit over 5-FU alone in p53 WT cells but did prove effective in p53 KD cells suggesting that p53 knockdown tumor cells were more sensitive to combination treatment (Fig. 6g–i).

To further verify the above results, we next determined whether inhibiting ATIC by AAV2-Atic shRNA delivery could delay CRC. This experiment was designed as illustrated in Fig. S6f and immunohistochemistry (IHC) and western blot analysis confirmed the KD of Atic (Figs. S6g and S6h). Essentially identical to the degree of CRC growth inhibition obtained with Cpd14 treatment, Atic KD diminished tumor burden by ~85% in *VP* mice and by ~65% in *Trp53*^*fl/fl*^ mice (Figs. S6f, S6i and S6j). The effects on ATIC activity and ATP levels were also more obvious in *VP* mice (Fig. S6k). In vitro studies also revealed directly that ATIC KD significantly repressed the proliferation of human CRC cells which reduced ATIC enzyme activity and ATP levels (Figs. S6l and S6m) with the effects again being more pronounced in p53^−/−^ cells.

### ATIC acetylation is downregulated in human CRCs

**Fig. 7:**
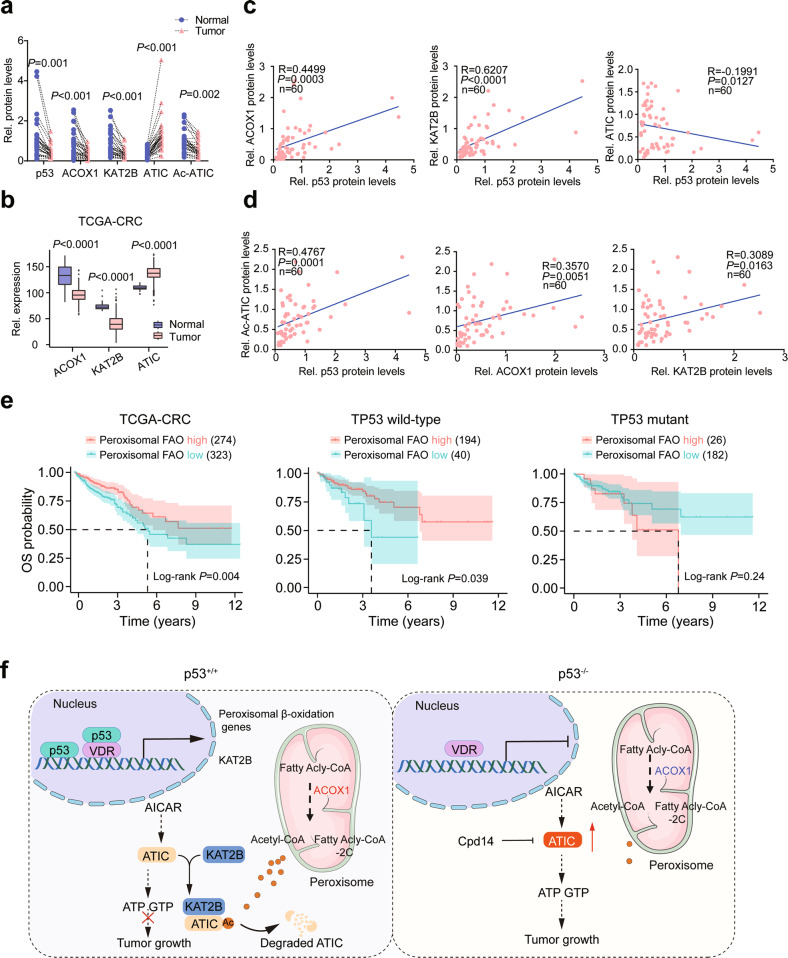
Acetylation of ATIC is downregulated in human CRCs. **a** Relative protein levels of p53, ACOX1, KAT2B, ATIC. The results shown are from the same tissues shown in Fig. S7a. Signal intensity of these proteins were quantified by Image J, and then normalized to β-actin band intensity. **b** *ACOX1*, *KAT2B*, *ATIC* expression in CRC tumors and normal colorectal tissues. The raw data from TCGA. **c** Correlation between the expression levels of p53 and ACOX1, KAT2B, ATIC, as determined by Pearson’s *r* analysis. The results shown are from the same tissues shown in Fig. S7a. **d** Correlation between the expression levels of Ac-ATIC and p53, ACOX1, KAT2B, as determined by Pearson’s *r* analysis. The results shown are from the same tissues shown in Fig. S7a. **e** Kaplan–Meier analysis of overall survival (OS) in CRC patients according to the expression of peroxisomal FAO. *P* values were calculated by log-rank test. The raw data was from TCGA. **f** Schematic illustration of the mechanistic model for the role of p53-mediated peroxisomal β-oxidation-ATIC axis in colorectal tumorigenesis. Data were presented as mean ± SD.

To further study the potential clinical relevance of the above-described novel p53-KAT2B-ATIC axis, we collected 45 pairs of human CRC samples (T) with adjacent normal colon tissues (N) along with detailed pathological and clinical information (Extended Data Table 8). Among these samples, 30 cases of p53 WT samples for analysis. In addition, p53, ACOX1 and KAT2B proteins were significantly decreased in CRC samples relative to control adjacent normal colonic tissue (Figs. 7a and S7a), which is consistent with the data from TCGA, GEO and Clinical Proteomic Tumor Analysis Consortium (CPTAC) database (Figs. 7b, S7b and S7c). In general, ACOX1 and KAT2B protein levels were positively correlated with p53, whereas ATIC levels were negatively correlated (Fig. 7c). ATIC acetylation levels also positively correlated with p53, ACOX1, and KAT2B (Fig. 7d) and significantly decreased in CRC samples (Fig. 7a). We further explored the association between these genes’ expression and patient prognosis in human carcinomas by using data from TCGA database. Interestingly, we found that patients with high-level expression of peroxisomal FAO genes had favorable survival (Fig. 7e). Importantly, this association was only observed in *TP53* wild-type patients, but not *TP53*-mutant patients, status (Fig. 7e). These data indicate that, as in the case of the murine AOM/DSS and *APC*^*min/+*^ CRC model, ATIC acetylation plays a critical role in human CRC pathogenesis.

## Discussion

p53 has increasingly been shown to control many metabolic processes that limit tumor growth [3]. We have shown here that this includes the activation of a suite of genes encoding peroxisomal enzymes that promote FAO. As a result, acetyl-CoA, the critical downstream product of this pathway, accumulates in cytoplasm where it serves as a substrate for KAT2B-mediated ATIC acetylation. These observations provide a direct functional link between p53-regulated peroxisomal FAO and purine biosynthesis that mediates tumor suppression (Fig. 7f).

Although p53 regulates lipid metabolism mainly through transcriptional processes [2], we found here that it also facilitates peroxisomal FAO at the protein level. Previous work has shown that p53 regulates lipid metabolism by protein–protein interaction and that it inhibits the pentose phosphate pathway (PPP) by binding to glucose-6-phosphate dehydrogenase (G6PD) and preventing its dimerization-mediated activation [29]. In addition, in hepatocyte-specific *Sirt1* knockout mice fed high-fat diets, there is a decrease in FAO [30]. *SIRT1* mRNA expression is induced by a complex formed between p53 and the forkhead transcription factor Foxo3a [30]. Analogously, our results demonstrate that p53 can also cooperate with the VDR to enhance peroxisomal FAO-related gene transcription.

A recent study indicates that overexpression of VDR, which is a direct transcriptional target of p53 in some cell types [23], can suppress the cancer stem cell phenotype of CRC cells and decrease invasion [31]. VDR signaling pathways may partially overlap with those of p53 by virtue of sharing several common direct target genes [23]. Indeed, we observed that the expression of peroxisomal FAO rate-limiting enzymes correlates with VDR expression in WT p53 cells but not in HCT116 p53^−/−^ cells (Figs. 2d and S2b). Beyond the induction of VDR mRNA expression, p53 also appears to be involved in determining the amount of nuclear-localized VDR (Fig. S2i).

A decrease in peroxisome activity has been reported in CRCs and breast, liver, and kidney cancers [32]. In addition to producing H_2_O_2_. Peroxisomes are key metabolic organelles, which contribute to cellular lipid metabolism at various levels that include lipid and bile acid synthesis, α-oxidation of branched-chain fatty acids, and β-oxidation of VLCFAs and the maintenance of redox balance [33]. In rodents, abnormal upregulation of ACOX1 by PPAR activation stimulates hepatic FAO and the accumulation of H_2_O_2_, leading to excess energy burning in the liver and contributing to the development of liver cancer [6, 34]. This is possibly caused by sustained PPARα activation in hepatocytes [35]. While H_2_O_2_ levels were similar in *VP* CRC tissues and HCT116 p53^−/−^ cells compared with the control group (Fig. S3a). p53 also activates several antioxidant proteins, including peroxiredoxins, catalase, and glutathione peroxidase 1 (GPX1), which may reduce H_2_O_2_ levels [6]. We measured catalase and GPX1 enzyme activity in a variety of cell lines and CRC tumors from *Ttp53*^*fl/fl*^ and *VP* mice and found higher levels of these in the former (data not shown).

Mitochondrial acetyl-CoA pools come from pyruvate, fatty acid, amino acid, and ketone body metabolism [36]. However, acetyl-CoA cannot be directly transported across mitochondrial membranes, cytosolic acetyl-CoA is generated primarily from ACLY, ACSS2, and ACOX1 [9, 16]. In our studies, the cytosolic acetyl-CoA (M + 2) levels were higher in p53 KD cells, which is produced from palmitic acid-^13^C16 (Fig. 3d). However, the total cytosolic acetyl-CoA was still decreased in p53-deficient mice and cells (Fig. 3b). p53 has been reported to repress the expression of SREBP1c, and therefore the expression of the SREBP1c target gene *ACLY* [37]. Consistent with this, ACLY protein levels were dramatically increased in p53-deficient mice and cells (Figs. 3e and S3c). p53 inhibits the expression of ACLY, which hinders the contribution of mitochondrial FAO to cytosolic acetyl-CoA. The mitochondrial acetyl-CoA, then enters the TCA cycle, where it is further oxidized to CO_2_ with the concomitant generation of ATP. It is not surprising because p53 promotes mitochondrial FAO by increasing the expression of several key enzymes (i.e., CPT1C, Acad11, LPIN1), which may important for p53 promotes oxidative phosphorylation (OXPHOS) to maintain energy and cell survival under metabolic stress [11–13]. In this context, p53 may provide a survival advantage, while this advantage may be short-lived. Because tumor cell growth requires *de novo* synthesis of fatty acids, p53 inhibits fatty acid synthesis and promotes FAO [38], the long-term imbalance between fatty acid synthesis and oxidation is not conducive to tumor growth. In addition, this effect highly depends on stress conditions and collaborating environmental signals. Although peroxisome FAO is a major source of cytosolic acetyl-CoA in p53 WT cells, there also likely exists cooperation and feedback between peroxisomes and mitochondria. Nonetheless, our results indicate that the important role of peroxisomal FAO in acetyl-CoA in p53 WT cells.

Acetyl-CoA is also the source of acetyl for histone acetylation [36]. In plants, peroxisomal FAO regulates histone acetylation [39]. However, He et al found that acetylation of histone H3 was unaffected by Acox1 knockout [16]. Consistent with this report, neither knockdown nor overexpression of ACOX1 has any effect on histone H3 in our study (Fig. S3h), suggesting that peroxisome-derived acetyl-CoA might have a selective role in acetylation. Furthermore, peroxisomal branched-chain fatty acid metabolism might be associated with a broader variety of tumors [40]. For example, the expression of some enzymes involved in peroxisomal branched-chain fatty acid degradation is increased in prostate tumors [40, 41]. Our current view on the role of peroxisomes in cancer cells remains fragmentary and in some cases contradictory. These discrepancies may be due to the heterogeneity of tumors analyzed and may require further correlations with such factors as tumor subtype, gene expression and mutational profiles, and/or epigenetic status.

ATIC activation is dictated not only by acetyl-CoA, whose levels are determined by p53’s control over peroxisomal FAO, but also by KAT2B. Although *KAT2B* is a p53 target gene [42], its physiological function and role in tumor suppression have until now been poorly understood. Previous studies showed KAT2B expression to be downregulated in esophageal squamous cell and hepatocellular carcinomas, and in the latter case, to be associated with inferior overall survival [43]. We observed that p53 promotes *KAT2B* transcription and that its expression was positively correlated with p53 in CRC samples. Moreover, in human CRC, data from UALCAN indicate that *KAT2B* expression is higher in *TP53* WT than in *TP53*-mutant samples (data not shown). Our work indicates that *KAT2B*, a p53 target gene, is not only critical for colorectal tumorigenesis but that its central role lies with its ability to acetylate ATIC and thus indirectly regulate purine levels.

Our data demonstrated that pharmacological and genetic inhibition of ATIC, inhibits purine synthesis and tumor growth both in vivo and in vitro. It is worth noting that the inhibition may partially rely on p53-mediated peroxisomal FAO. To support rapid proliferation, cancer cell enhances their rates of nucleotide synthesis, which is in turn reduced in the face of p53-mediated cell cycle arrest. p53 also limits nucleotide biogenesis by directly inhibiting inosine monophosphate dehydrogenase (IMPDH) and guanine monophosphate synthase (GMPS), which are involved in *de novo* guanosine triphosphate biosynthesis [44–46]. p53 further inhibits *de novo* purine synthesis by transcriptionally inhibiting *MTHFD2*, which encodes a key enzyme in folate metabolism [47]. Thus, this suggests that other mechanisms are equally important for p53-mediated limiting nucleotide biogenesis and tumor growth.

Pharmacological inhibition of purine synthesis has been studied as a potential cancer therapy [48]. For example, Huang et al. found that ASCL1^Low^ small cell lung cancers with high levels of MYC are sensitive to mycophenolic acid (MPA), an IMPDH inhibitor. Some antifolates, such as lometrexol, were also designed to target ATIC activity and inhibit *de novo* purine synthesis [49]. However, many of these therapies have adverse toxicities. In contrast to these agents, which directly inhibit ATIC’s enzymatic activity, Cpd14 is a peptide-based ATIC inhibitor that disrupts the enzyme’s homodimerization and thus indirectly impairs its activity and the proliferation of certain cancer cell types [27, 28]. Notably, ATIC inhibition by such approaches is more effective in p53-deficient cells than in p53 WT cells. Moreover, inhibition of ATIC not only impairs *de novo* purine biosynthesis but also leads to a large accumulation of AICAR [50], which potently activates AMPK [51] and inhibits the growth of some glioblastomas by repressing adipogenesis [52].

Collectively, these studies identify a previously unrecognized p53-peroxisomal FAO-purine biosynthesis axis that permits cross-talk between these pathways and regulates growth so as to ensure a proper balance between proliferation and the supply of purine nucleotides. At least for CRC, p53 facilitates the acetylation-dependent deactivation of ATIC, which plays a key role in p53 tumor suppressive activities. Ultimately, deconstructing this pathway through which p53 acts may lead to new opportunities for therapeutic intervention in cancer.

## Methods

### Plasmids and constructs

The open reading frames of human *TP53*, *VDR*, *ACOX1*, *ATIC*, and *KAT2B* or mouse *Atic* were amplified and cloned into pHAGE-CMV-Flag, pCMV-HA, pLKO-GFP or pAAV-EF1a-GFP. KATs were kindly provided by Prof. Hong-Bing Shu (Wuhan University, China) [53]. Mutations in the ATIC cDNA sequences were generated by overlap extension PCR. Human *TP53*, *VDR*, *ACOX1*, *ATIC,* and *KAT2B* or mouse Atic shRNAs were synthesized by from GENECREATE (Wuhan, China), subsequently annealed, and inserted into the pLKO.1-puro vector. Human VDR sgRNAs were ordered from Lentiviral CRISPR gRNA Sanger Library Human (Sigma). Primers, shRNAs, and sgRNAs used in this study are listed in Extended Data Table 7.

### Human CRC samples

CRC samples were collected between September 2018 and December 2020 at the Zhongnan Hospital of Wuhan University. All samples were collected from patients after obtaining informed consent in accordance with a protocol approved by the Ethics Committee of Zhongnan Hospital of Wuhan University. We collected samples from 45 patients with CRC with detailed pathological and clinical information. Among the 45 samples, 30 cases of p53 WT samples were for analysis. Detailed clinical information for these patients is presented in Extended Data Table 8.

### Cell lines and cell culture

Human embryonic kidney HEK293T cells, human colon cancer cell lines, RKO, HCT116, HCT116 (p53^−/−^), and human normal intestinal epithelial HIEC cells were obtained from ATCC. MEFs were obtained by cesarean section of pregnant females from timed mating of *Trp53*^*em1Cd*^*/Gpt* heterozygous mice as previously described [54]. Embryos were genotyped using primers listed in Extended Data Table 7. All cells were regularly authenticated by short tandem repeat analysis, tested for the absence of Mycoplasma contamination, and were cultured in Dulbecco’s modified Eagle’s medium (DMEM, Gibco) supplemented with 10% fetal bovine serum (Gibco) and 1% of penicillin and streptomycin (Life Technologies, 15140) in 5% CO_2_ at 37 °C.

### Mice

All animal procedures were approved by the Animal Care Committee of Wuhan University. *Trp53*^*fl/fl*^ (008462) mice were purchased from Jackson Laboratory and kindly provided by Dr. Bo Zhong (Wuhan University) [55]. The *Pvillin-Cre* (T000142) mice were purchased from Cyagen (Guangzhou, China). *Apc*^*Min/+*^ (T001457) and *Trp53*^*em1Cd*^*/Gpt* (T005332) mice were purchased from GemPharmatech (Jiangsu, China). *Trp53*^*fl/fl*^ mice were crossed with *Pvillin-Cre* mice to generate *Pvillin-Cre* + *Trp53*^*fl/fl*^ (*VP*) mice. *Apc*^*Min/+*^ mice were crossed with *VP* mice to generate *Apc*^*Min/+*^ *Pvillin-Cre* + *Trp53*^*fl/fl*^ (*AP*) mice. Mice were genotyped using the primers listed in Extended Data Table 7. All mice were housed in a specific-pathogen-free (SPF) animal facility at Wuhan University. AOM/DSS-induced CRCs were generated following the previously described procedures [56]. Briefly, 8-week-old male C57B6/J, *Trp53*^*fl/fl*^ and *VP* mice were injected intraperitoneally with 10 mg/kg AOM (Sigma-Aldrich). Seven days later, mice were given drinking water containing 2.5% DSS (MP Biomedicals, Santa Ana, CA, USA) for 7 days followed by 2 weeks regular drinking water for recovery. This same cycle was repeated twice. At day 98, tumor burdens were evaluated.

For administration of adeno-associated virus (AAV) to AOM/DSS-induced CRC mice, male *Trp53*^*fl/fl*^ or *VP* mice were randomly distributed into nine groups. The first of these was treated with AAV2 expressing AAV2-shCtrl and two groups of mice were treated with AAV2 expressing shAtic. Four additional groups of mice were treated with AAV2 expressing the control vector, WT-Atic or the indicated Atic mutants. 1 × 10^11^ AAV2 viral particles were intraperitoneally injected at local multiple sites into each mouse. Two groups of mice were injected i.p. with control agent or Cpd14 (50 mg/kg) [27, 28] twice in a week for 6 weeks. Cpd14 was kindly provided by professor Ali Tavassoli (University of Southampton) [27].

For cell-derived xenograft (CDX) experiments, 6-week-old male BALB/c nude mice (D000521, GemPharmatech, Jiangsu) were injected subcutaneously with HCT116 cells (2 × 10^6^ cells/mice). The animals were randomly divided into eight groups 1-week post-injection; Vehicle, Cpd14, 5-FU, Cpd14 + 5-FU groups. Cpd14 and 5-FU were intraperitoneally injected every three days at the dose of 50 mg/kg and 30 mg/kg [57], respectively. Tumor size was measured every 3 days. Tumor volume (*T*_*V*_) was calculated was calculated as follows: *T*_*V*_ = 0.52 × Length (mm) × Width (mm)^2^.

### Fatty acid oxidation assays

The effect of p53 knockout on peroxisomal FAO was determined by measuring catabolism of stable isotope–labeled docosanoic acid D39-C20:0 to D43-C22:0 or D3-C16:0 to D3-C22:0 via mass spectrometric analysis, as previously described [21]. Briefly, when HCT116, HCT116 (p53^−/−^) cells, MEFs, and MEFs (p53^−/−^) reached 40% confluency, the normal medium was switched to lipid-free FBS-containing medium supplemented with 30 μM docosanoic acid for three days. Fatty acid isolation and mass spectrometry analysis were performed as previously described [58].

### Luciferase reporter, qRT-PCR, and CHIP assays

Human *ACOX1*, *EHHADH*, *ACAA1* and *SCP2* promoter sequences or VDRE binding sites mutant of the gene’s promoter were inserted into pGL3-basic luciferase vector (Promega). Luciferase assays were performed as previously described [11]. Total RNA was isolated by using Trizol (Life Technology, USA), 1 μg total RNA was reverse transcribed into cDNA using a cDNA Synthesis Kit (Invitrogen, Carlsbad, CA). SYBR^®^ Green Premix Pro Taq HS qPCR Kit (AG11702, Accurate Biotechnology) was applied to conduct quantitative real time PCR. *ACTB* was used as an internal reference. ChIP and qPCR quantification were performed as described [59]. Primer sequences are listed in Extended Data Table 7.

### Cell proliferation assay

Cell proliferation was assessed using the CCK-8 assay. In brief, 2 × 10^3^ cells were seeded in 96-well plates and grown for 5 days. Cell numbers were measured daily using a Cell Counting Kit-8 (TopScience, Inc.) according to the vendor’s instructions.

### Immunoprecipitation, Immunohistochemistry, and western blot analysis

Cells were lysed in RIPA buffer with added PMSF and co-IP and immunoblot analysis was performed as described [56]. To detect ATIC acetylation levels, immunoprecipitations were performed using an anti-ATIC antibody. This was followed by immunoblot using pan acetylation antibodies. Mice were euthanized using carbon dioxide inhalation. Tumors were collected, fixed in 4% paraformaldehyde overnight, transferred to 70% ethanol, and subsequently embedded in paraffin. Hematoxylin and eosin (H&E) and immunohistochemistry (IHC) was performed as described [56]. ATIC antibody (Abcam, ab188321) was used for IHC.

### Subcellular fractionation

Subcellular fractionation analysis was performed using the Nuclear/Cytosol Fractionation Kit (Beyotime, P0027) according to manufacturer’s instructions. For Acetyl-CoA measurement, non-denatured cytosolic fractions were used.

### Mass spectrometry analysis

Protein lysates for LC-MS/MS were extracted from cells as previously described [60, 61]. Briefly, the AOM/DSS-induced CRC model, *Trp53*^*fl/fl*^ and *VP* mice’s tumors were lysed as above. Protein digestion and the affinity-purified anti-acetyl lysine antibody (Cell Signaling Technology). The composition of protein was analyzed by mass spectrometry according to the protocols described previously [62].

### Cycloheximide (CHX) assay

Cells were seeded in six-well plates and allowed to achieve 80% confluency. They were then treated with 100 μg/ml CHX for the indicated times. Cells were collected. lyzed and analyzed by western blot as described [59].

### RNA sequencing (RNA-seq)

Colons were flushed with PBS to clear feces and opened longitudinally. Tumors were dissected away from the surrounding normal tissue and homogenized in 1 ml of TRIzol (Invitrogen). Total RNA was then extracted according to the supplier’s directions and genomic DNA was removed using DNase I (TaKara). RNA quality was determined by 2100 Bioanalyser (Agilent) and quantified with an ND-2000 (NanoDrop Technologies). Only RNA sample with RIN values >8 were used to construct sequencing libraries.

RNA-seq transcriptome libraries were prepared with a TruSeqTM RNA sample preparation Kit from Illumina (San Diego, CA) using 1 μg of total RNA. RNAs were first isolated by poly(A) selection with oligo(dT) beads and then fragmented. Double-stranded cDNA was synthesized using a SuperScript double-stranded cDNA synthesis kit (Invitrogen, CA) with random hexamer primers (Illumina). The newly synthesized cDNA was subjected to end-repair, phosphorylation, and ‘A’ base addition according to Illumina’s library construction protocol. Libraries were size-selected for cDNA target fragments of 300 bp on 2% Low Range Ultra Agarose followed by PCR amplified using Phusion DNA polymerase (NEB) for 15 PCR cycles. After quantified by TBS380, paired-end RNA-seq sequencing library was sequenced with the Illumina HiSeq xten/NovaSeq 6000 sequencer (2 × 150 bp read length).

### Metabolic assays

Acetyl-CoA (MAK039, Sigma) and H_2_O_2_ (BC3595, Solarbio) were measured according to the instructions provided by the manufacturers. AICAR Tfase activity assays were performed as described [63, 64]. Cells were homogenized in 20 mM HEPES-KOH buffer, pH 7.5, 10 mM KCl, 1.5 mM MgCl_2_, 1 mM sodium EDTA buffer, 1 mM sodium EGTA buffer, and 1 mM dithiothreitol in the presence of 250 mM sucrose and protease inhibitor cocktail (Roche Diagnostics). Each reaction mixture contained a final concentration of 66 mM Tris-Cl, pH 7.4, 100 mM 10-f-FH4, 50 mM AICAR, and 50 mM KCl. AICAR TFase was assayed using AICAR and 10-f-H2F by following the production of H2F at 298 nm.

### Acetyl-CoA quantification and stable isotope labeling

Extraction of cytosolic acyl-CoAs from cells was carried out as previously described with some modifications [65, 66]. For isotopic tracer analysis, HCT116 (WT), HCT116 (KD) cells, MEFs (p53+/+), and MEFs (p53−/−) reached 40% confluency, then the cells were changed to lipid-free FBS-containing medium supplemented with 30 μM docosanoic acid (D43-C22:0) or palmitate (^13^C16). After 72 h, acetyl-CoA was extracted as described above for LC-MS analysis.

### Purine nucleotide measurement by LC-MS

Purine nucleotide measurement was performed as described previously with some modifications [67]. After adeno-associated virus or Cpd14-treated, the mice were euthanized by CO_2._ Tumor tissue was taken out and immediately placed in pre-chilled methanol for metabolomic analysis.

### Statistical analysis

The data are presented as the mean ± SD of at least three independent experiments. Statistical analyses were carried out by using GraphPad Prism 8 (San Diego, CA, USA). Pearson correlation analysis was utilized to evaluate the correlation between the two groups. Two-tailed Student’s *t* test was used to calculate *P* values. Kaplan–Meier curves for survival were analyzed with GraphPad software using the log-rank test. Statistical significance is displayed as *P* < 0.05.

## Supplementary information

Supplemental information

Extended Data Table 1

Extended Data Table 2

Extended Data Table 3

Extended Data Table 4

Extended Data Table 5

Extended Data Table 6

Extended Data Table 7

Extended Data Table 8

aj-checklist

Supplementary Figure 1

Supplementary Figure 2

Supplementary Figure 3

Supplementary Figure 4

Supplementary Figure 5

Supplementary Figure 6a-k

Supplementary Figure 6l,m

Supplementary Figure 7

Original data files

## Data Availability

Data from this study have been deposited in the GEO. Original data from RNA-seq in AOM/DSS-induced colon cancer model are available in the NCBI GEO under accession codes GSE189730. The datasets generated and/or analyzed during the current study are available from the corresponding author upon reasonable request.
