# Supplementary material for: p53 promotes peroxisomal fatty acid β-oxidation to repress purine biosynthesis and mediate tumor suppression
Source: Cell Death Dis. 2023 Feb 7;14(2):87. doi: 10.1038/s41419-023-05625-2 (PMC9905075; doi:10.1038/s41419-023-05625-2)
Supplement: Supplementary file 1 — Supplemental information [file 41419_2023_5625_MOESM1_ESM.docx]

**Supplemental Materials for**

**p53 Promotes Peroxisomal** **Fatty Acid β-oxidation to Repress Purine**

**Biosynthesis and Mediate Tumor Suppression**

**Figure S1. p53 facilitates peroxisomal β-oxidation genes expression**, **related to Figure 1.** (a) Representative image of tumor in the *Trp53^fl/fl^* and *VP* mice colon on day 98 after AOM injection. Colon sections were stained with hematoxylin & eosin (H&E). Scale bar, 20 μm. (b) Gene ontology analysis of gene sets that were significantly downregulated metabolic process in p53 null MEFs compared to WT ones. (c) GSEA was used to evaluate changes in the gene signature of peroxisomal FAO in p53 WT MEFs compared to null ones. (d) GSEA was used to evaluate changes in the gene signature of mitochondrial FAO in MEFs (upper) and CRC tumors (lower) from *Trp53^fl/fl^* and *VP* mice. (e) Heatmap analysis of peroxisomal FAO genes in p53 WT MEFs compared with null ones. (f) Peroxisomal FAO pathway genes were detected by qRT-PCR in HCT116 p53^+/+^ or HCT116 p53^-/-^ cells treated with Nutlin3 (10 μM), n = 3. (g) Western blot analysis for ACOX1, EHHADH, ACAA1, SCP2, ACOX3 and ABCD4 expression in HCT116 cells or MEFs treated with Nutlin3 or not; p53 knockdown (KD) in HCT116 p53^+/+^ or overexpression p53 in p53^-/-^. (h) Protein levels of ACOX1, EHHADH, ACAA1, SCP2, ACOX3 and ABCD4 in HCT116 p53^+/+^ and p53^-/-^ cells or p53^-/-^ stably expressing different types of p53 mutants. R, arginine; H, histidine; W, tryptophan.Data were presented as mean ± SD. ***P* < 0.01, ****P* < 0.001.

**Figure S2. p53 is associated with VDR to promote the expression of genes involved in peroxisomal FAO, Related to Figure 2.** (a) Protein expression of ACOX1, EHHADH, ACAA1, SCP2 and ABCD4 in CRC cells when VDR was knowdown. (b) Western blot analysis of ACOX1, EHHADH, ACAA1, SCP2, ACOX3 and ABCD4 expression in MEFs p53^+/+^ or p53^-/-^cells expressing VDR. (c) qPCR ChIP analyses of VDR binding to *ACOX1*, *EHHADH*, *ACAA1*, and *SCP2* promoter regions in MEFs p53^+/+^ or p53^-/-^cells, n = 3. (d) Western blot analysis of VDR, ACOX1, EHHADH, ACAA1, SCP2, ACOX3 and ABCD4 expression in HCT116 p53^+/+^ or HCT116 p53^-/-^cells treated with or without Nutlin3. (e) *VDR* expression was detected by qRT-PCR in HCT116 p53^+/+^ or HCT116 p53^-/-^ cells treated with Nutlin3 or not, n = 3. (f) Co-IP of endogenous p53 with VDR in CRC cells. (g) Schematic representation of the domains of p53 and VDR. TAD, N-terminal transactivation domains; DBD, DNA-binding domain; CTD, C-terminal domain. LBD, carboxy-terminal ligand binding domain; AF-2, activation function-2. (h) IP and WB of p53 interaction with VDR or the truncation mutants. (i) HCT116 and HEK293T cells were treated with Nutlin3 for 24h. Whole cell lysates and nuclear fractions were prepared. Data were presented as mean ± SD. ***P* < 0.01, ****P* < 0.001.

**Figure S3. Acetyl-CoA from p53-mediated peroxisomal FAO regulates ATIC activity, related to Figure 3.** (a) Levels of H_2_O_2_ in the CRC tumors from CRC mice tumor tissues and HCT116 cells, n = 3 or 6. (b) Western blot analysis of ACLY, ACSS2, and ACOX1 expression in CRC cells treated with Nutlin3. (c) GSEA plot showing enriched nucleotide biosynthesis signaling pathways in the *VP* mouse. (d) Western blot analysis of ATIC acetylation in CRC cells or MEFs treated with Nutlin3 or not. (e) Western blot analysis of ATIC acetylation in CRC cells stably expressing Flag-ATIC were treated with Nutlin3 (left), or transfected with HA-p53 (right). (f) HCT116 cells with a stable knockdown of ACOX1 were subsequently infected with viruses for expression of ACOX1. The above cells followed by the western blot analysis of ATIC acetylation and measurement of acetyl-CoA levels, n = 3. (g) ATIC activity was measured in HCT116 cells treated with or without Nutlin3, n = 3. (h) Effect of ATIC K266R, K356R, or K524R mutation on its acetylation and activity, n = 3. (i) The amino acid sequences surrounding ATIC K266 in multiple species. (j) HCT116 cells with a stable knockdown of ATIC were subsequently infected with viruses for expression of Flag-ATIC (WT), Flag- ATIC -K266R (K266R) or Flag- ATIC -K266Q (K266Q). The above cells were further treated with Nutlin3, followed by the western blot analysis of ATIC acetylation and measurement of ATIC enzyme activity, n = 3. Data were presented as mean ± SD. ns, non-significant, *P* > 0.05.

**Figure S4. p53 increases ATIC acetylation to trigger its degradation by transcriptionally activating KAT2B, related to Figure 4.** (a) HIEC cells were treated with either DMSO or Nutlin3 for 48 h, and the expression of 13 KAT family acetyltransferases genes were assessed by qRT-PCR, n = 3. (b) Protein expression of KAT2A and KAT2B in HCT116 and HIEC cells treated with Nutlin3 for 48 h. (c) Western blot analysis of ATIC acetylation in CRC cells when overexpression of KAT2B. (d) Effect of ATIC K266R, K356R, or K524R mutation on KAT2B-mediated ATIC acetylation. (e) Western blot analysis of ATIC acetylation in HCT116 cells when KAT2B KD or co-expression of Flag-KAT2B. (f) Co-IP of endogenous KAT2B with ATIC in CRC cells. (g) mRNA levels of *TP53* and *ATIC* in HCT116 p53^+/+^ and p53^-/-^ cells treated with either DMSO or Nutlin3 for 48 h were analyzed by qRT-PCR, n = 3. (h) Western blot analysis of ATIC in HCT116 cells treated with or without Nutlin3 for 24 h. After addition of 100 mg/mL cycloheximide (CHX), cells were harvested at the indicated times for IB analysis. Signal intensity of ATIC protein was quantified by Image J, and then normalized to β-Actin band intensity. (i) Western blot analysis of HCT116 cells stable knockdown of endogenous ATIC and re-introduced with ATIC WT, K266R or K266Q mutant. After addition of 100 mg/mL CHX, cells were harvested at the indicated times for IB analysis. (j) CRC cells treated with or without Nulin3 for 24 h were further cultured in the presence of DMSO or 10 μM CQ for 10 h, followed by western blot analysis of ATIC expression. Data were presented as mean ± SD. ***P* < 0.01, ****P* < 0.001.

**Figure S5. Acetylation of ATIC is closely correlated to colorectal tumorigenesis, related to Figure 5.** (a) ATIC immunohistochemistry (IHC) in representative sections of tumor tissues from *VP* mice. (b) Western blot analysis of ATIC following injection of AAV2-Atic (WT, K266R, K266Q) in CRC tumors from *Trp53^fl/fl^* and *VP* mice, n = 3. (c) CCK-8 assays were performed in CRC cells stably expression the indicated vectors, n = 3. (d) HCT116 cells with stable expression of p53 were further infected with viruses expressing ATIC WT, K266R or K266Q mutant. Cell growth curves were determined by CCK-8 assays, n=3. (e) ATIC activity was measured in the above cells from (a), n=3. (f) ATP levels were measured with assay kits in the above cells from (a), n=3. Data were presented as mean ± SD.

**Figure S6. Targeting ATIC suppresses colorectal tumorigenesis, related to Figure 6****.** (a) A scheme of Cpd14 treatment in *APC^min/+^Trp53^fl/fl^* and *AP* CRC mouse model. (b) Typical images of colon tumors from Control and Cpd14-treated mice 42 days after Cpd14 treatment. (c) Colon tumor volume in mice from (a), n = 5 or 6. Tumor volume (*TV*) was calculated was calculated as follows: *TV* = 3.14 × d^3^ × 0.75. (d) Tumor inhibition rate for Cpd14-treated mice relative to control mice in p53 WT and p53-deficient mice, n = 5 or 6. (e) ATIC activity (left) and ATP levels (right) in tumor tissues from in *APC^min/+^ Trp53^fl/fl^* and *AP* CRC mouse model, n = 5 or 6. (f) A Scheme for the AOM/DSS-induced colon cancer model in *Trp53^fl/fl^* and *VP* mice (top). Each mouse received two intraperitoneal injections of the indicated virus during the second processing of DSS treatment. Typical images of colon tumors from mice treated with shCtrl or shAtic encoding AAV. (g) ATIC immunohistochemistry (IHC) in representative sections of tumor tissues from each group, as in (f). (h) Western blot analysis of ATIC following injection of the indicated virus in CRC tumors from *Trp53^fl/fl^* and *VP* mice, n = 2. (i) Colon tumor numbers in mice from (f), n = 6. (j) Tumor inhibition rate for shAtic mice relative to Ctrl mice in *Trp53^fl/fl^* and *VP* mice, n = 6. (k) ATIC activity and ATP levels were measured in tumor tissues from (f), n = 6. (l) Cell growth were determined by CCK-8 knockdown of ATIC, n = 3. (m) ATIC activity and ATP levels were measured in the above cells from (l), n = 3. Data were presented as mean ± SD.

**Figure S7. Acetylation of ATIC is downregulated in human CRCs, related to Figure 7.** (a) Western blot analysis of the indicated proteins in human CRC patient tissues, 30 pairs of CRC samples (T) with adjacent normal colon tissues (N). (b) *ACOX1*, *KAT2B*, *ATIC* expression in CRC tumors and normal colorectal tissues. The raw data from GSE41258, and GSE23878. (c) Protein expression of ACOX1 and ATIC in colon cancer. KAT2B was not identified in Colon data set. Z-values represent standard deviations from the median across samples for the given cancer type. Log2 Spectral count ratio values from CPTAC were first normalized within each sample profile, then normalized across samples. The data was from CPTAC.

**Key resources table**

| **Antibodies** | **SOURCE** | **IDENTIFIER** |
| --- | --- | --- |
| VDR | Proteintech | 14526-1-AP |
| VDR | Santa Cruz | sc-13133 |
| p53 | Abclonal | A11232 |
| p53 | Santa Cruz | sc-126 |
| ACOX1 | Abcam | ab184032 |
| EHHADH | Abclonal | A13488 |
| ACAA1 | Abclonal | A7422 |
| ACOX3 | Abclonal | A17020 |
| p21 | Abclonal | A19094 |
| SCP2 | Proteintech | 23006-1-AP |
| H3 | Proteintech | 17168-1-AP |
| ABCD4 | Elabscience | E-AB-12686 |
| ATIC | Abclonal | A5559 |
| ATIC | Abcam | ab188321 |
| KAT2A | Abclonal | A2224 |
| KAT2B | Proteintech | 13983-1-AP |
| KAT2B | Abclonal | 13983-1-AP |
| ACLY | Proteintech | 15421-1-AP |
| ACSS2 | Abclonal | A12334 |
| Acetylated-Lysine Antibody | CST | AB_331805 |
| HA | Sigma | H6908 |
| HA | OriGene | AF4911 |
| Flag | Sigma | AB_259529 |
| Flag | Proteintech | 20543-1-AP |
| GFP | Proteintech | 66002-1-Ig |
| β-actin | Santa Cruz | Sc47778 |
| Goat Anti-Rabbit IgG (alexa fluor 594) | Abcam | Ab150080 |
| Goat Anti-Mouse IgG (alexa fluor 488) | Abcam | Ab150113 |
| HRP- Mouse Anti-Rabbit IgG Light chain specific | Proteintech | SA00001-7L |
| HRP-conjugated Anti-Heavy Chain of Rabbit IgG | Proteintech | SA00001-7H |
| HRP-conjugated Goat Anti-Mouse IgG Heavy Chain | Abclonal | AS064 |
| HRP-conjugated AffiniPure Goat Anti-Mouse IgG Light Chain | Abclonal | AS062 |

**Critical Commercial Assays**

| Acetyl-Coenzyme A Assay Kit | Sigma | MAK039 |
| --- | --- | --- |
| Hydrogen Peroxide Assay Kit | Solarbio | BC3595 |
| BCA Assay | Thermo Fisher | 23225 |
| Dual-Luciferase® Reporter Assay System | Promega | E1910 |
| Cell Counting Kit-8 | TOPSCIENCE | C0005 |
| ATP assay kit | Njjcbio | A095-1-1 |
| Nuclear and Cytoplasmic Protein Extraction Kit | Beyotime | P0027 |

**Experimental Models: Cell Lines**

| Human embryonic kidney HEK293T cells | ATCC | CRL-3216 |
| --- | --- | --- |
| Human colorectal carcinoma HCT116 cells | ATCC | CCL-247 |
| Human colorectal carcinoma HCT116 p53^-/-^cells | Prof. zhang Lab | ^1^ |
| Human colorectal carcinoma RKO cells | ATCC | CRL-2577 |
| Human normal intestinal epithelial HIEC cells | ATCC | CRL-3266 |
| Mouse embryo fibroblasts (MEFs) | This paper | N/A |

**Experimental Models: Organisms/Strains**

| C57B6/J mice | NRCMM | NA |
| --- | --- | --- |
| *Trp53^fl/fl^* | Prof. Zhong Lab | ^2^ |
| *Pvillin-Cre* | Cyagen | C001014 |
| *Trp53^em1Cd^/Gpt* | GemPharmatech | T005332 |
| BALB/c nude mice | GemPharmatech | D000521 |

**Chemicals and Reagent**

| PMSF | Calbiochem | 80055-380 |
| --- | --- | --- |
| Triton X-100 | Thermo Fisher Scientific | 28314 |
| Trypsin-EDTA | Gibco | 15400054 |
| Fetal Bovine Serum (FBS) | Gibco | 26010074 |
| Penicillin/Streptomycin | Gibco | 15140-122 |
| DMSO | Sigma-Aldrich | D2650 |
| Protein G Sepharose | Sigma-Aldrich | P3296 |
| Nutlin3 | Sigma-Aldrich | SML0580 |
| Cycloheximide (CQ) | Sigma-Aldrich | R750107 |
| 3-methyladenine (3-MA) | Sigma-Aldrich | M9281 |
| MG132 | Sigma-Aldrich | M8699 |
| 10-Formylfolic acid | Sigma-Aldrich | 1286300 |
| Azoxymethane | Sigma-Aldrich | A5486 |
| TRIzol | Life Technology | 15596018 |
| Palmitic Acid-^13^C16 | Medical Isotopes, Inc. | C99 |
| Docosanoic Acid (d43) | CIL | DLM-4703-PK |
| Docosanoic-22,22,22-d3 Acid | Medical Isotopes, Inc. | D1936 |
| NAD^+^ | MCE | HY-B0445 |
| Chloroquine | MCE | HY-17589A |
| 5-Fluorouracil | MCE | HY-90006 |
| AICAR | TargetMol | T1477 |
| Etomoxir | TargetMol | T0365 |
| Thioridazine | TargetMol | T4535 |
| Dextran Sulfate Sodium Salt (DSS) | MPBIO | 160110 |

**References**

1 Tu, R. *et al.* USP49 participates in the DNA damage response by forming a positive feedback loop with p53. *Cell Death Dis* **9**, 553 (2018).

2 Wang, X.-M. *et al.* The deubiquitinase USP25 supports colonic inflammation and bacterial infection and promotes colorectal cancer. *Nature Cancer* **1**, 811-825 (2020).
