# Supplementary figures and images for: p53 promotes peroxisomal fatty acid β-oxidation to repress purine biosynthesis and mediate tumor suppression
Source: Cell Death Dis. 2023 Feb 7;14(2):87. doi: 10.1038/s41419-023-05625-2 (PMC9905075; doi:10.1038/s41419-023-05625-2)

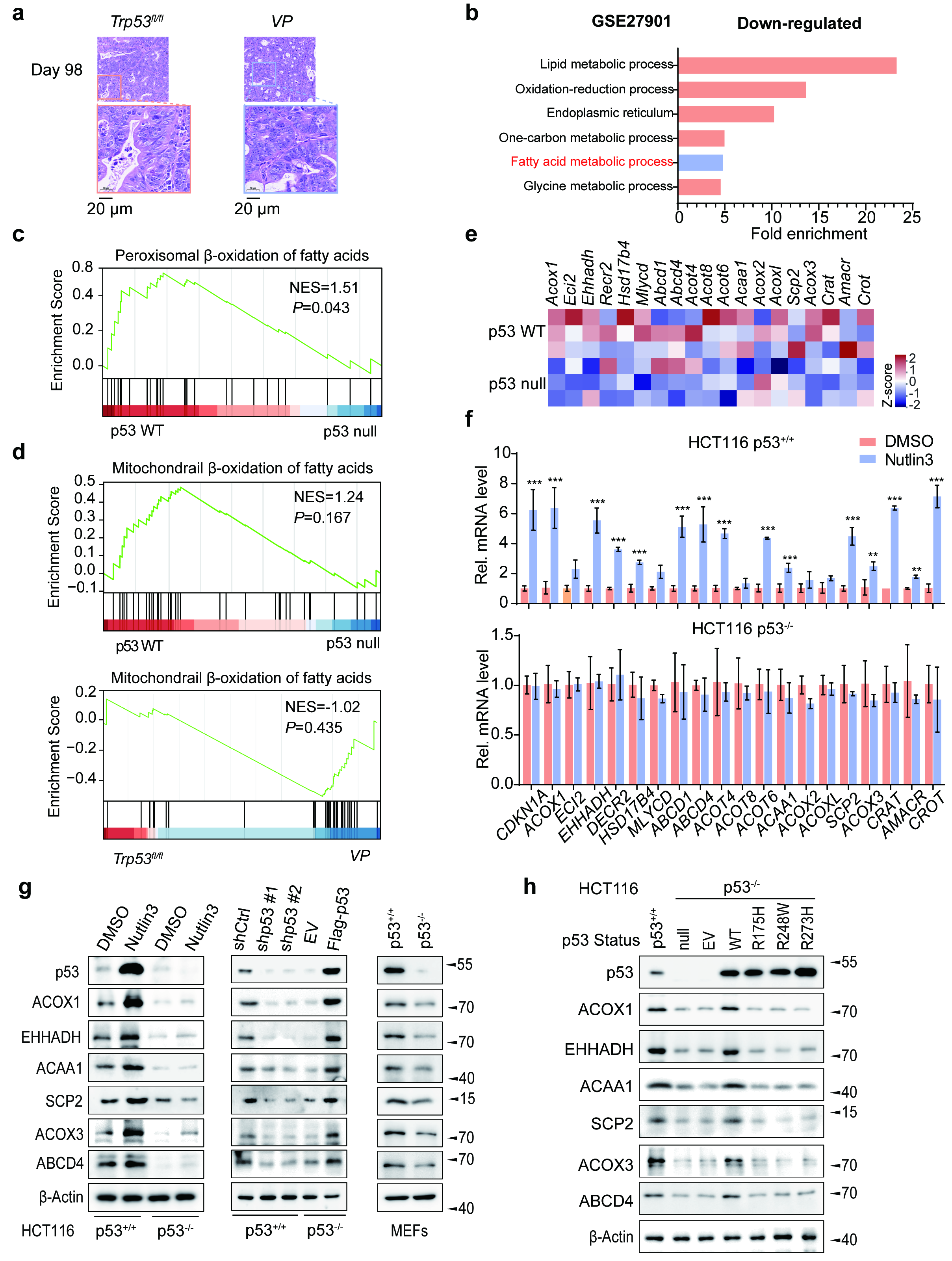

Supplement: Supplementary file 11 — Supplementary Figure 1 [file 41419_2023_5625_MOESM11_ESM.tif]

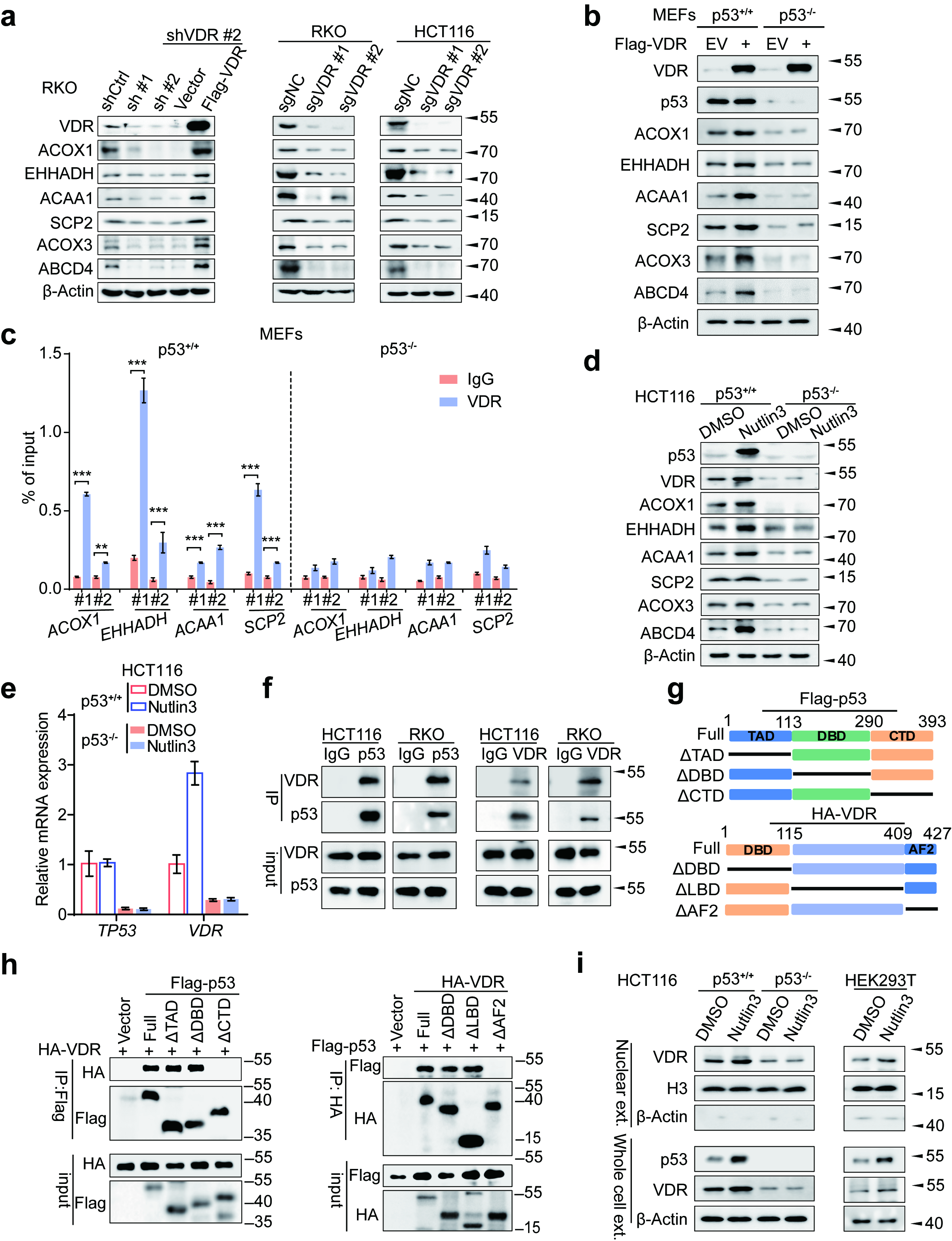

Supplement: Supplementary file 12 — Supplementary Figure 2 [file 41419_2023_5625_MOESM12_ESM.tif]

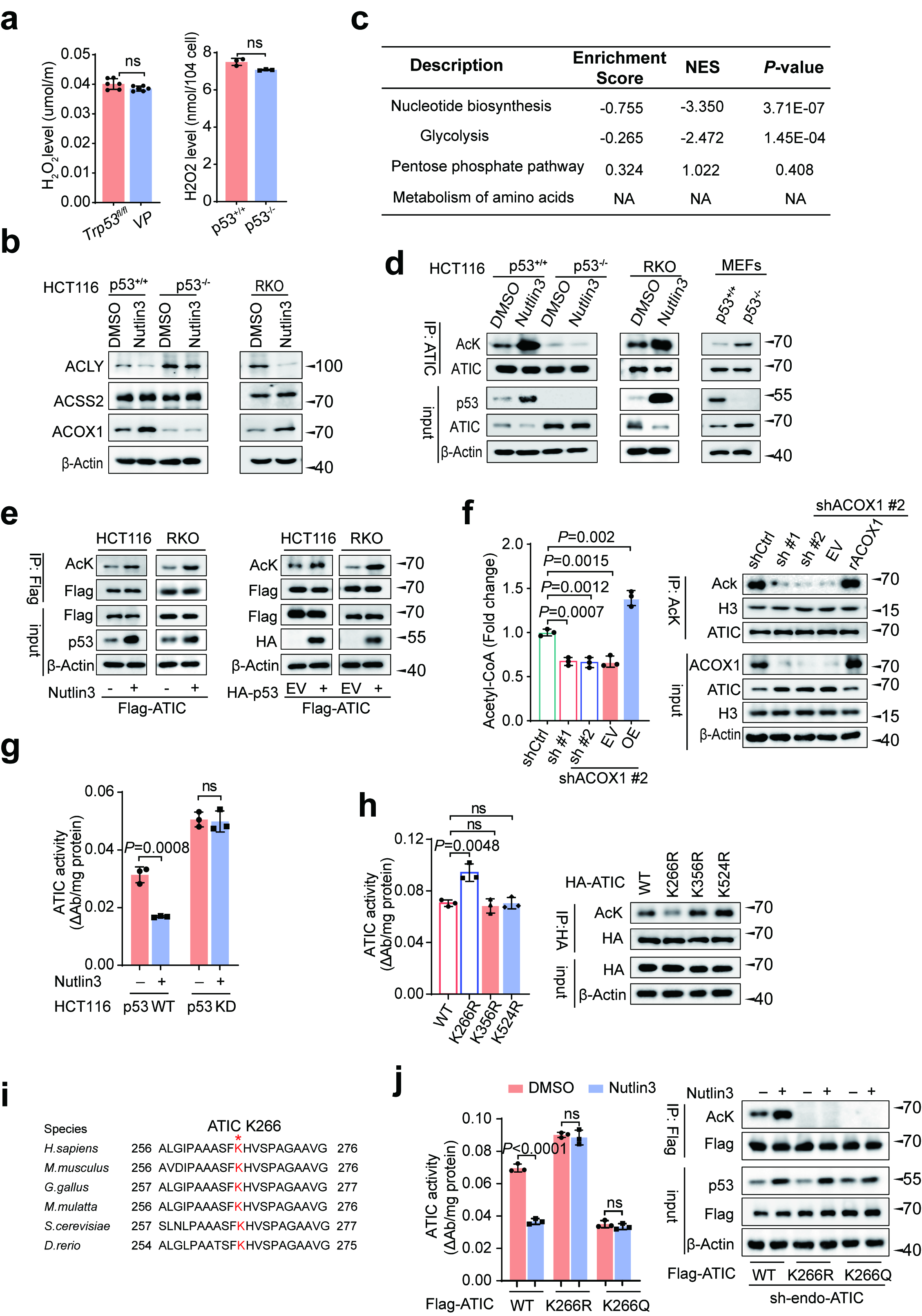

Supplement: Supplementary file 13 — Supplementary Figure 3 [file 41419_2023_5625_MOESM13_ESM.tif]

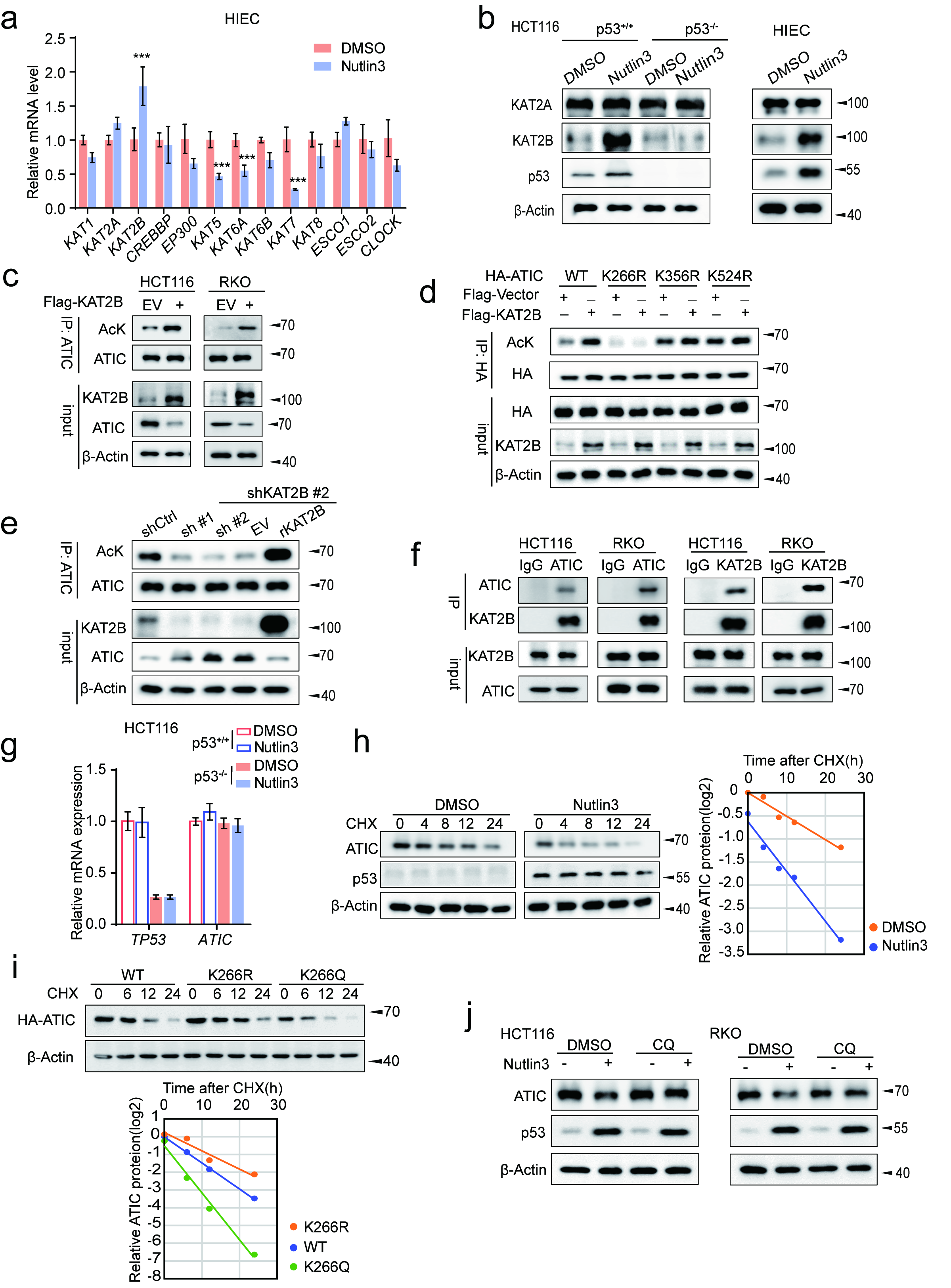

Supplement: Supplementary file 14 — Supplementary Figure 4 [file 41419_2023_5625_MOESM14_ESM.tif]

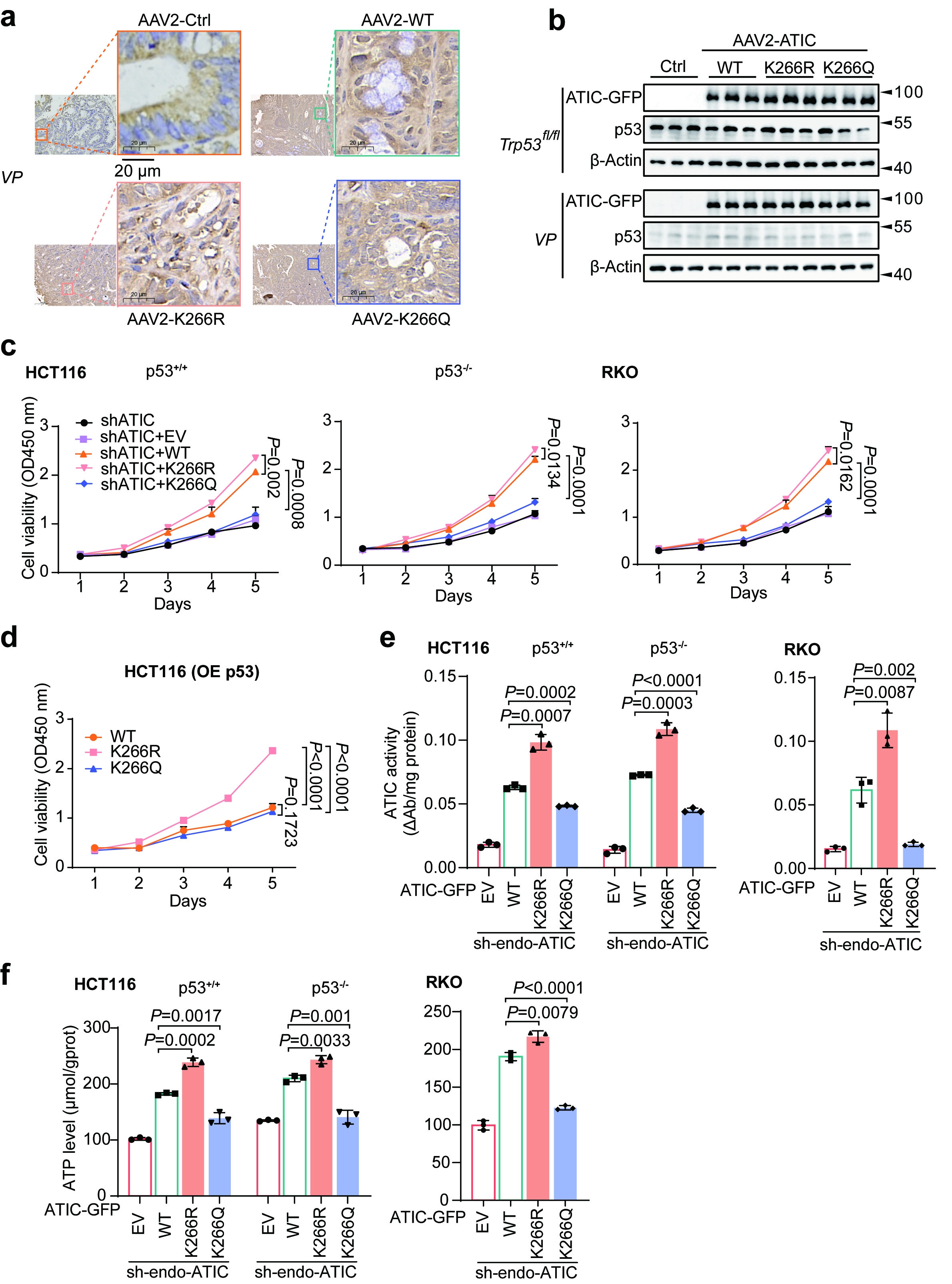

Supplement: Supplementary file 15 — Supplementary Figure 5 [file 41419_2023_5625_MOESM15_ESM.tif]

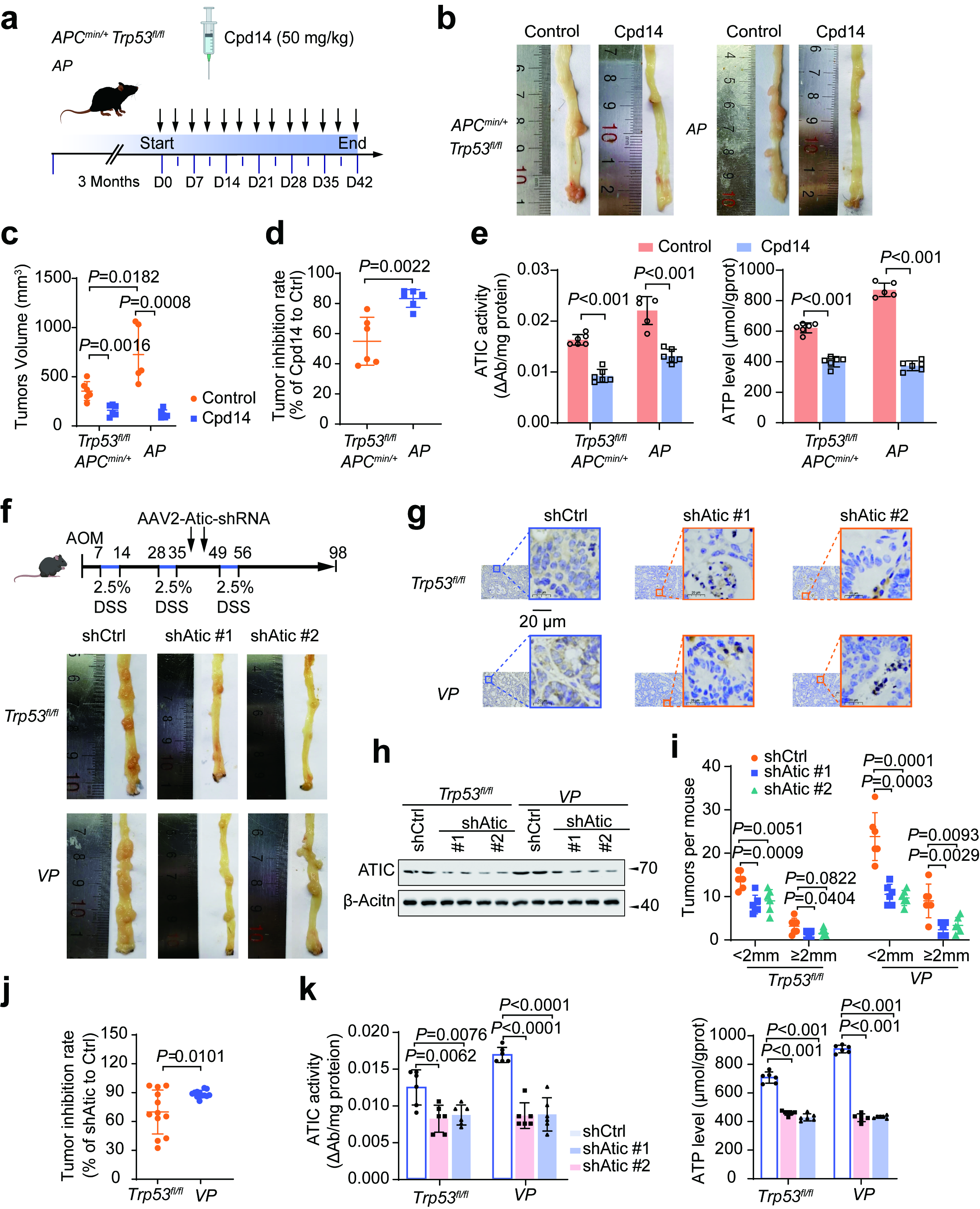

Supplement: Supplementary file 16 — Supplementary Figure 6a-k [file 41419_2023_5625_MOESM16_ESM.tif]

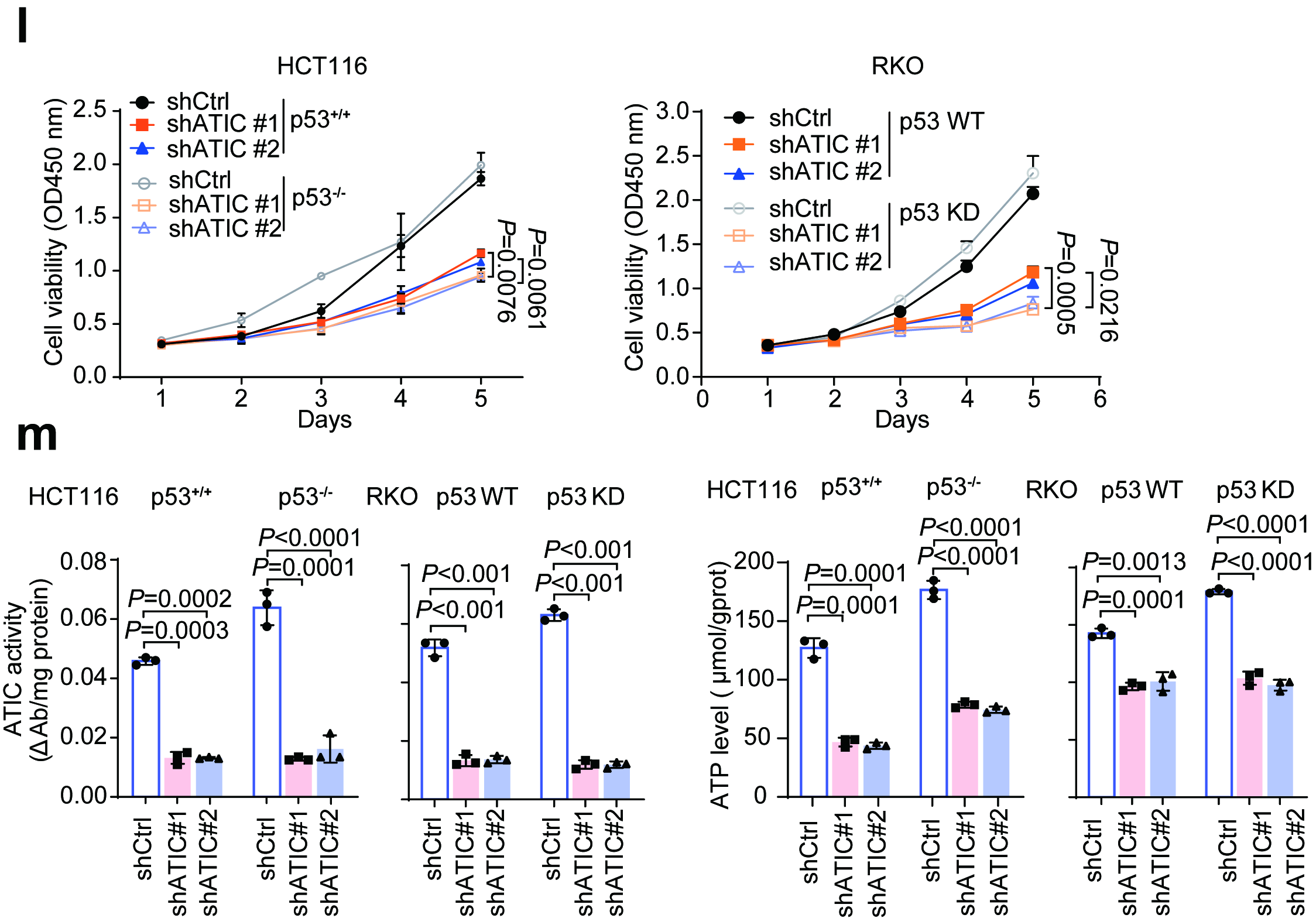

Supplement: Supplementary file 17 — Supplementary Figure 6l,m [file 41419_2023_5625_MOESM17_ESM.tif]

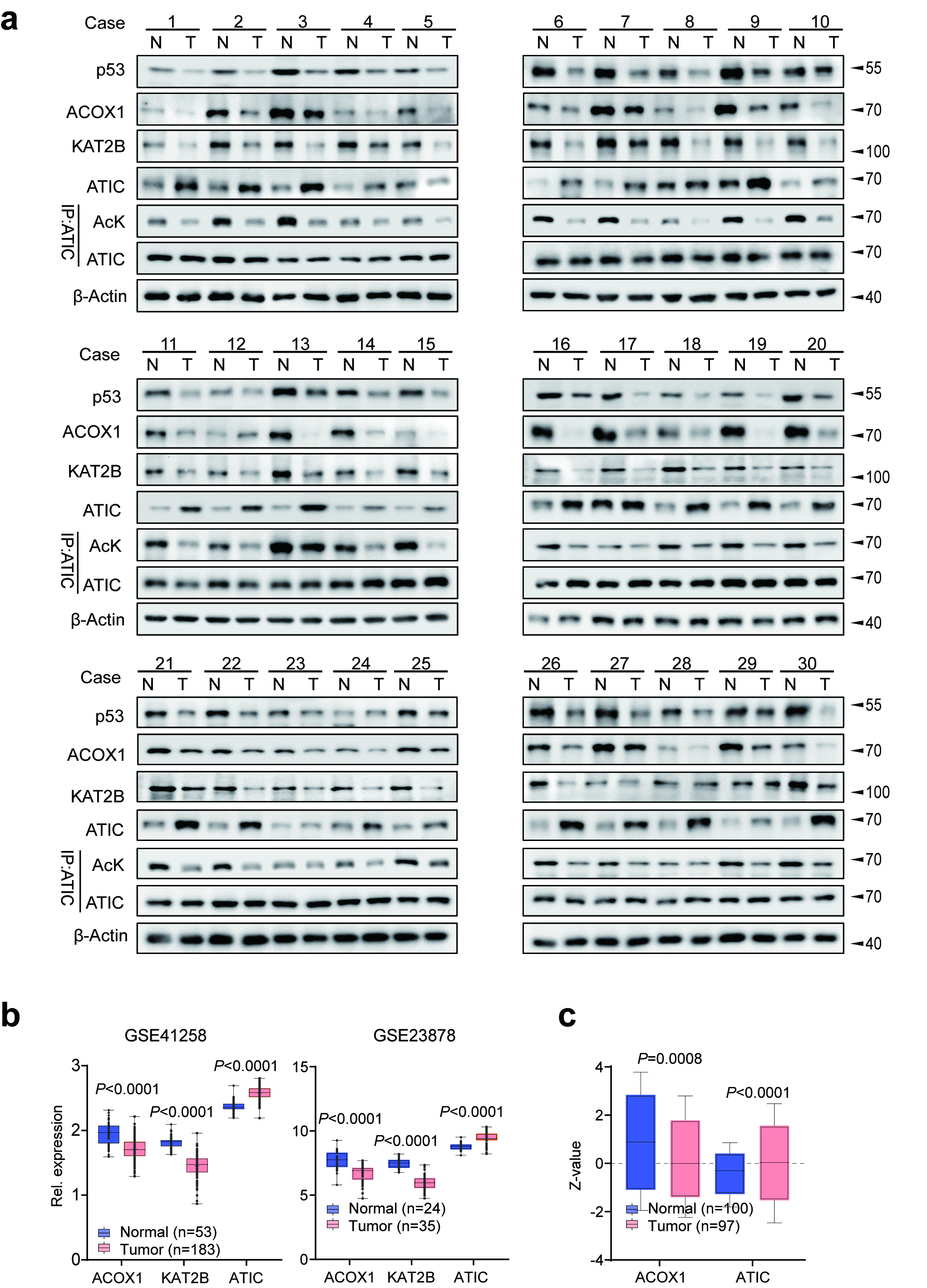

Supplement: Supplementary file 18 — Supplementary Figure 7 [file 41419_2023_5625_MOESM18_ESM.tif]
