# Supplementary material for: p53 promotes peroxisomal fatty acid β-oxidation to repress purine biosynthesis and mediate tumor suppression
Source: Cell Death Dis. 2023 Feb 7;14(2):87. doi: 10.1038/s41419-023-05625-2 (PMC9905075; doi:10.1038/s41419-023-05625-2)

Fig. 1f

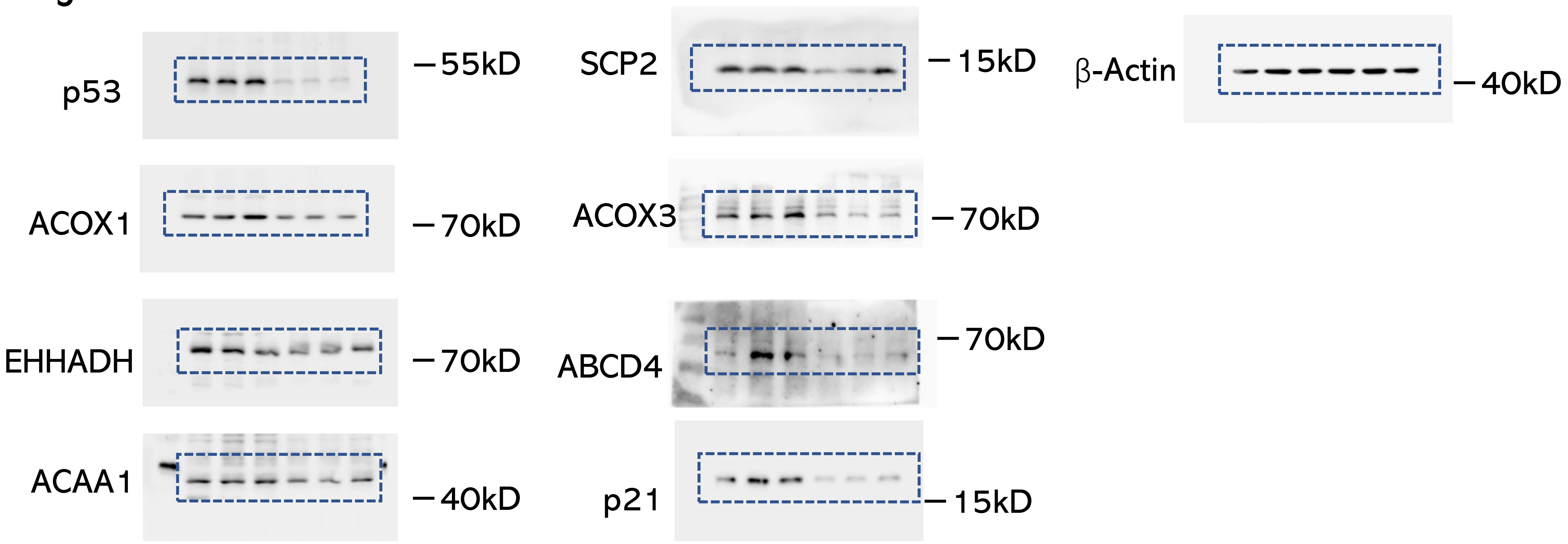
p53
55kD
SCP2
15kD
β-Actin
40kD
ACOX1
70kD
ACOX3
70kD
EHHADH
70kD
ABCD4
70kD
ACAA1
40kD
p21
15kD

Fig. S1g

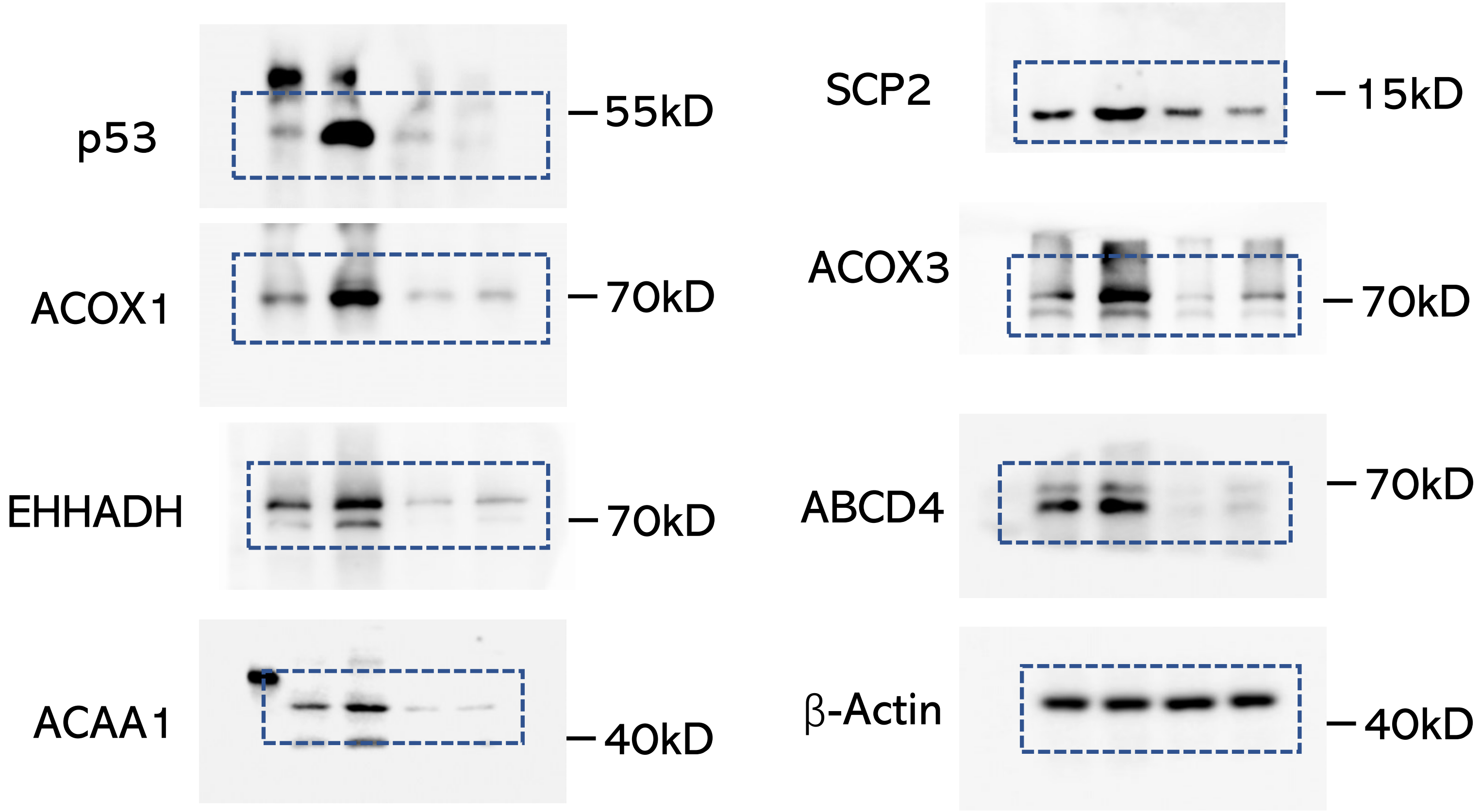
p53
55kD
ACOX1
70kD
EHHADH
70kD
ACAA1
40kD
SCP2
15kD
ACOX3
70kD
ABCD4
70kD
β-Actin
40kD

Fig. S1g

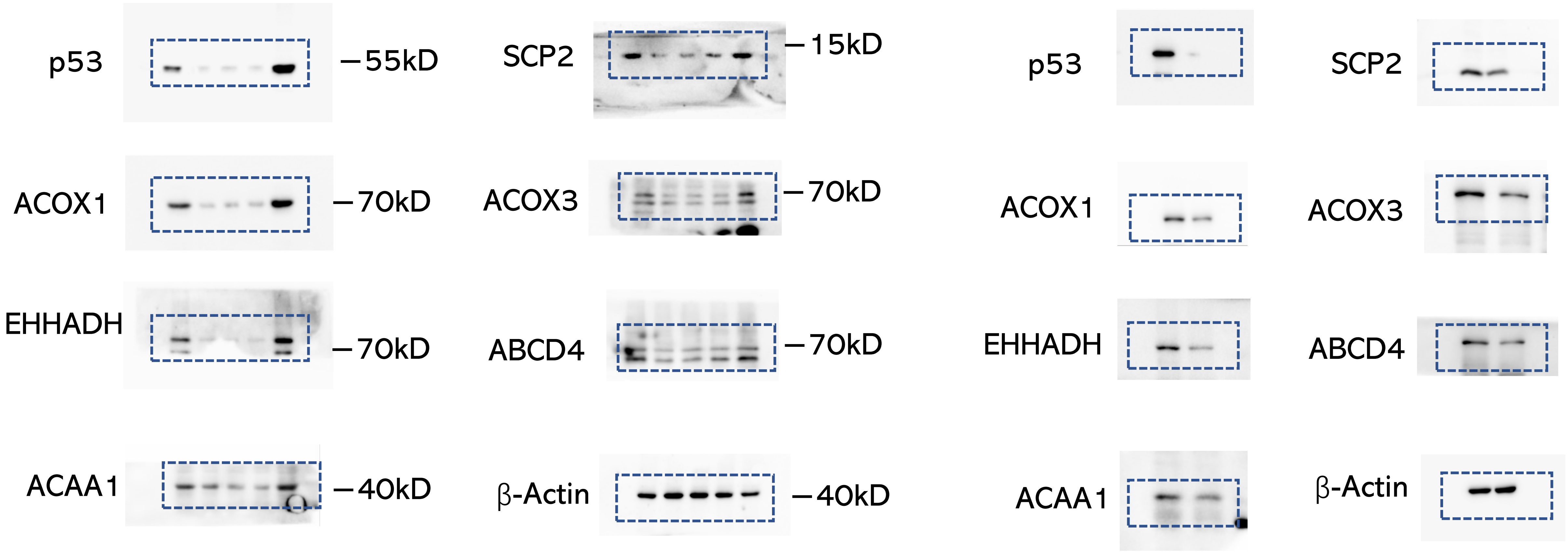
p53
—55kD
SCP2
—15kD
p53
SCP2
ACOX1
—70kD
ACOX3
—70kD
ACOX1
ACOX3
EHHADH
—70kD
ABCD4
—70kD
EHHADH
ABCD4
ACAA1
—40kD
β-Actin
—40kD
ACAA1
β-Actin

Fig. S1h

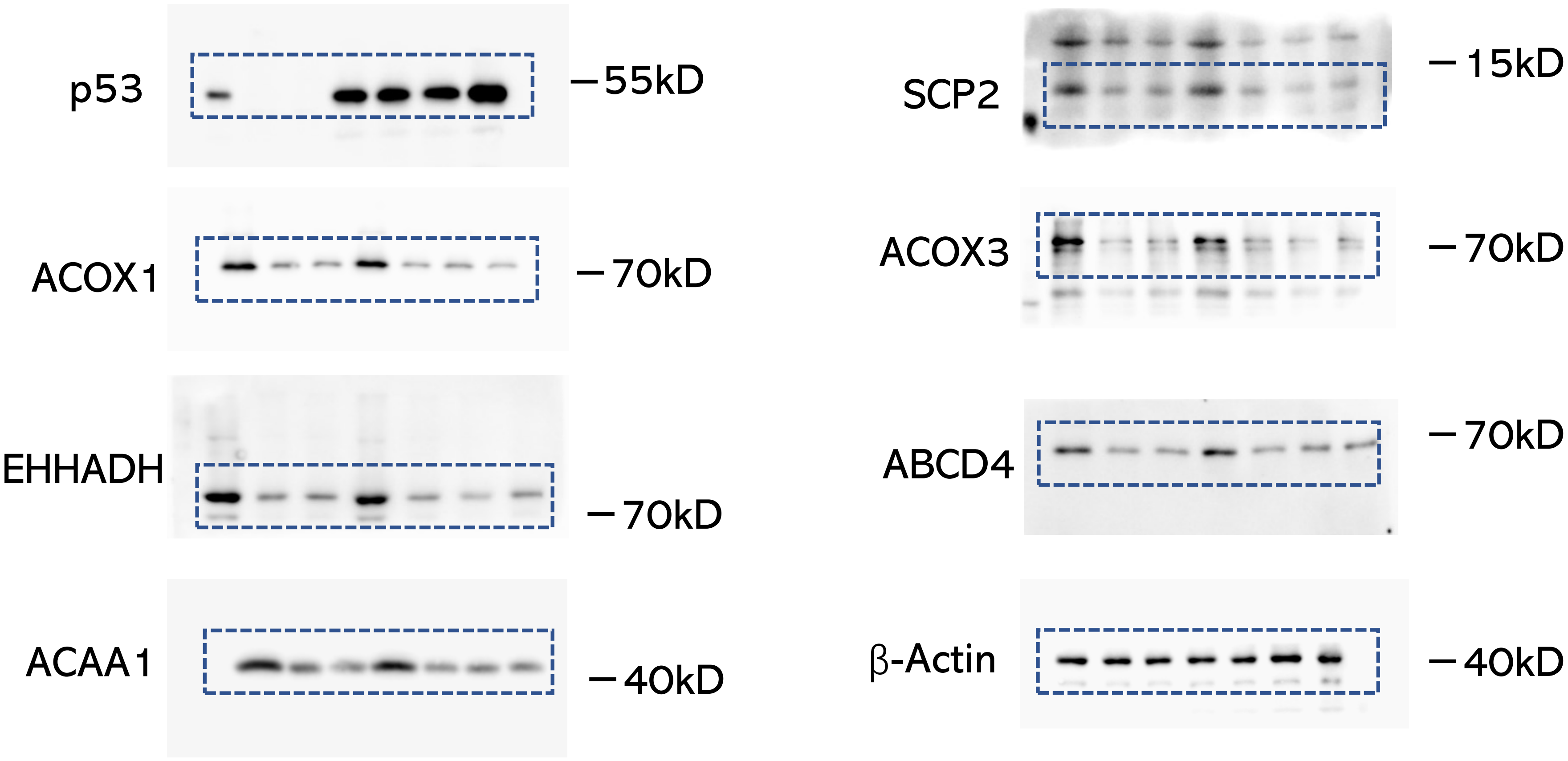
p53
55kD
ACOX1
70kD
EHHADH
70kD
ACAA1
40kD
SCP2
15kD
ACOX3
70kD
ABCD4
70kD
β-Actin
40kD

Fig. 2c

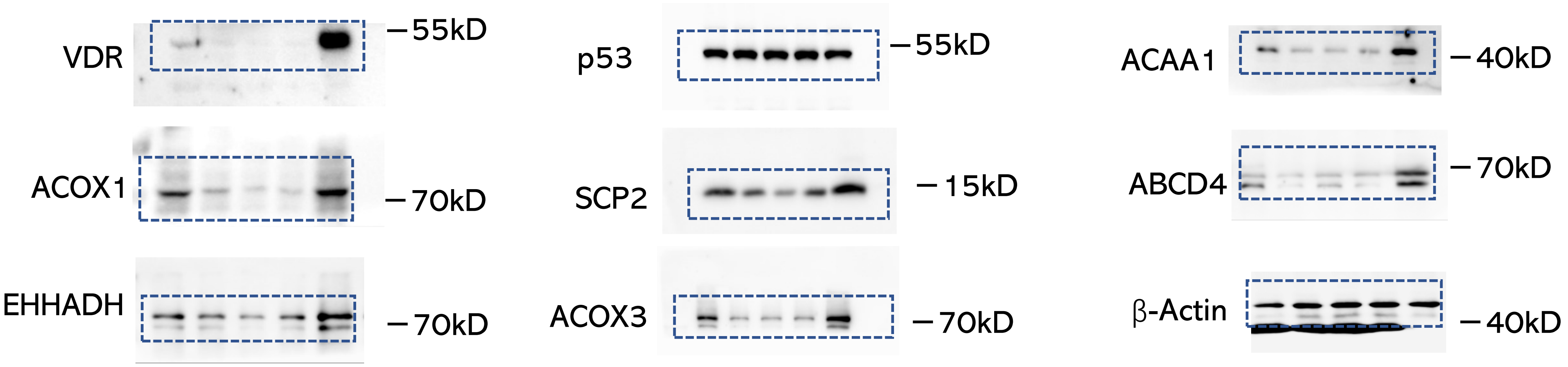
VDR
55kD
p53
55kD
ACAA1
40kD
ACOX1
70kD
SCP2
15kD
ABCD4
70kD
EHHADH
70kD
ACOX3
70kD
β-Actin
40kD

Fig. 2d

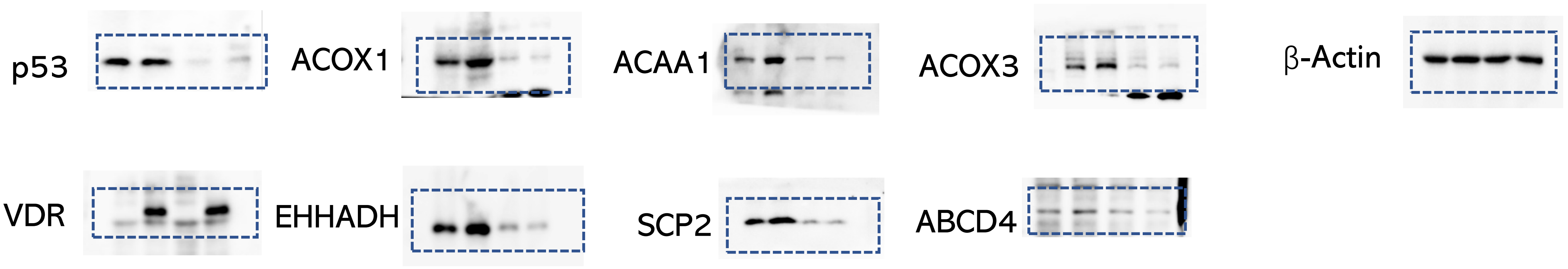
p53
ACOX1
ACAA1
ACOX3
β-Actin
VDR
EHHADH
SCP2
ABCD4

Fig. S2a

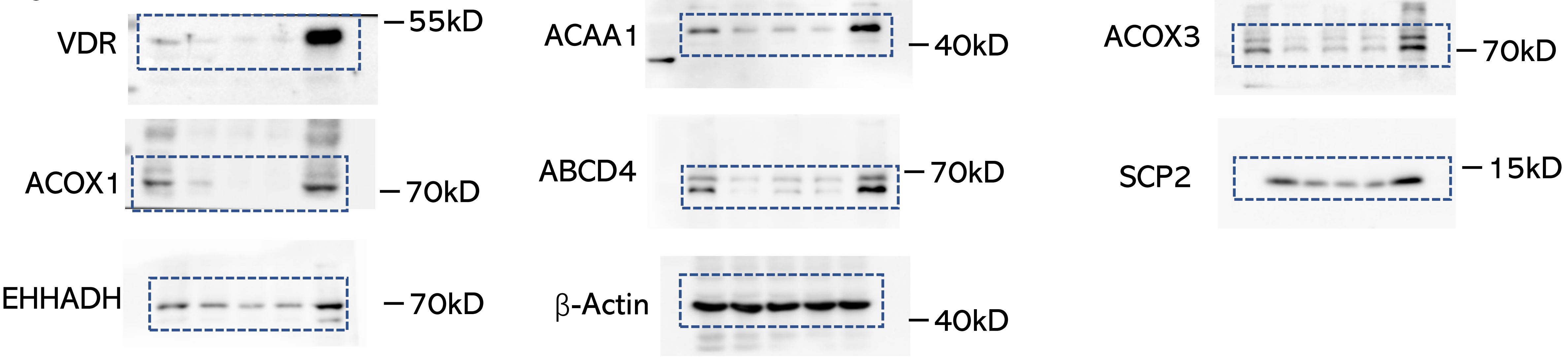

Fig. S2a

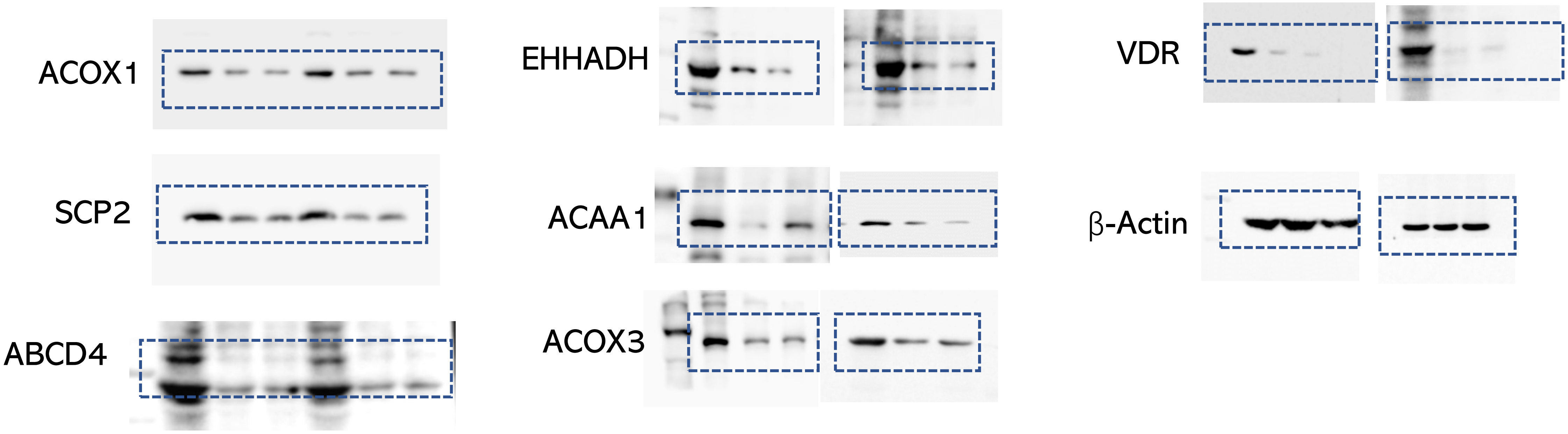

Fig. S2b

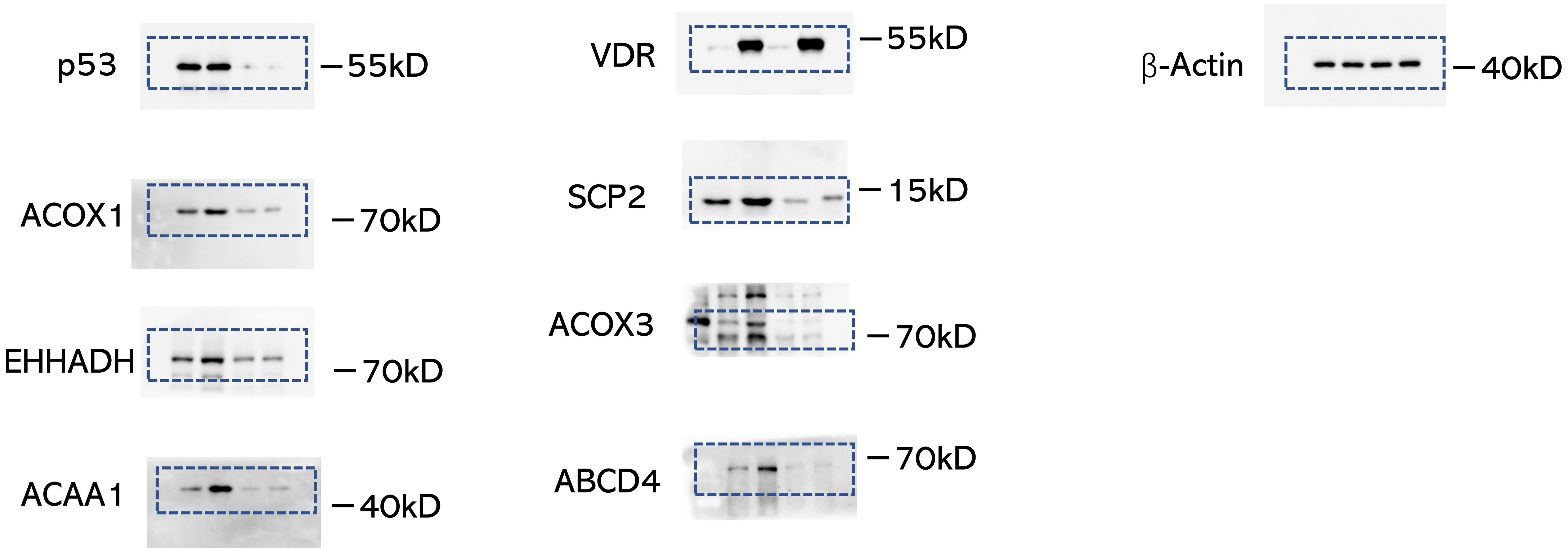
p53
55kD
VDR
55kD
β-Actin
40kD
ACOX1
70kD
SCP2
15kD
EHHADH
70kD
ACOX3
70kD
ACAA1
40kD
ABCD4
70kD

Fig. S2d

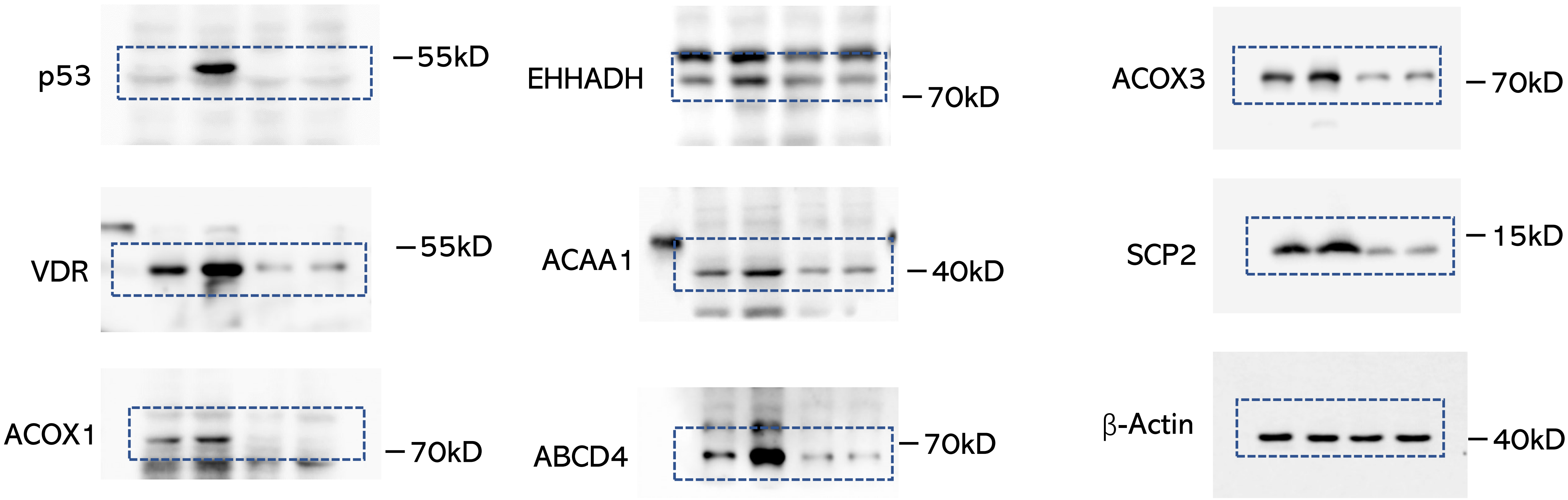

p53
55kD
EHHADH
70kD
ACOX3
70kD
VDR
55kD
ACAA1
40kD
SCP2
15kD
ACOX1
70kD
ABCD4
70kD
β-Actin
40kD

Fig. S2f

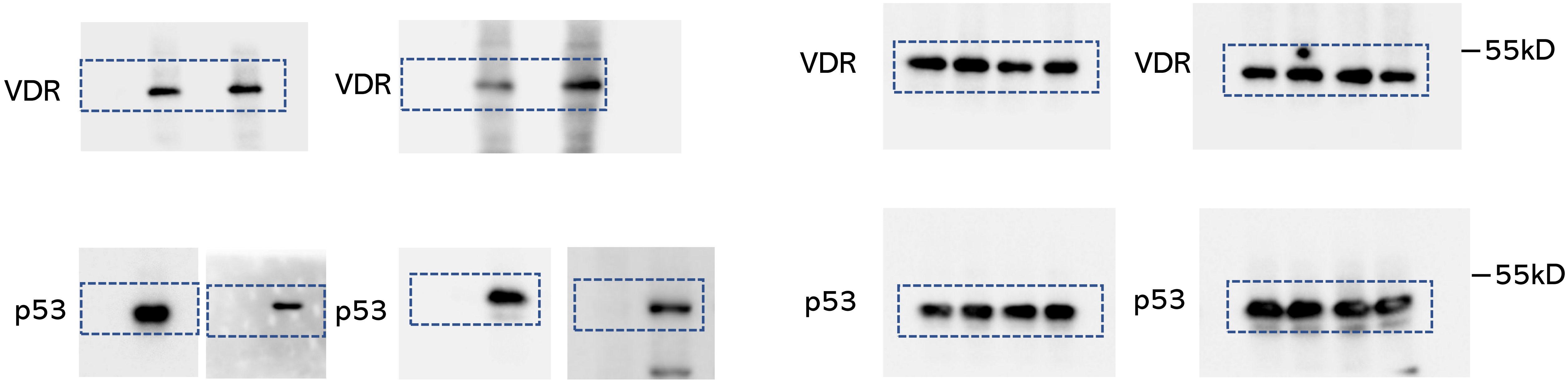

Fig. S2h

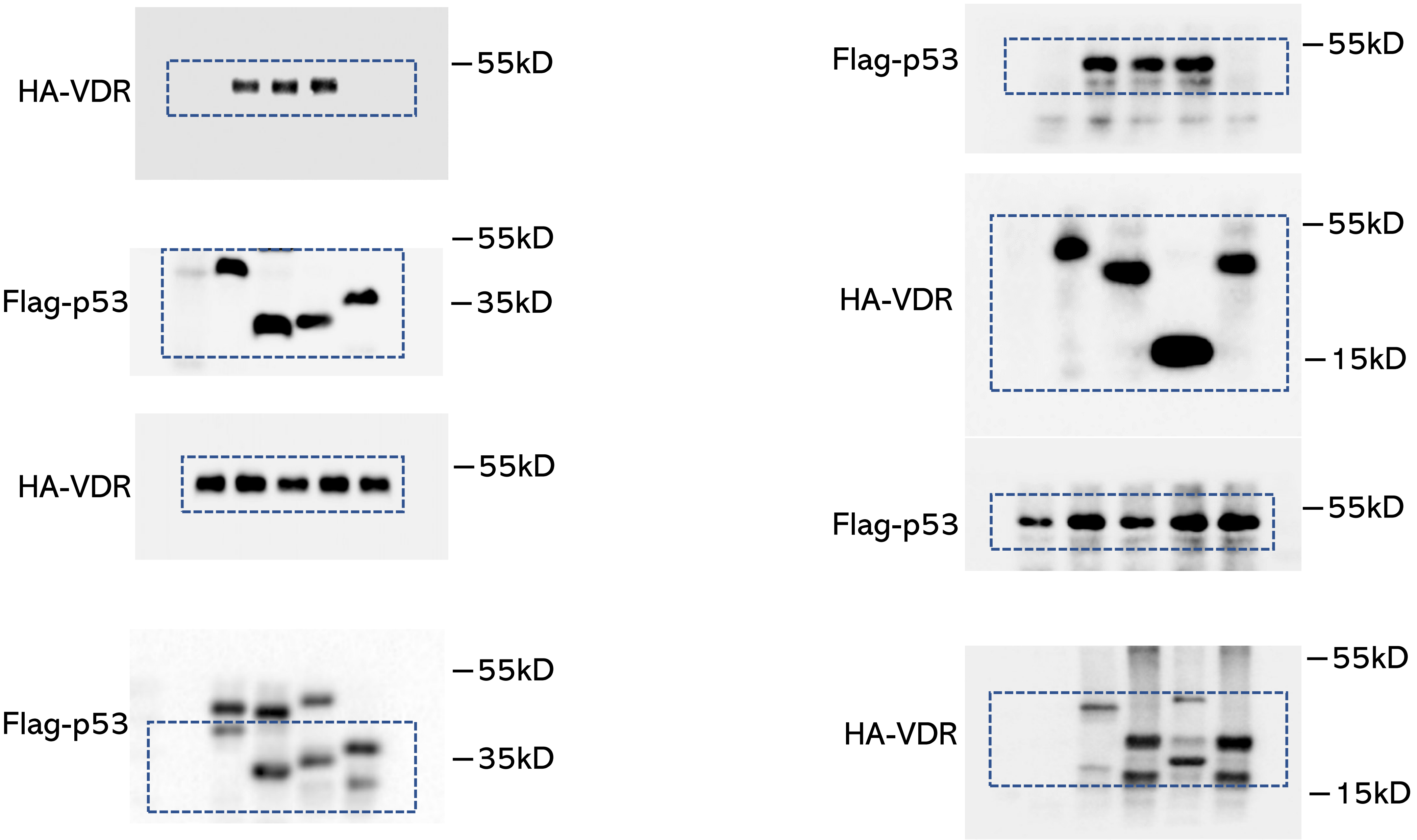
HA-VDR
55kD
Flag-p53
55kD
35kD
HA-VDR
55kD
Flag-p53
55kD
35kD
Flag-p53
55kD
HA-VDR
55kD
15kD
Flag-p53
55kD
HA-VDR
55kD
15kD

Fig. S2i

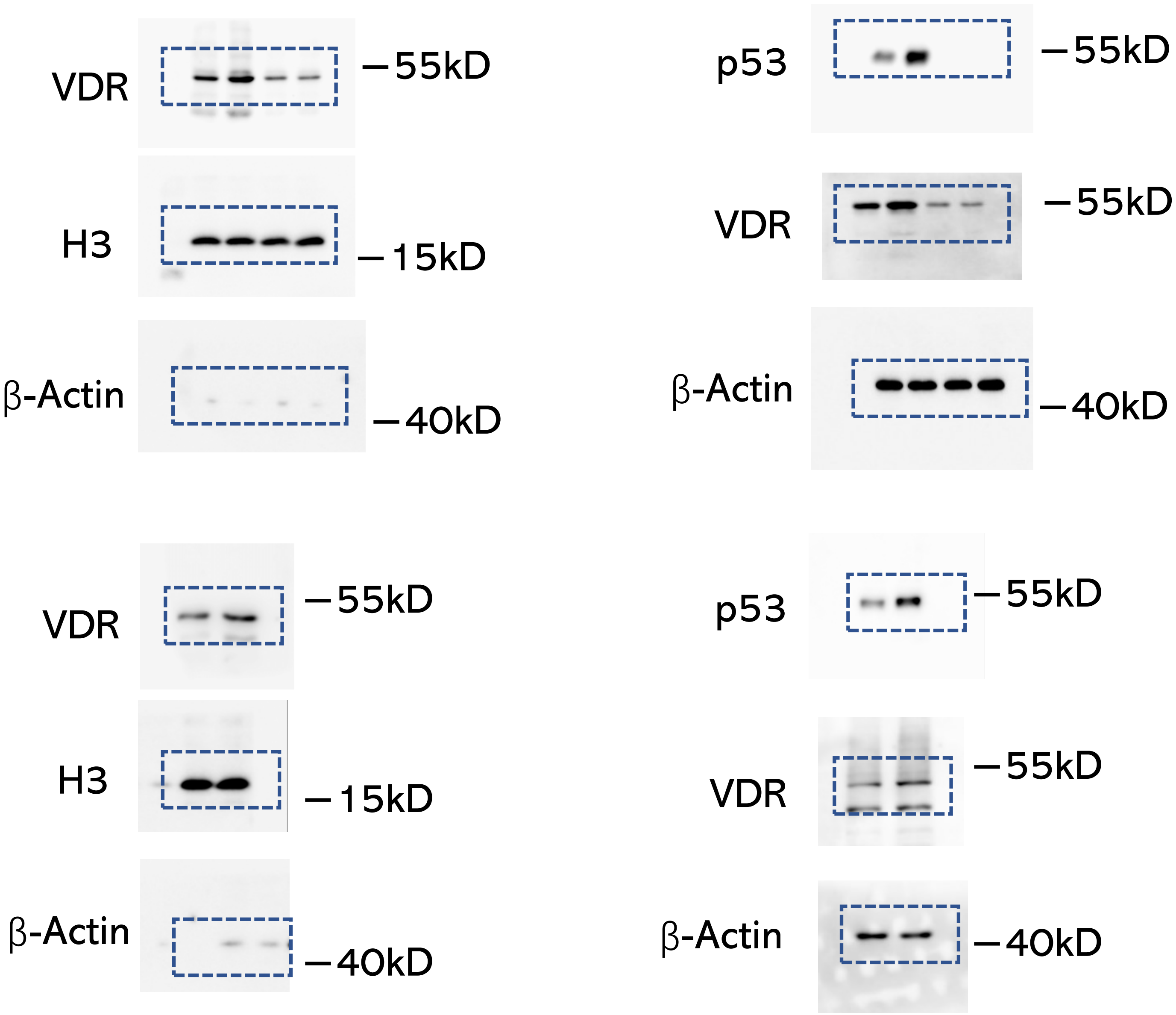
VDR
—55kD
H3
—15kD
β-Actin
—40kD
p53
—55kD
VDR
—55kD
β-Actin
—40kD
VDR
—55kD
H3
—15kD
β-Actin
—40kD
p53
—55kD
VDR
—55kD
β-Actin
—40kD

Fig. 3e

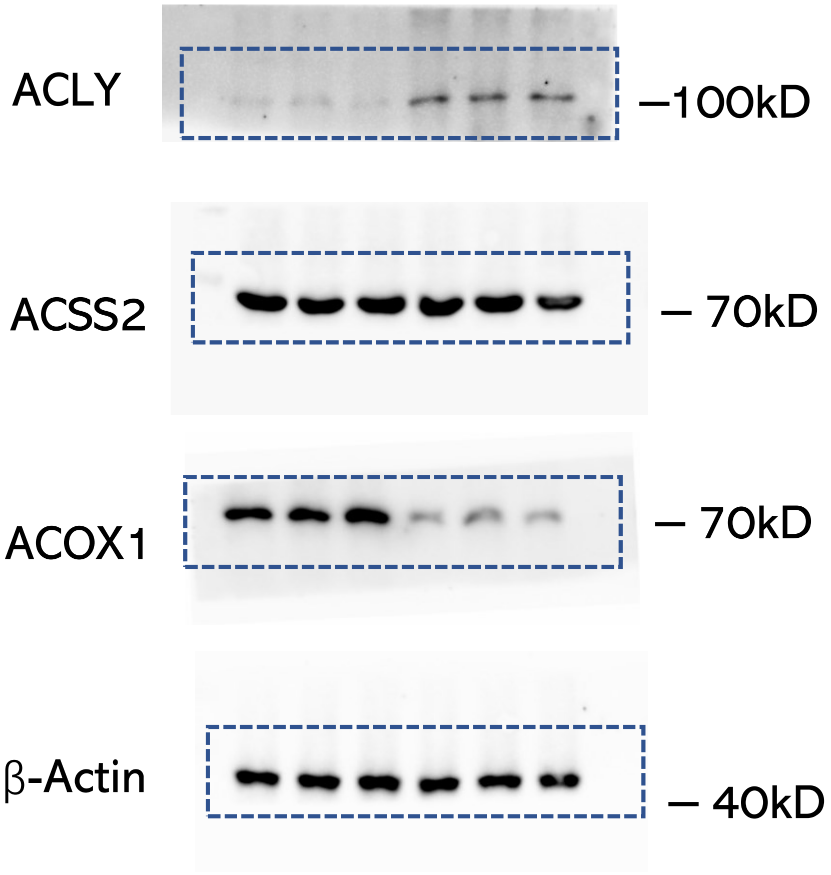
ACLY
—100kD
ACSS2
— 70kD
ACOX1
— 70kD
β-Actin
— 40kD

Fig. 3h

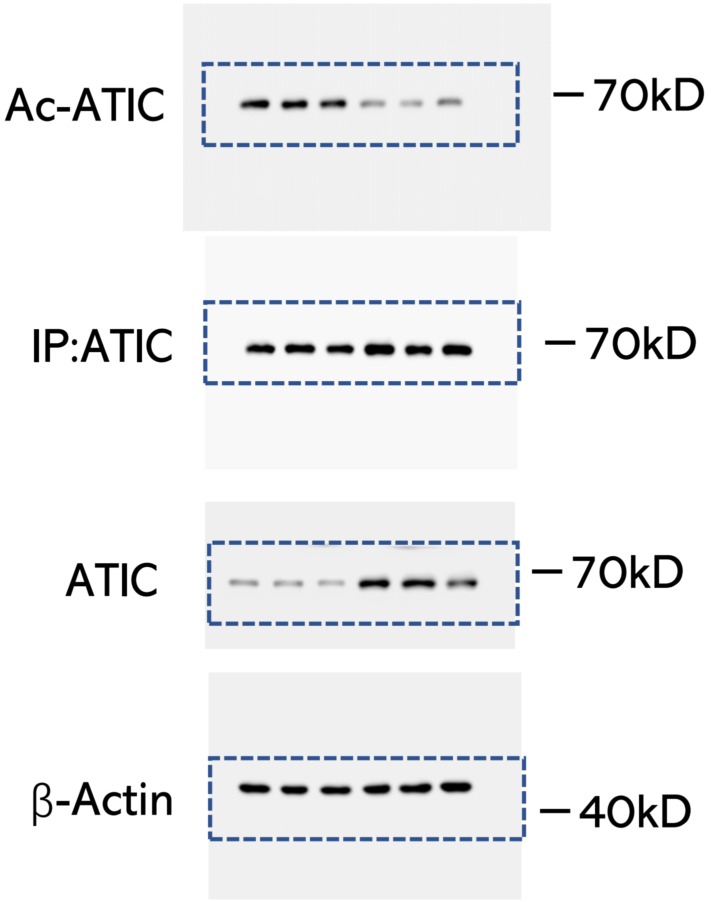
Ac-ATIC
—70kD
IP:ATIC
—70kD
ATIC
—70kD
β-Actin
—40kD

Fig. S3b

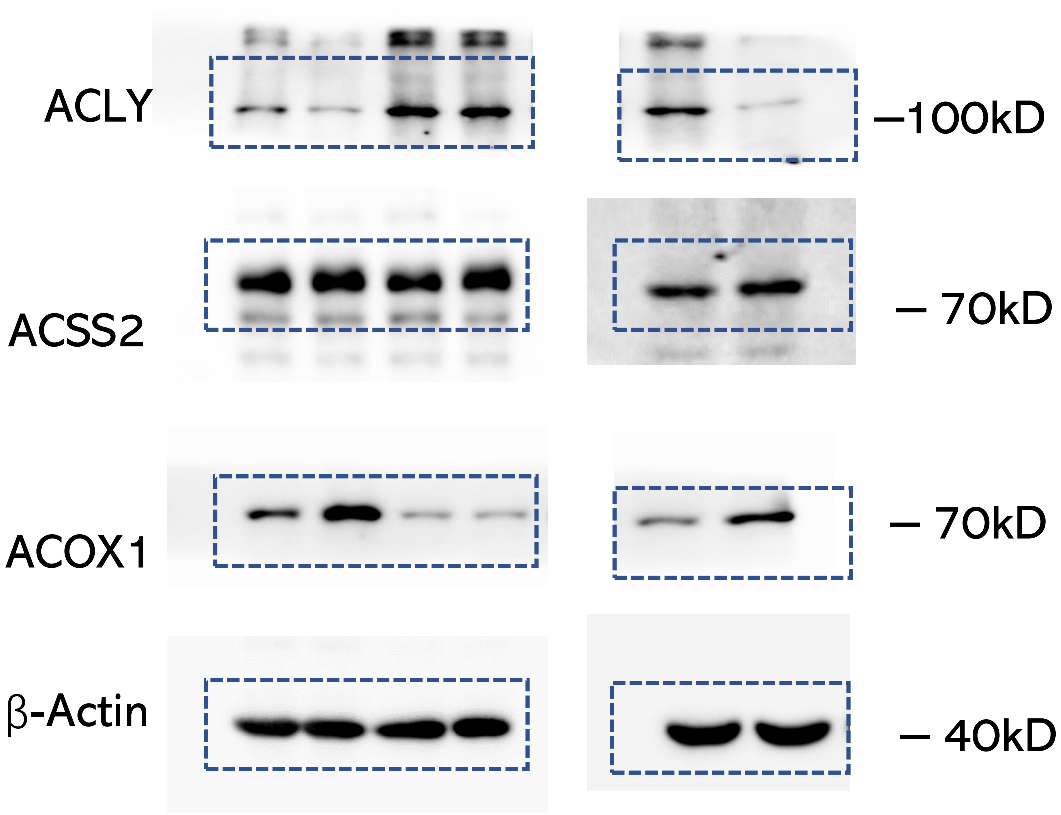

Fig. S3d

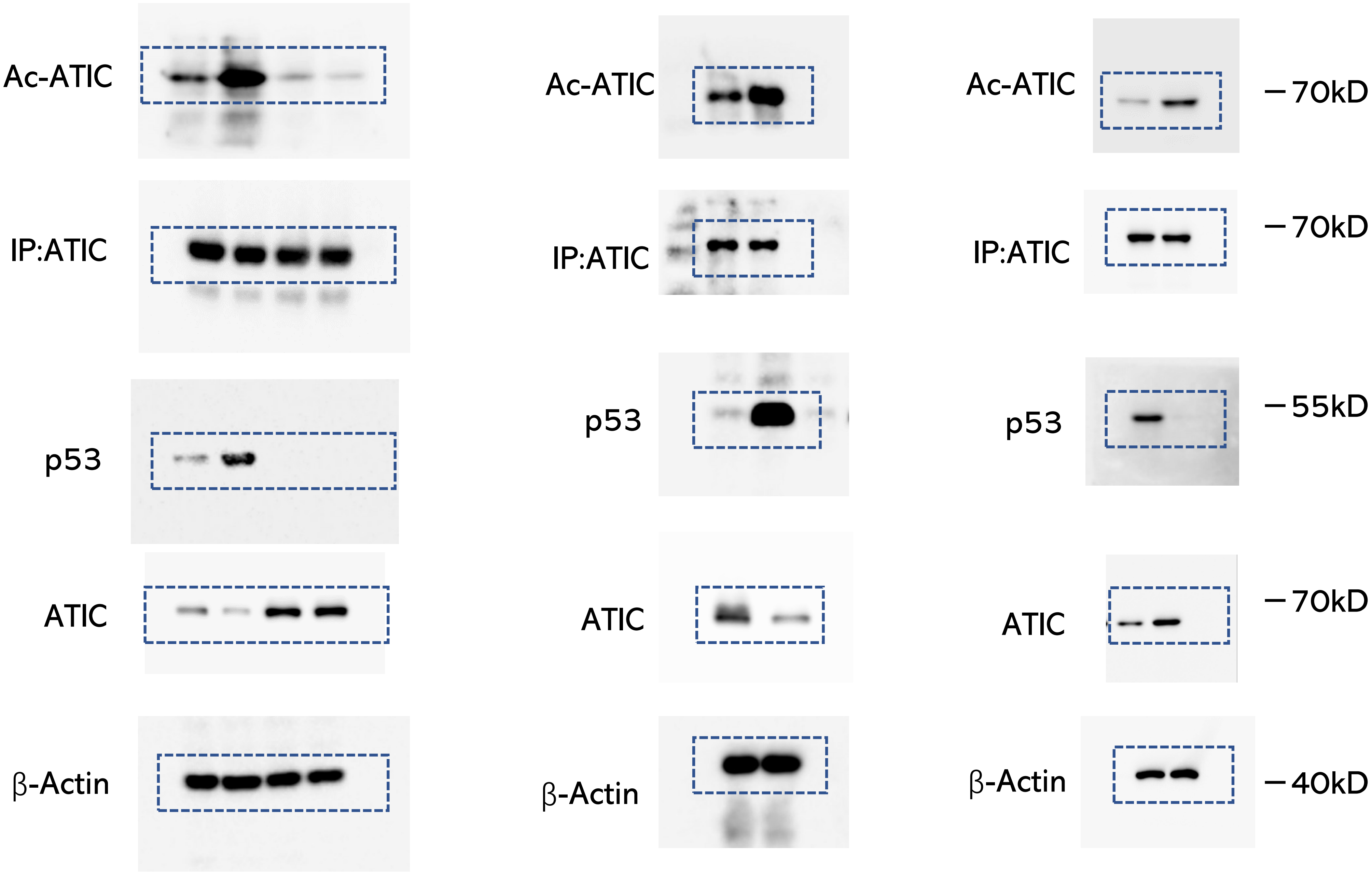
Ac-ATIC
IP:ATIC
p53
ATIC
β-Actin
Ac-ATIC
IP:ATIC
p53
ATIC
β-Actin
Ac-ATIC
IP:ATIC
p53
ATIC
β-Actin
70kD
70kD
55kD
70kD
40kD

Fig. S3e

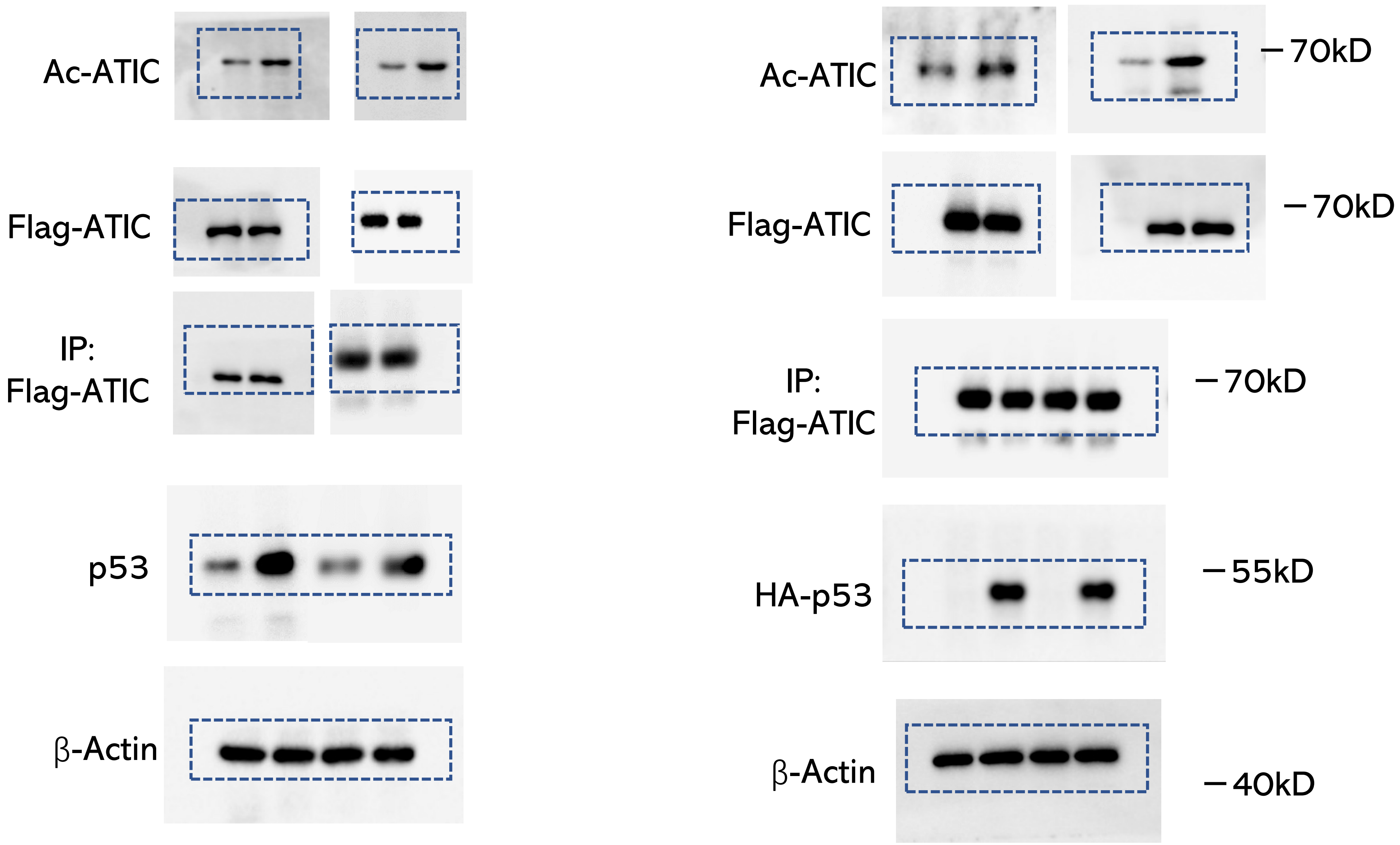
Ac-ATIC
Flag-ATIC
IP:
Flag-ATIC
p53
β-Actin
Ac-ATIC
70kD
Flag-ATIC
70kD
IP:
Flag-ATIC
70kD
HA-p53
55kD
β-Actin
40kD

Fig. S3f

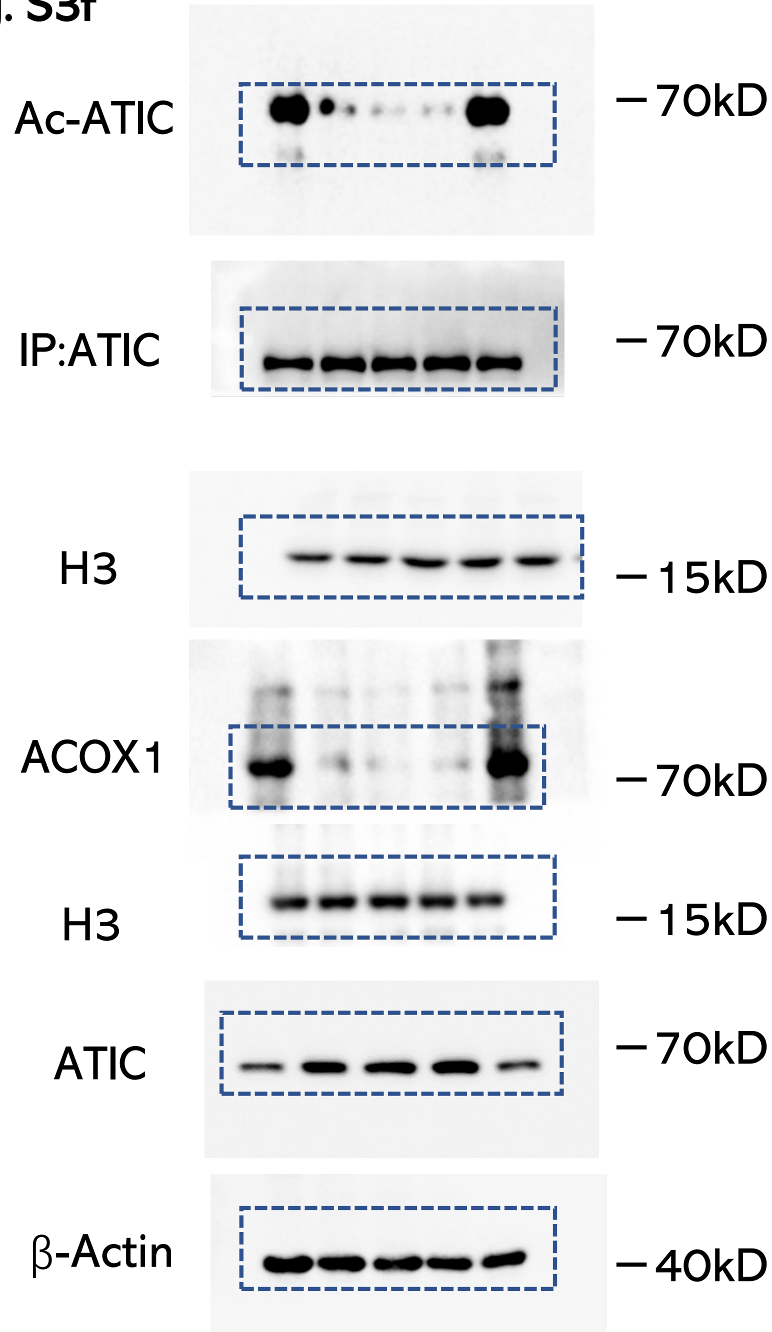

Fig. S3h

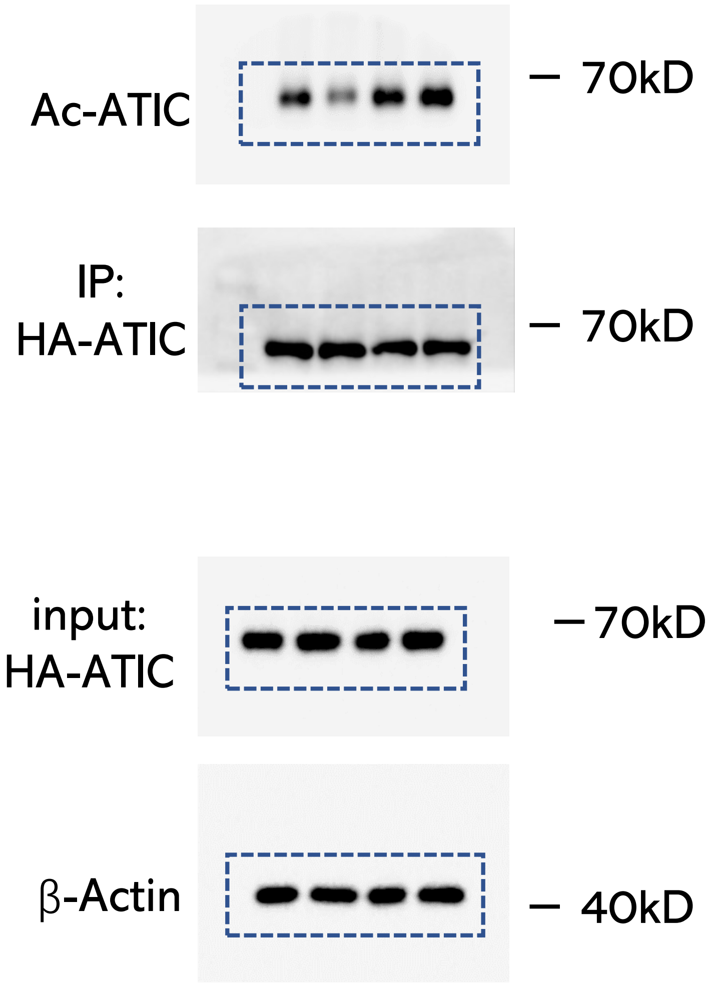

Fig. S3j

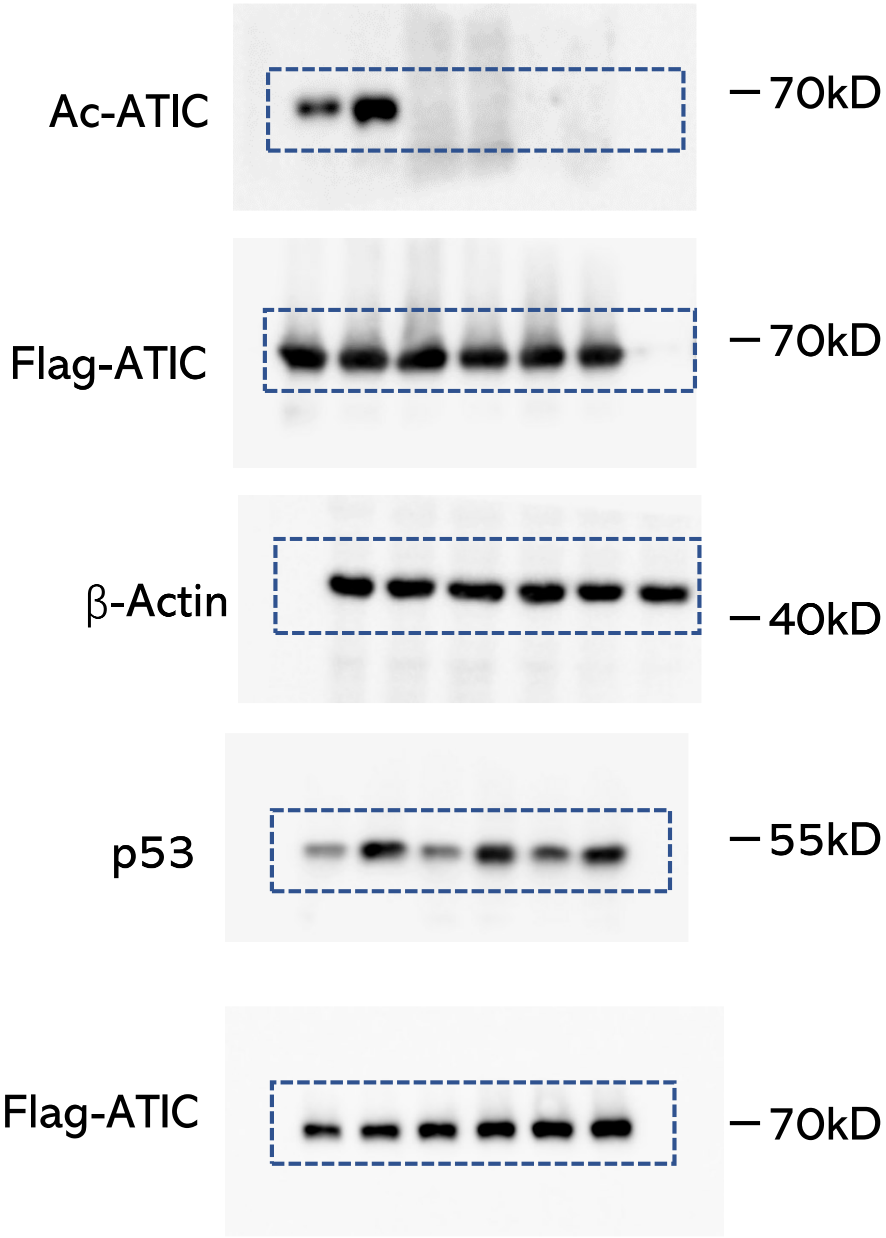

Ac-ATIC
70kD
Flag-ATIC
70kD
β-Actin
40kD
p53
55kD
Flag-ATIC
70kD

Fig. 4b

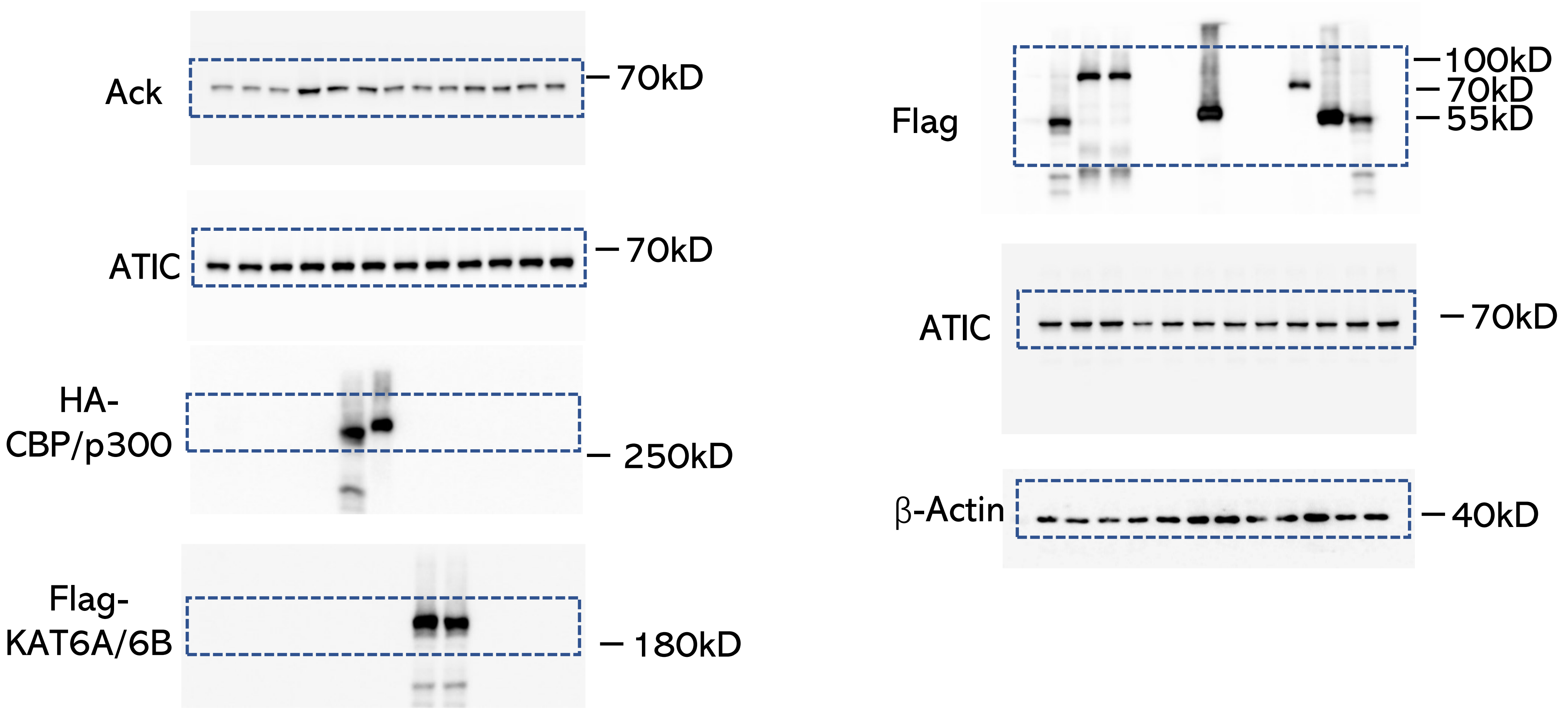
Ack
70kD
ATIC
70kD
HA-
CBP/p300
250kD
Flag-
KAT6A/6B
180kD
Flag
100kD
70kD
55kD
ATIC
70kD
β-Actin
40kD

Fig. 4c

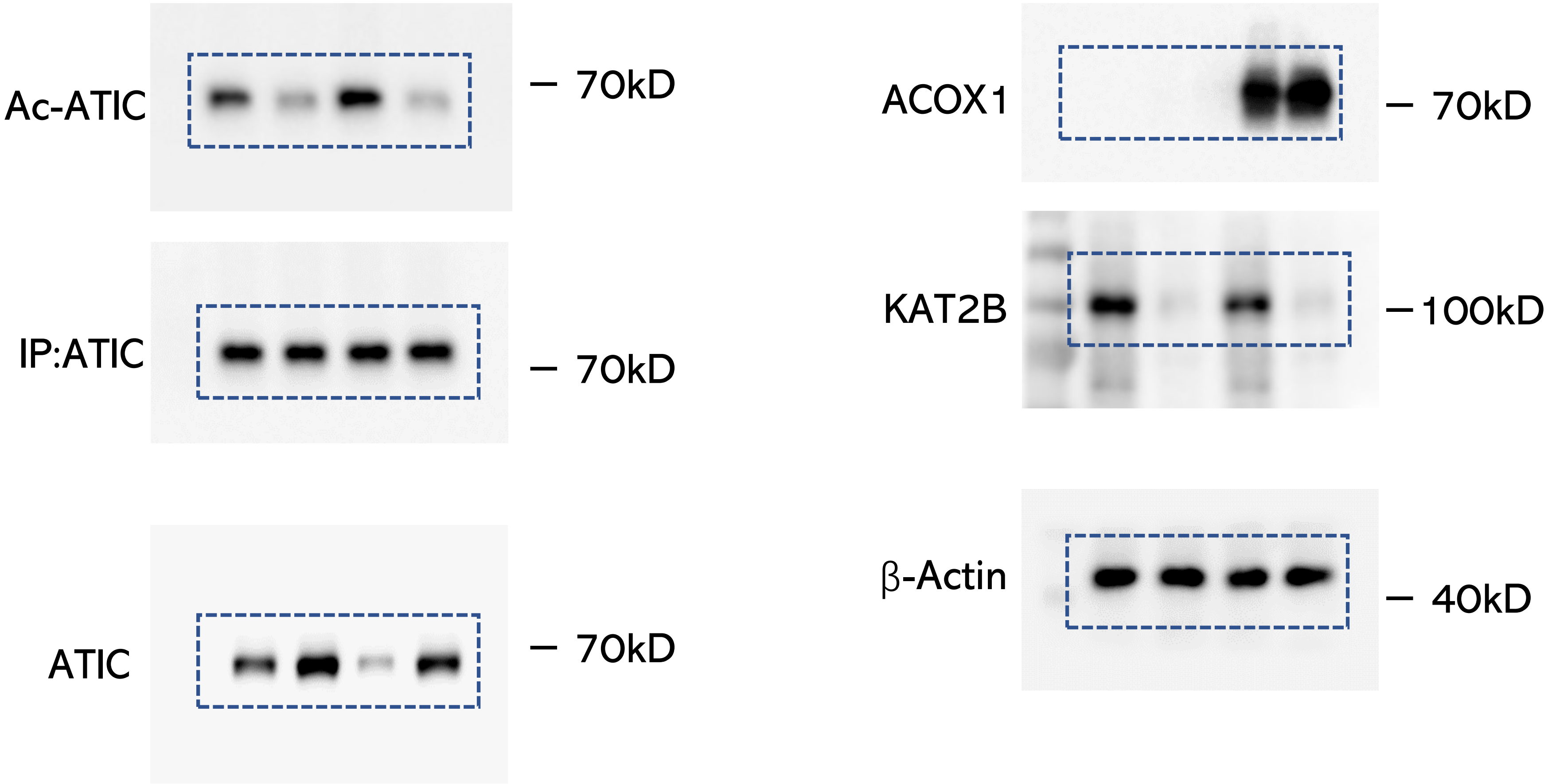
Ac-ATIC
— 70kD
IP:ATIC
— 70kD
ATIC
— 70kD
ACOX1
— 70kD
KAT2B
—100kD
β-Actin
— 40kD

Fig. 4d

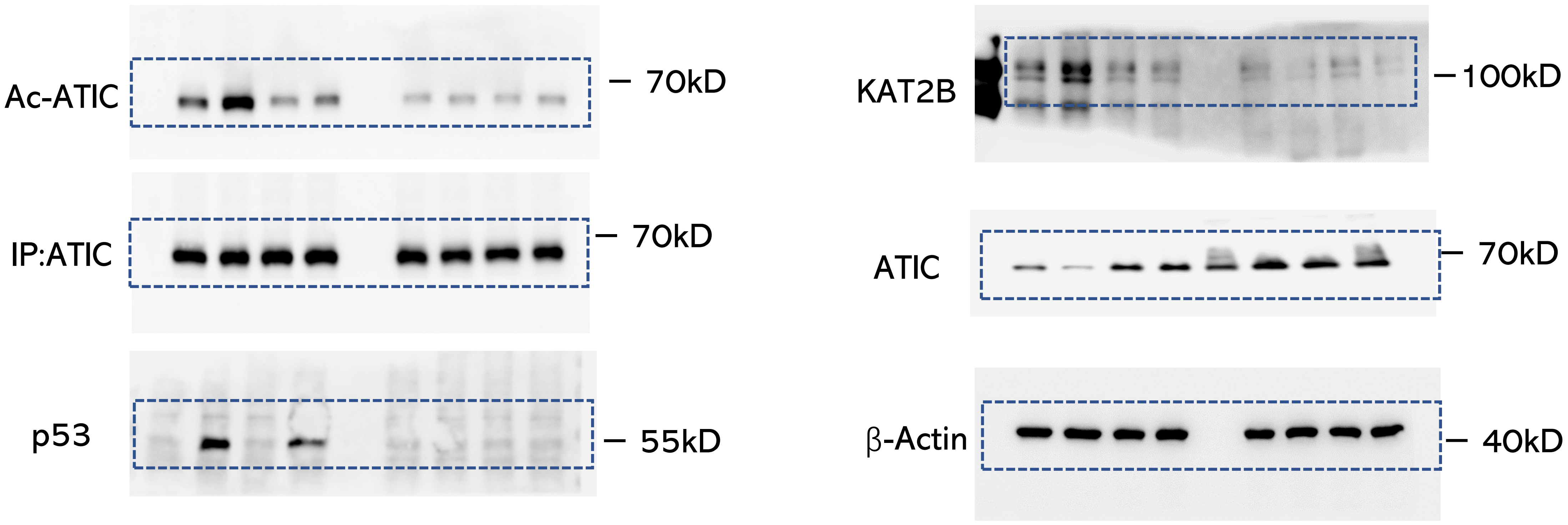

Ac-ATIC
70kD
IP:ATIC
70kD
p53
55kD
KAT2B
100kD
ATIC
70kD
β-Actin
40kD

Fig. 4e

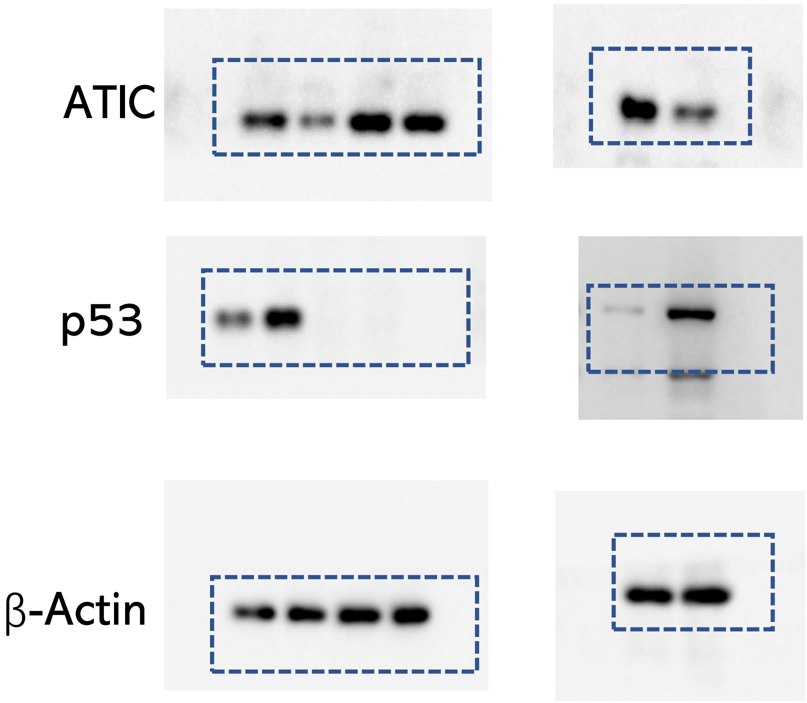

Fig. 4f

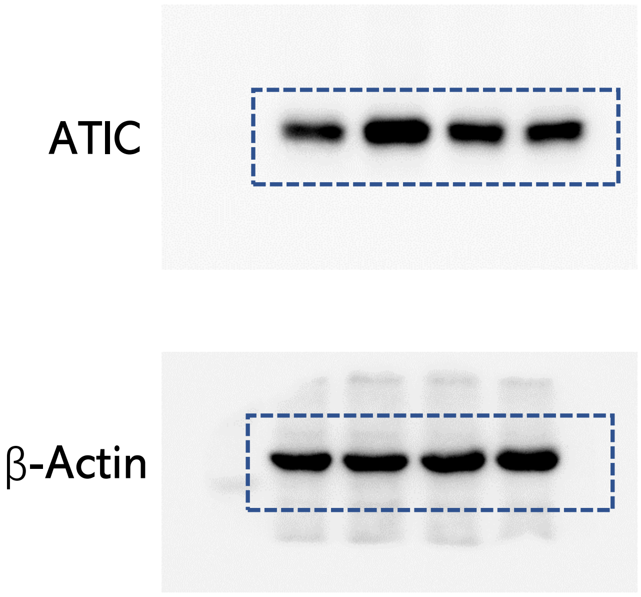

Fig. 4g

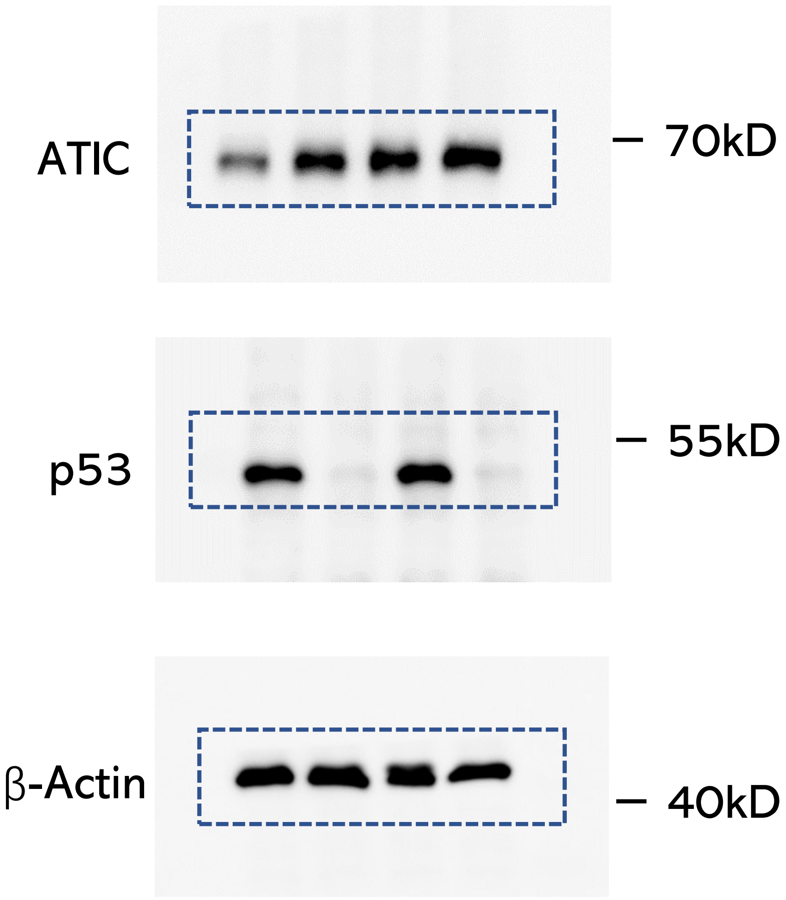

Fig. S4b

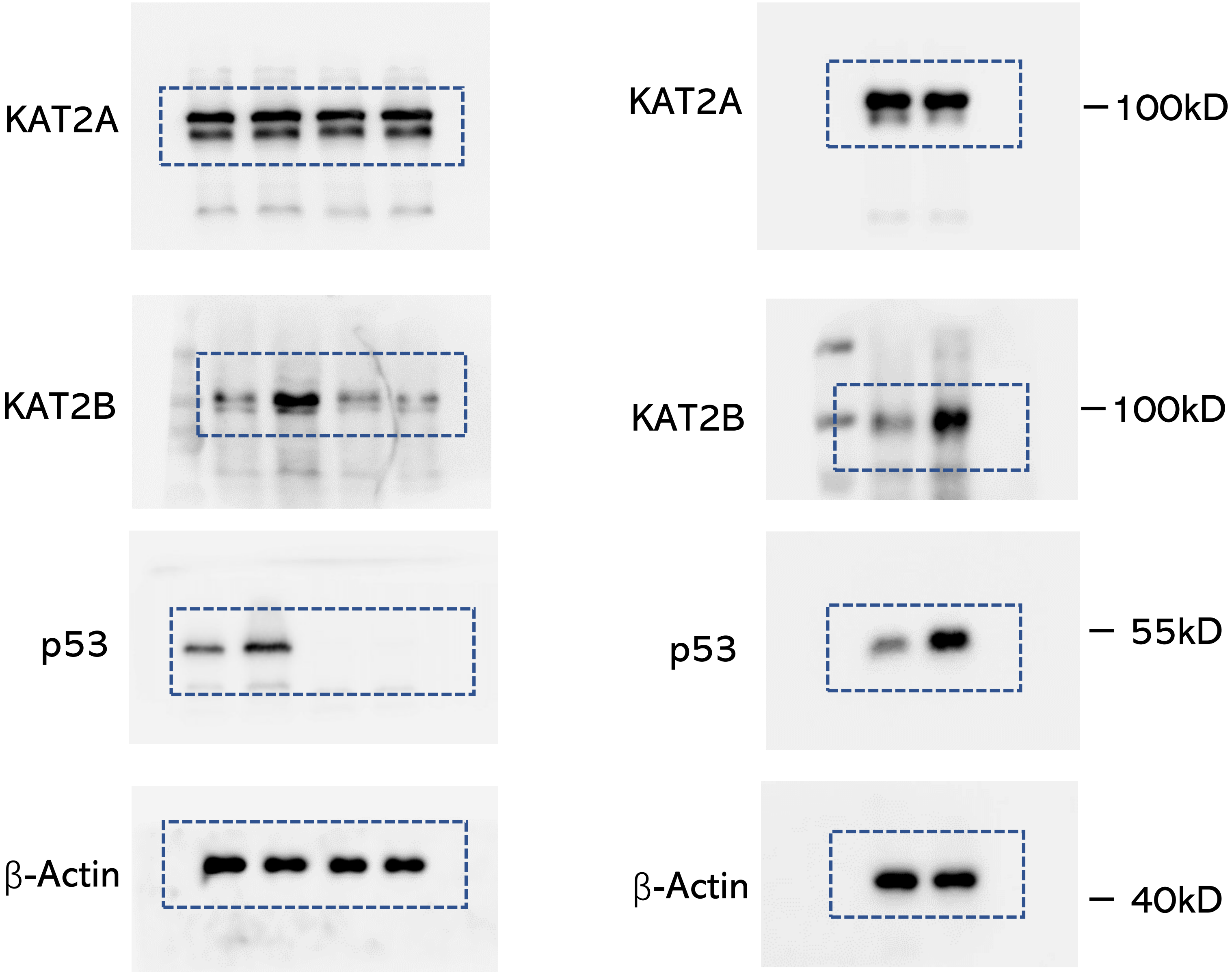

KAT2A
KAT2B
p53
β-Actin
KAT2A
100kD
KAT2B
100kD
p53
55kD
β-Actin
40kD

Fig. S4c

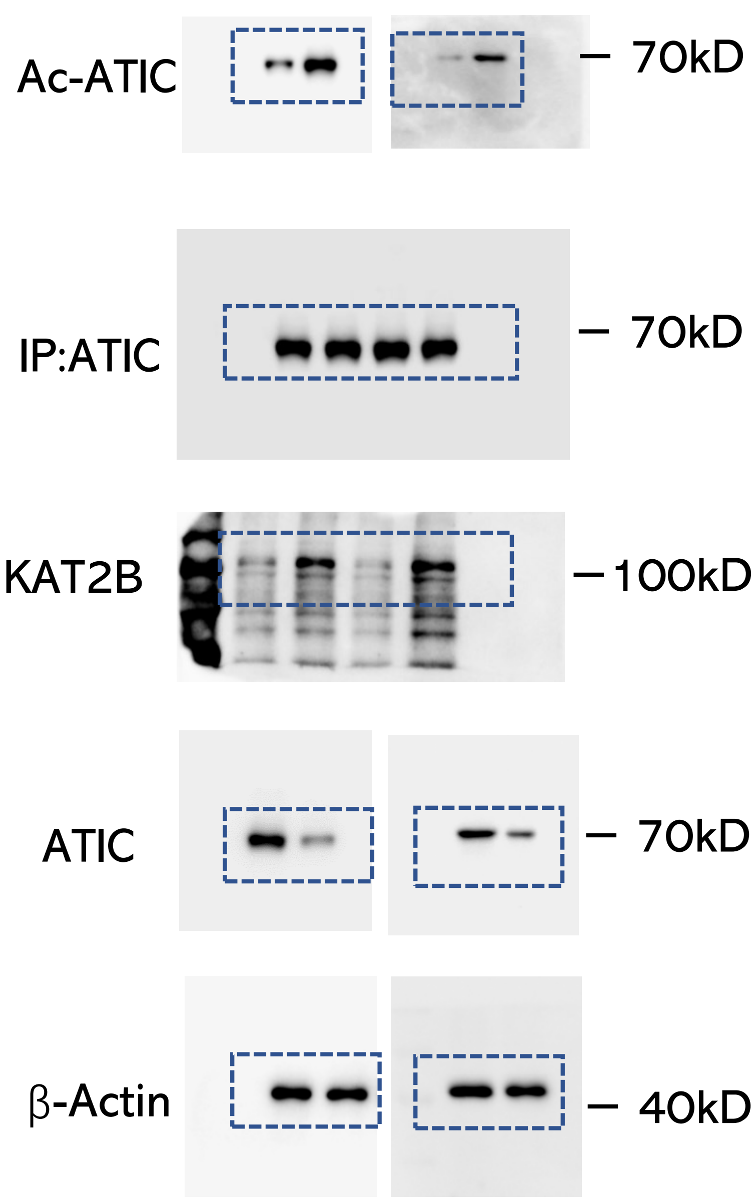

Fig. S4d

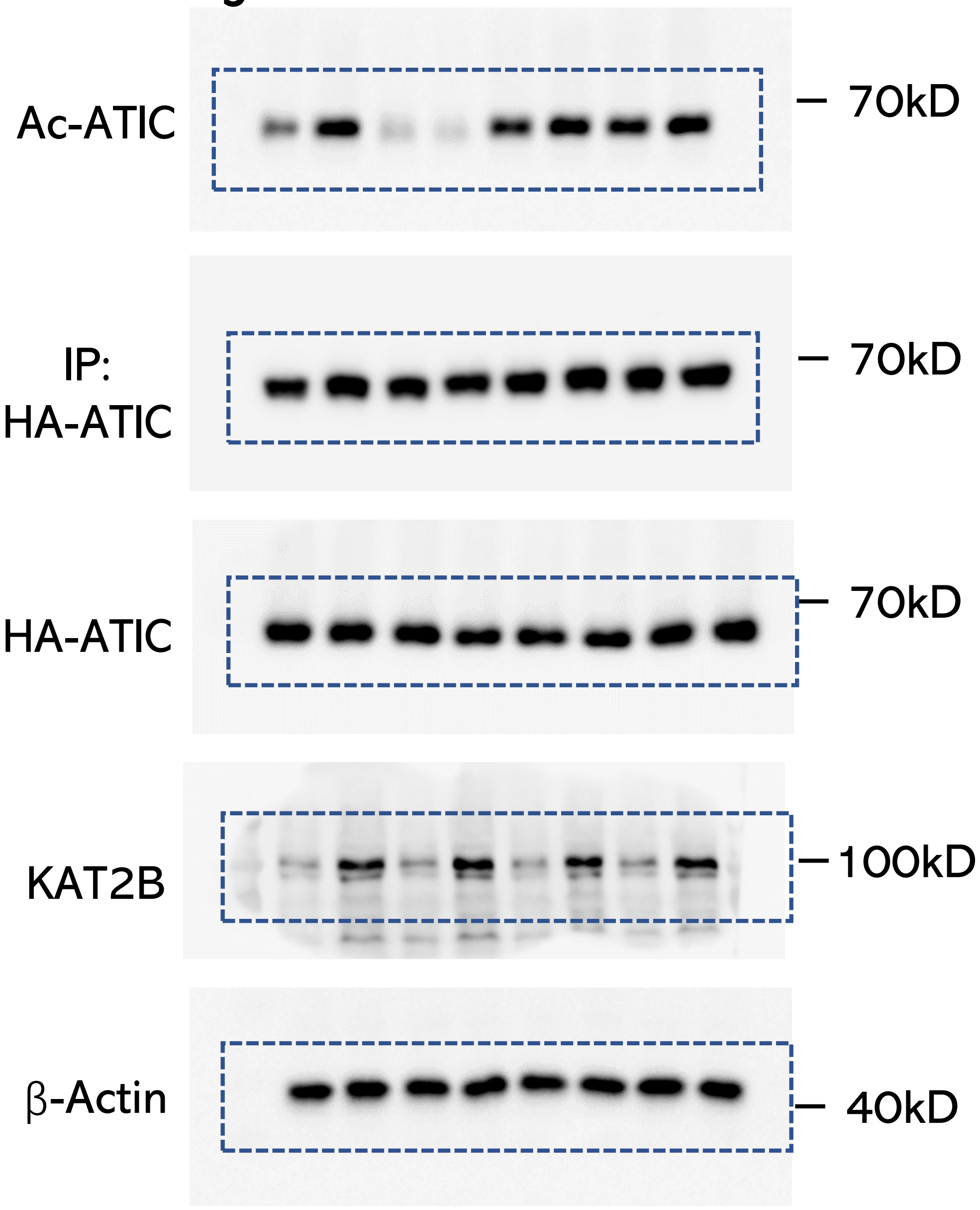

Fig. S4e

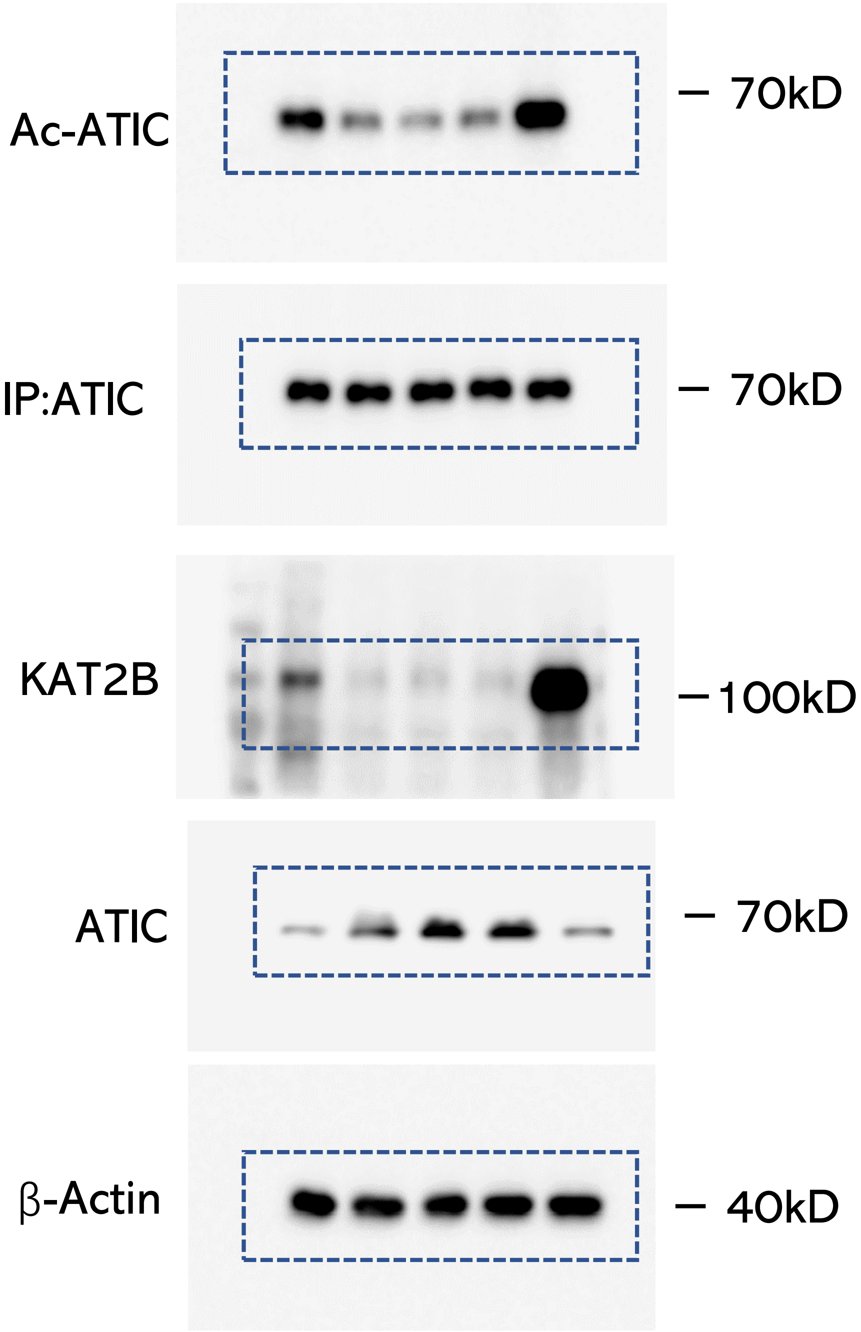

Fig. S4f

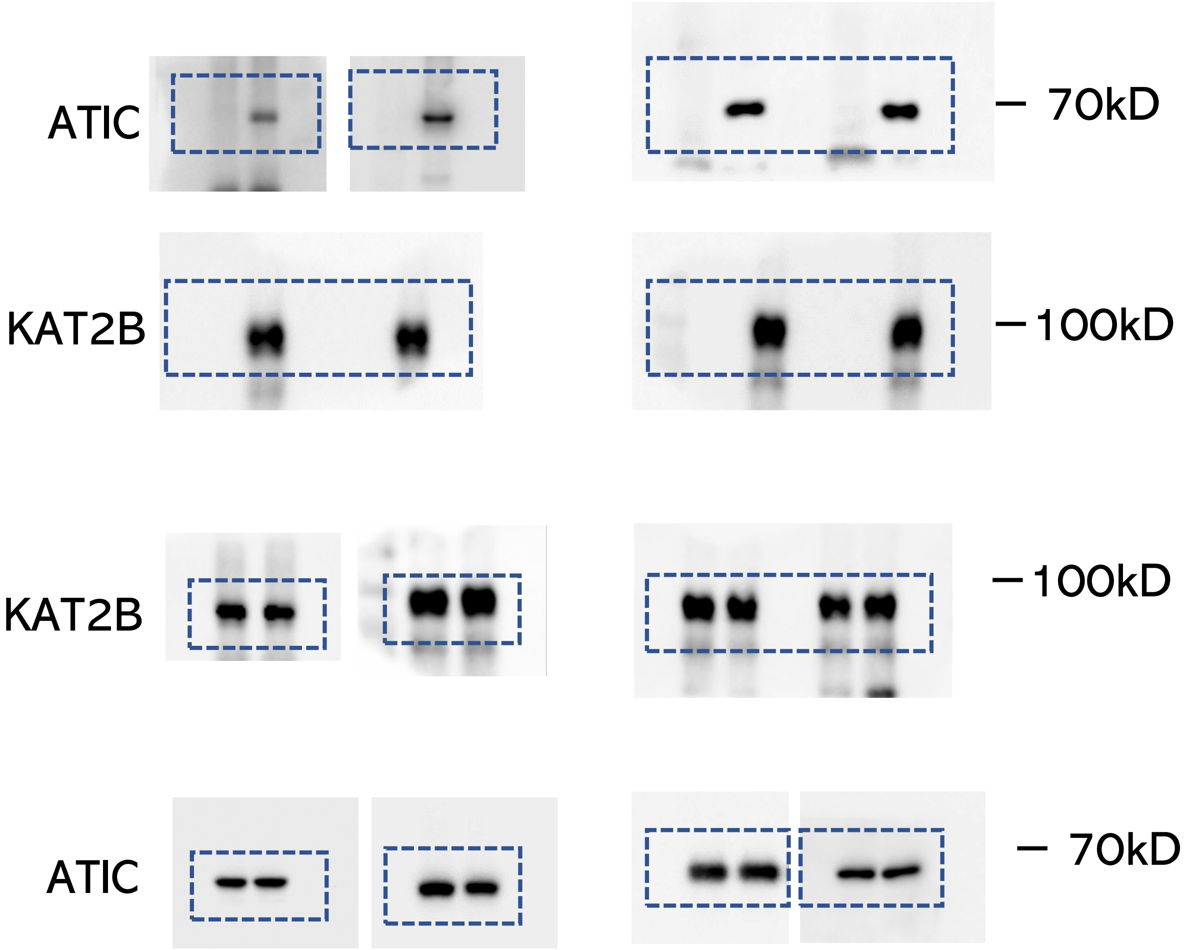

Fig. S4h

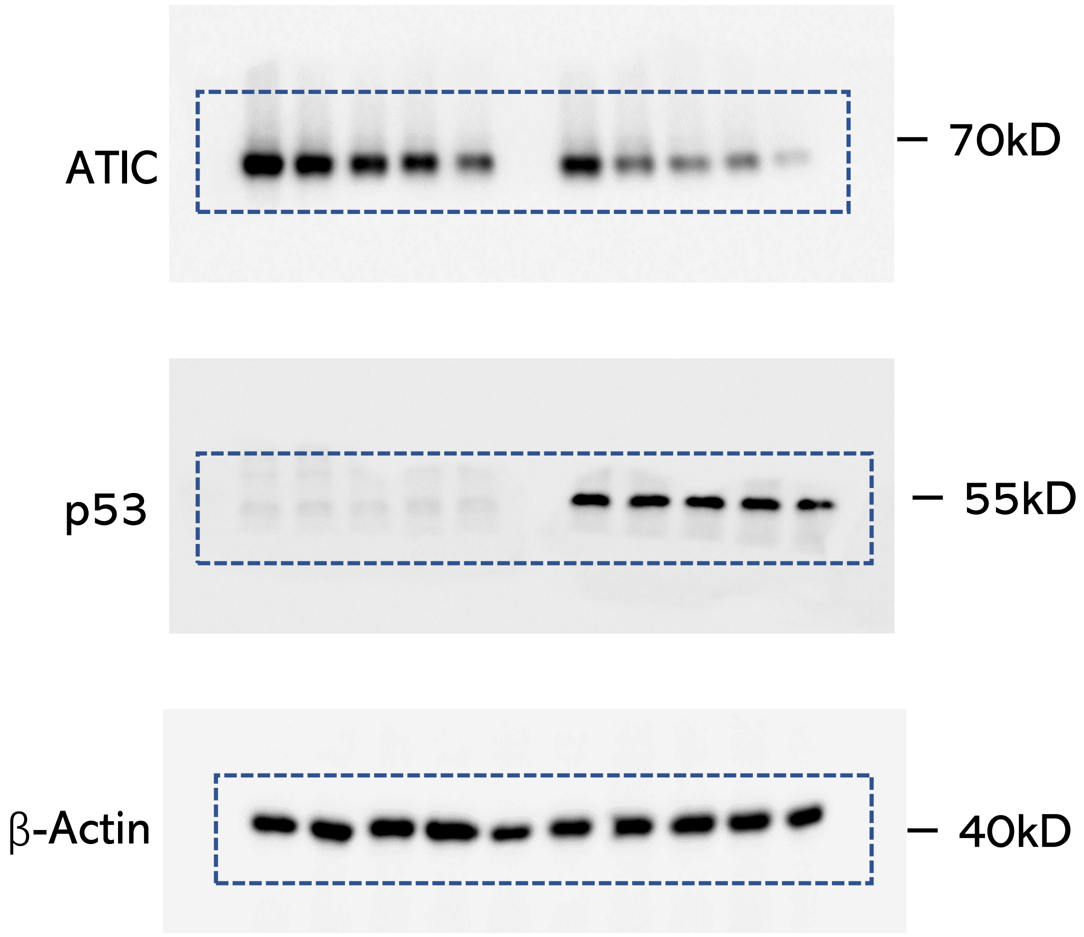

Fig. S4i

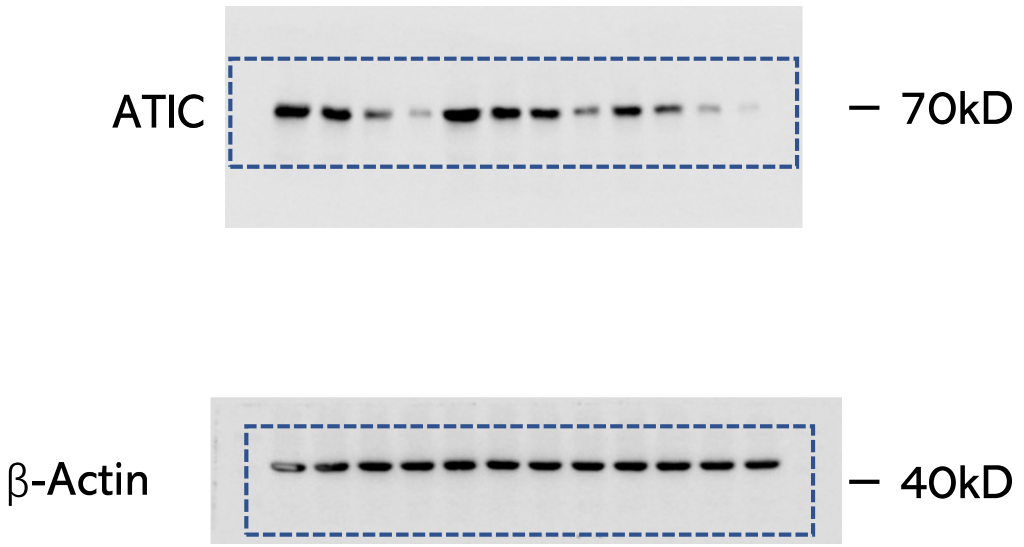

Fig. S4j

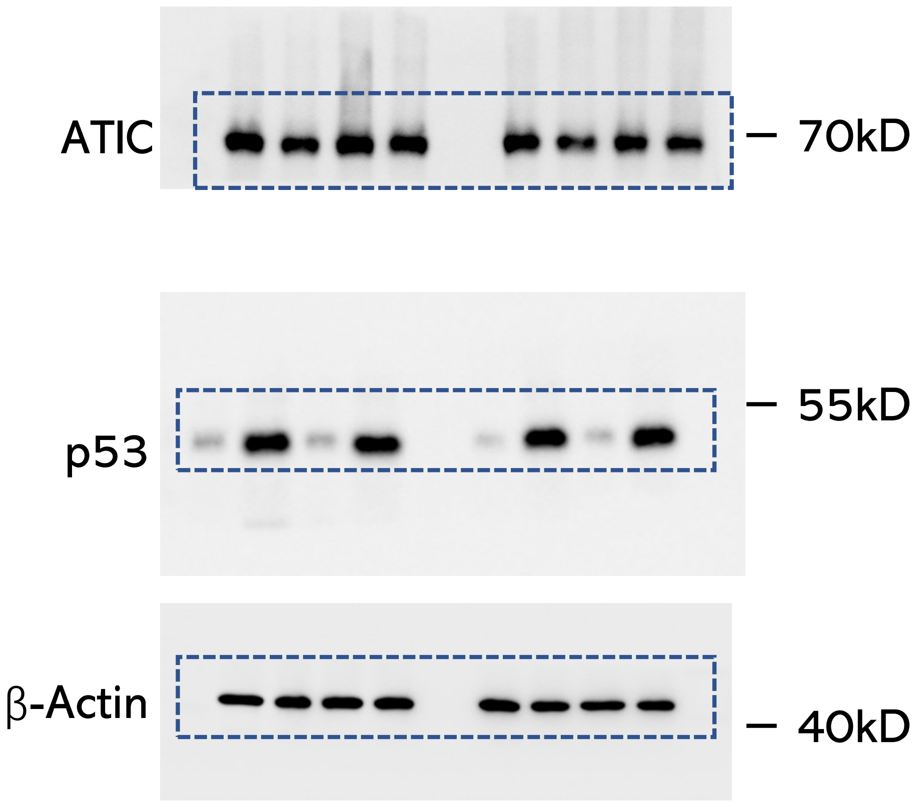

Fig. 5i

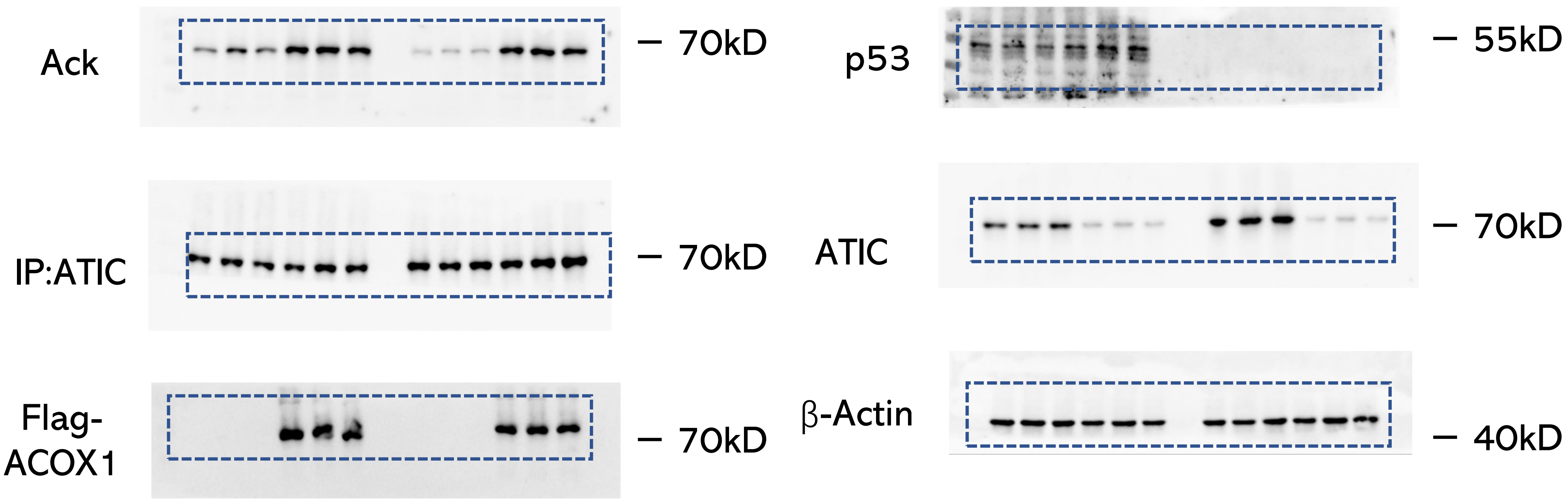

Ack
70kD
IP:ATIC
70kD
Flag-
ACOX1
70kD
p53
55kD
ATIC
70kD
β-Actin
40kD

Fig. S5b

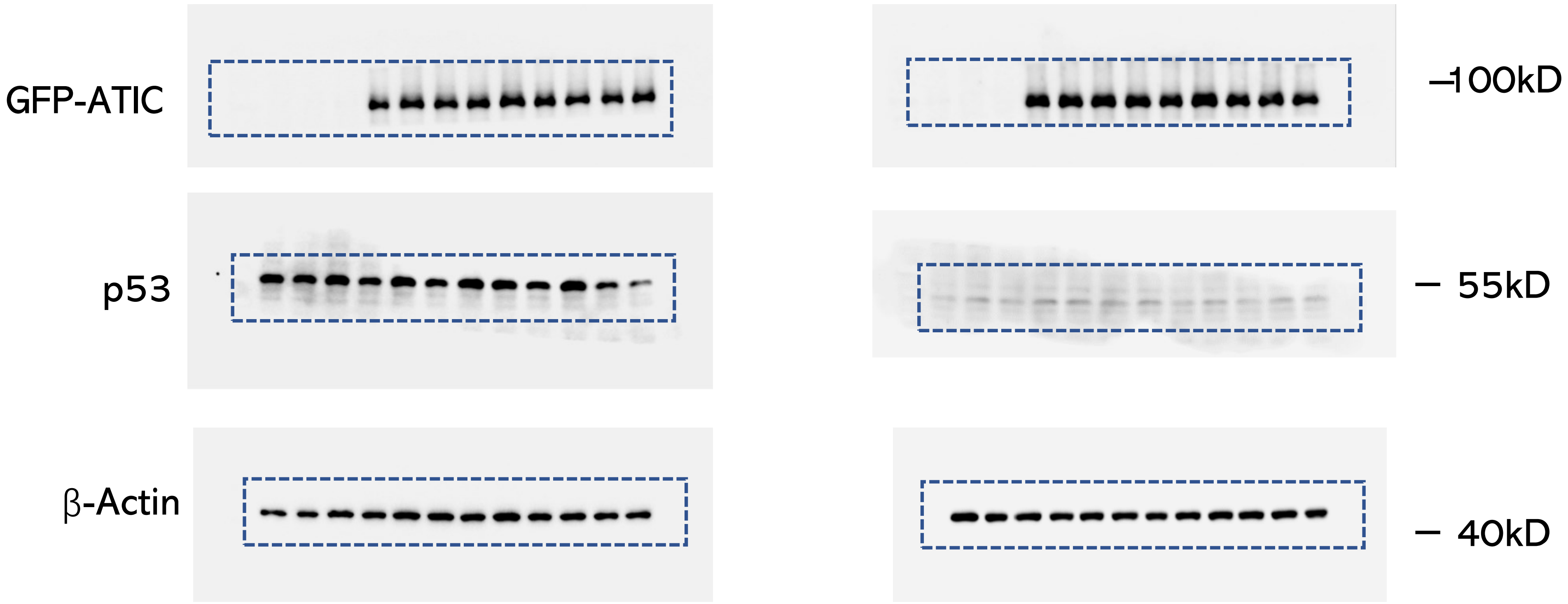

Fig. S6h

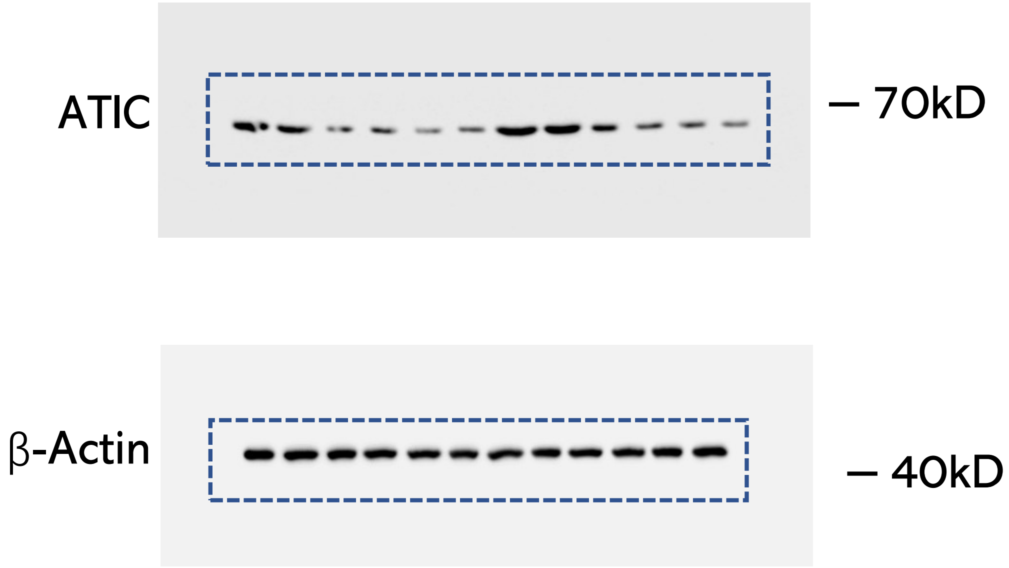

Fig. S7a **p53**

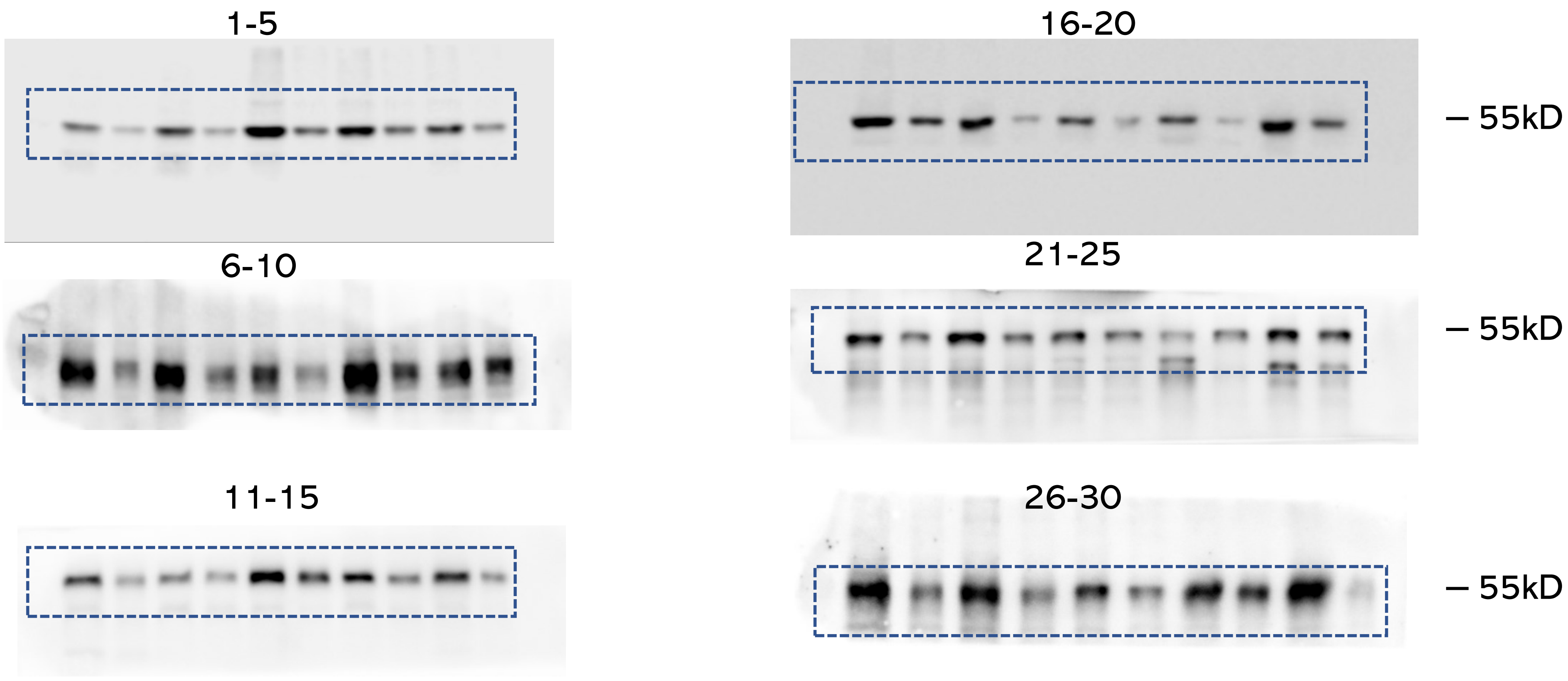

Fig. S7a

ACOX1

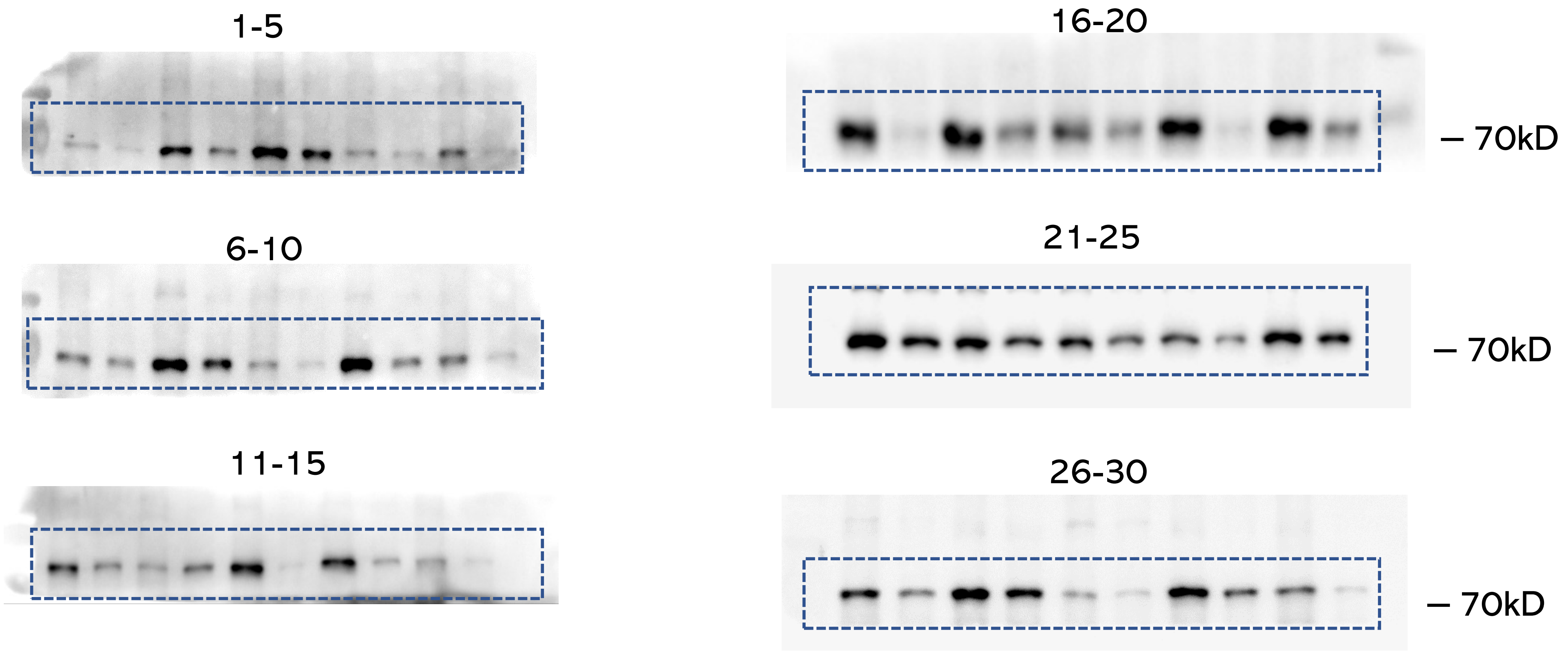

Fig. S7a

KAT2B

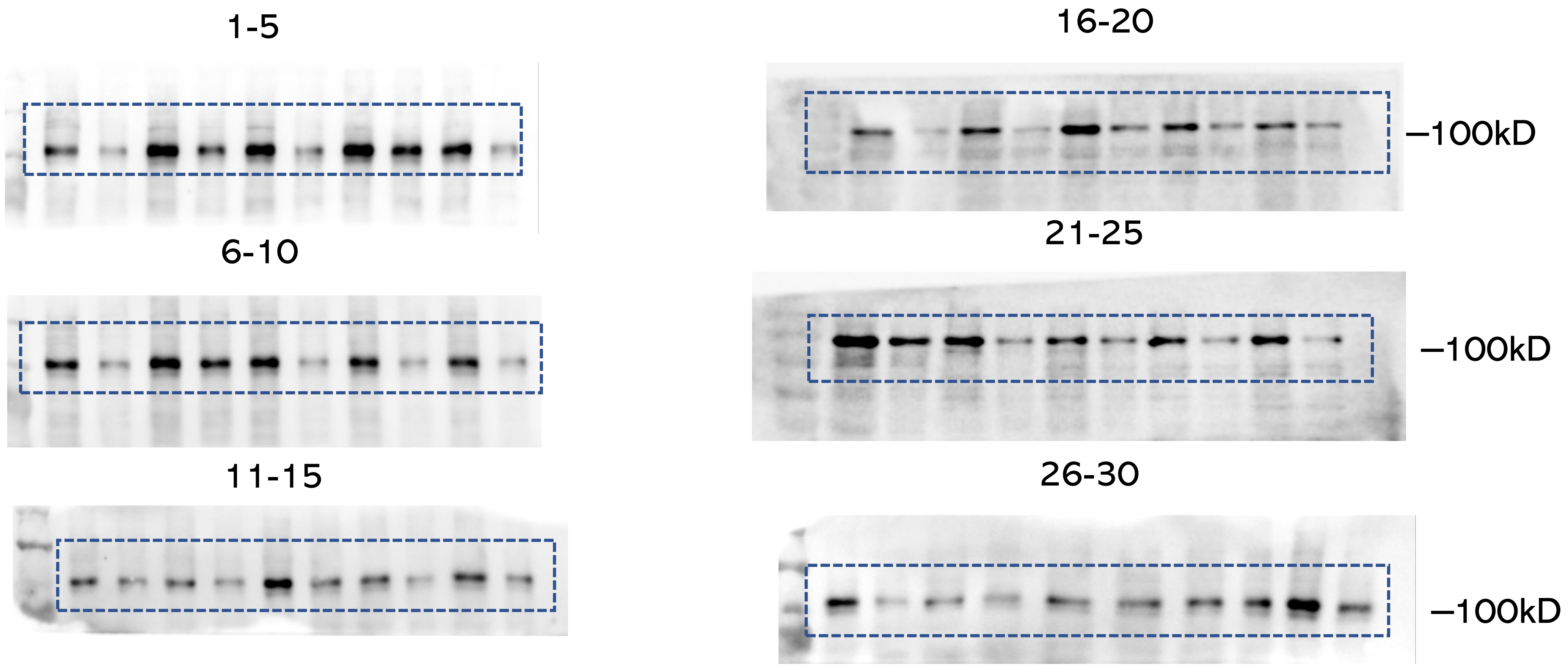

Fig. S7a ATIC

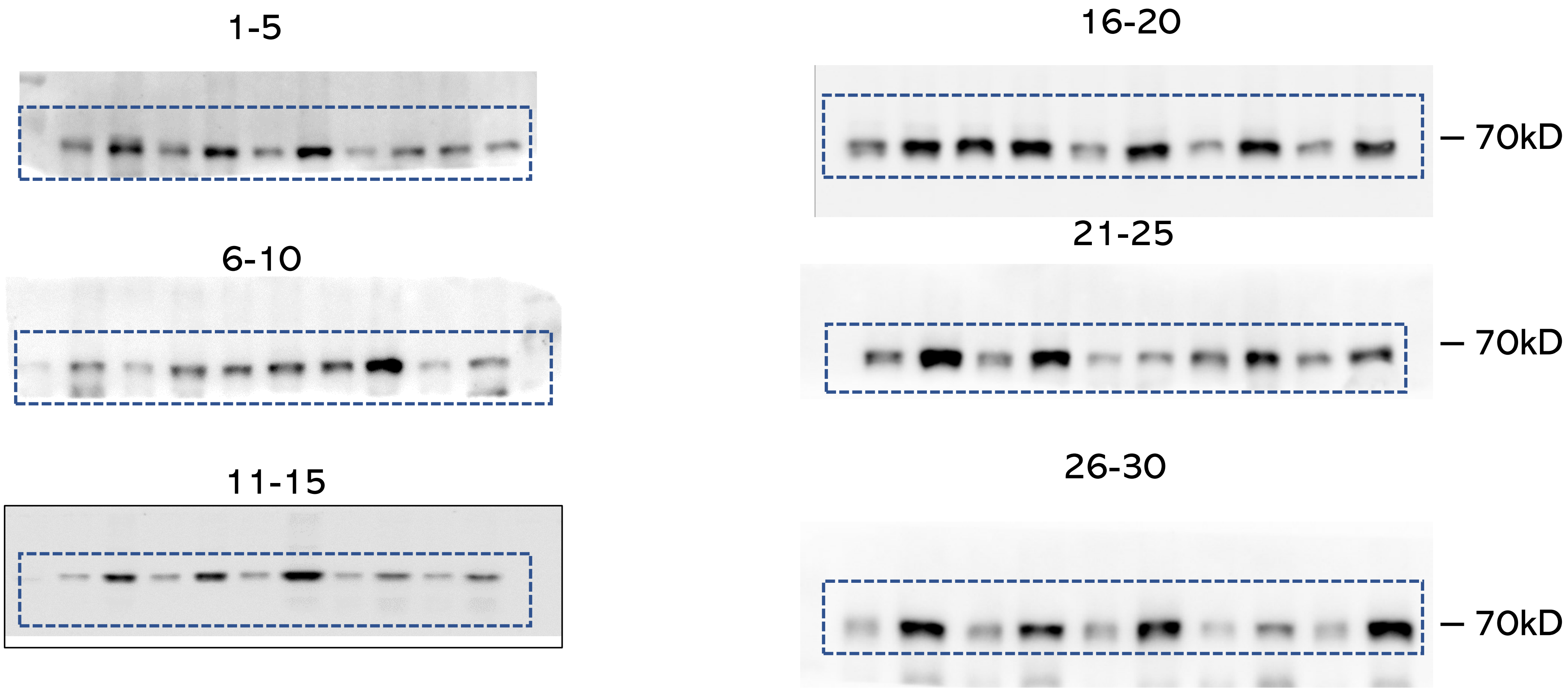

Fig. S7a Ac-ATIC

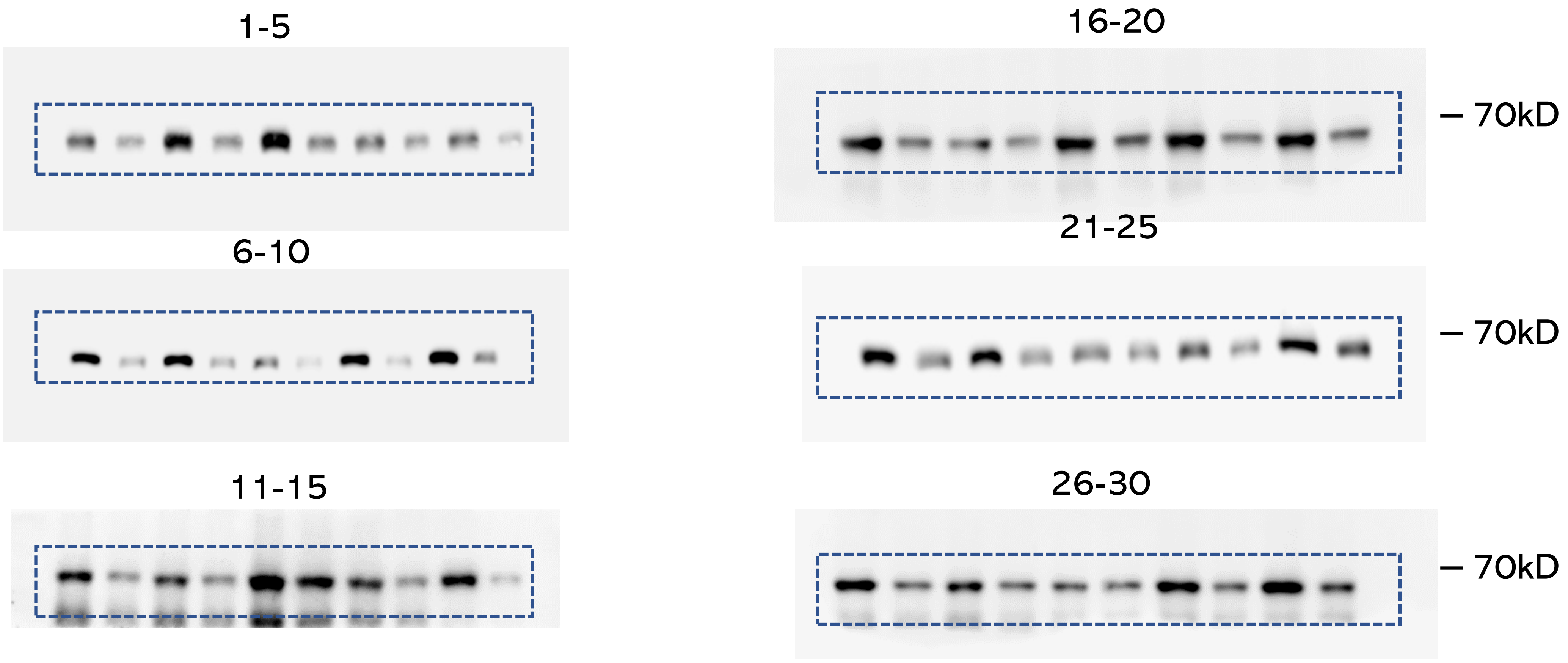

Fig. S7a IP: ATIC

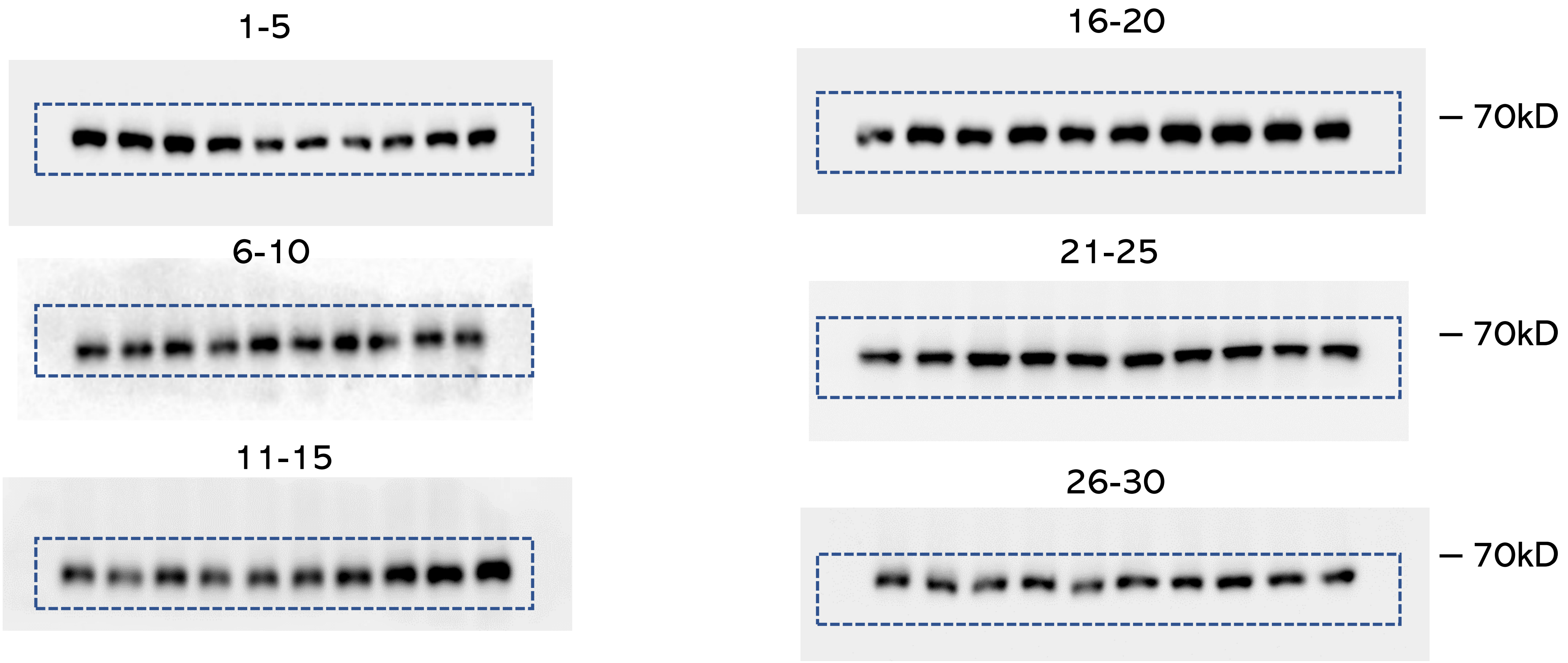

Fig. S7a

Actin

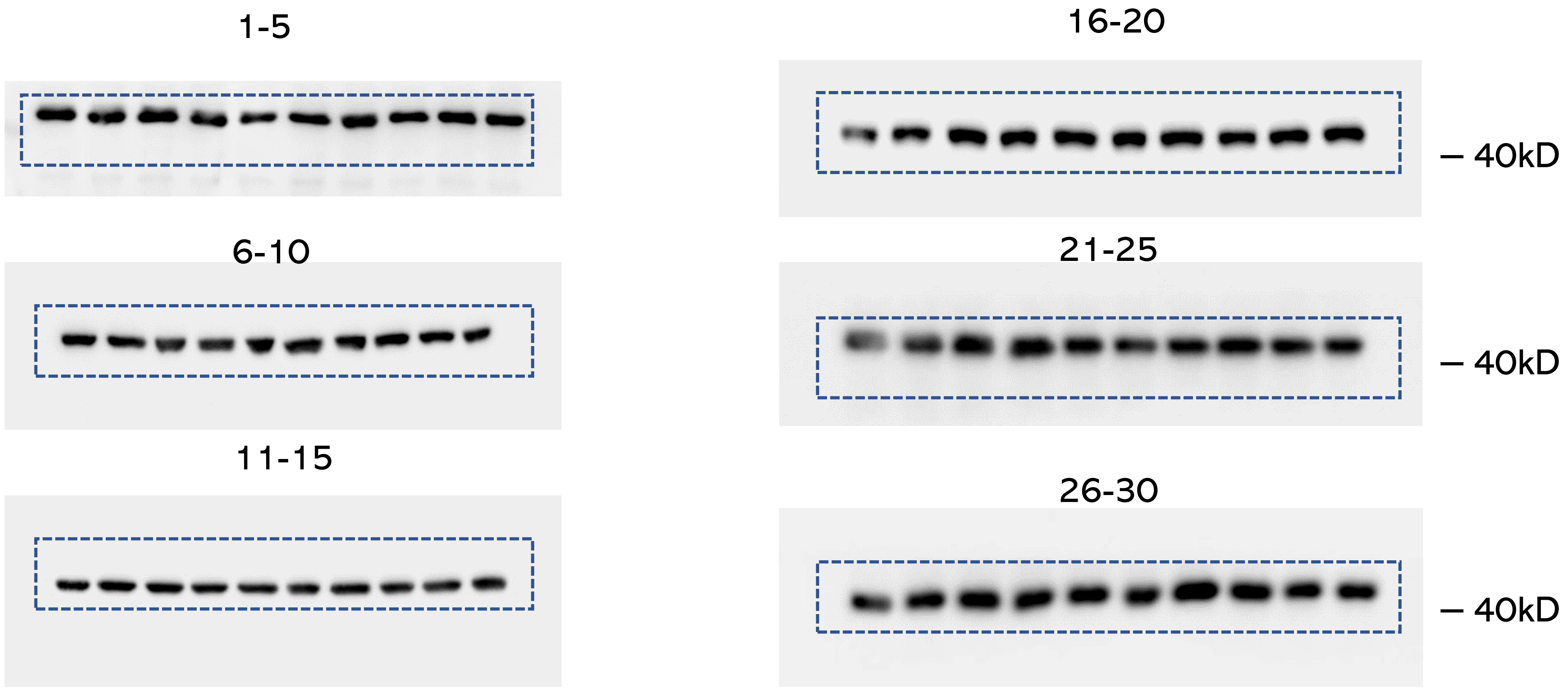

Supplement: Supplementary file 19 — Original data files [file 41419_2023_5625_MOESM19_ESM.pdf]
